# Mesenchymal stromal cells improve the transplantation outcome of CRISPR-Cas9 gene-edited human HSPCs

**DOI:** 10.1016/j.ymthe.2022.08.011

**Published:** 2022-08-17

**Authors:** Stefania Crippa, Anastasia Conti, Valentina Vavassori, Samuele Ferrari, Stefano Beretta, Silvia Rivis, Roberto Bosotti, Serena Scala, Stefania Pirroni, Raisa Jofra-Hernandez, Ludovica Santi, Luca Basso-Ricci, Ivan Merelli, Pietro Genovese, Alessandro Aiuti, Luigi Naldini, Raffaella Di Micco, Maria Ester Bernardo

**Affiliations:** 1 San Raffaele Telethon Institute for Gene Therapy (SR-TIGET), IRCCS San Raffaele Scientific Institute, 20132 Milan, Italy; 2 Laboratory of Tumor Inflammation and Angiogenesis, VIB-KULeuven, 3000 Leuven, Belgium; 3 Cagliari University-Hospital, 09100 Cagliari, Italy; 4 National Research Council, Institute for Biomedical Technologies, 20132 Milan, Italy; 5 Dana-Farber/Boston Children’s Cancer and Blood Disorders Center, Department of Pediatric Oncology, Harvard Medical School, Boston, MA 02115, USA; 6 Pediatric Immunohematology and Bone Marrow Transplantation Unit, San Raffaele Scientific Institute, 20132 Milan, Italy; 7 ^“^Vita Salute” San Raffaele University, 20132 Milan, Italy

**Keywords:** mesenchymal stromal cells, hematopoietic support, hematopoietic stem and progenitor cells, genome editing, DNA-damage response

## Abstract

Mesenchymal stromal cells (MSCs) have been employed *in vitro* to support hematopoietic stem and progenitor cell (HSPC) expansion and *in vivo* to promote HSPC engraftment. Based on these studies, we developed an MSC-based co-culture system to optimize the transplantation outcome of clustered regularly interspaced short palindromic repeats (CRISPR)-Cas9 gene-edited (GE) human HSPCs. We show that bone marrow (BM)-MSCs produce several hematopoietic supportive and anti-inflammatory factors capable of alleviating the proliferation arrest and mitigating the apoptotic and inflammatory programs activated in GE-HSPCs, improving their expansion and clonogenic potential *in vitro*. The use of BM-MSCs resulted in superior human engraftment and increased clonal output of GE-HSPCs contributing to the early phase of hematological reconstitution in the peripheral blood of transplanted mice. In conclusion, our work poses the biological bases for a novel clinical use of BM-MSCs to promote engraftment of GE-HSPCs and improve their transplantation outcome.

## Graphical abstract

**Figure:**
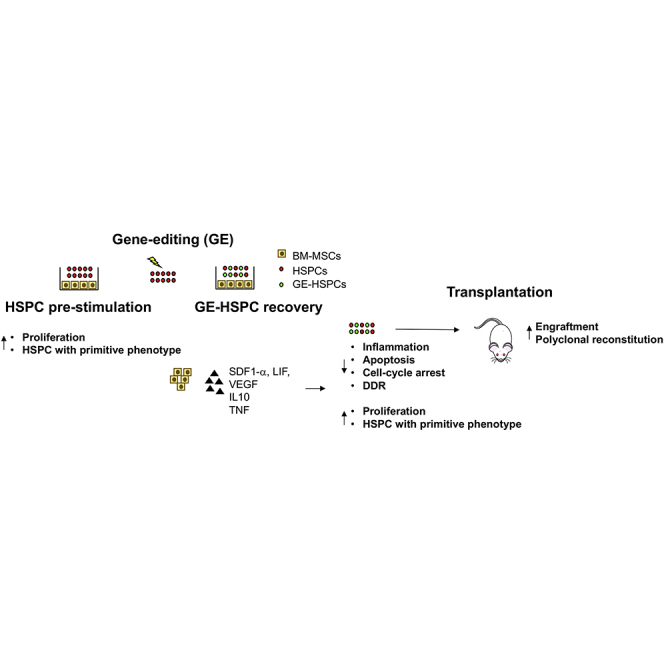

## Teaser

An MSC-based co-culture system is developed to protect HSPCs from the side effects of gene editing and improve the gene-edited HSPC transplantation outcome.

## Introduction

Mesenchymal stromal cells (MSCs) are crucial elements in the bone marrow (BM) niche, where they are physically associated with hematopoietic stem and progenitor cells (HSPCs) and regulate their homeostasis mainly through the release of paracrine factors.^1^^,^^2^^,^^3^

*Ex vivo* expanded human MSCs^4^ have been employed in pre-clinical models of HSC transplantation (HSCT) and phase I/II clinical trials to favor the engraftment of transplanted HSPCs and their hematopoietic reconstitution.^5^^,^^6^^,^^7^^,^^8^^,^^9^ Despite transplanted MSCs not persisting long term,^10^ previous works indicate that the MSC secretion of anti-inflammatory molecules reduces the inflammatory response to pre-transplant conditioning, rendering the BM niche a more favorable environment for the engraftment of transplanted HSPCs.^11^^,^^12^^,^^13^

In addition to their *in vivo* use, MSCs were extensively studied to enhance the *ex vivo* expansion of umbilical cord blood (UCB)-derived HSPCs before HSCT. MSCs improve the outcome of UCB-HSPC transplantation due to their ability to secrete hematopoietic supportive factors^14^^,^^15^^,^^16^ and directly interact with HSPCs to promote survival and proliferation while preventing their differentiation.^17^ Moreover, several works indicated that the use of stromal cells in co-culture with HSPCs also improves the gene-transfer efficiency in long-term repopulating HSPCs by producing a supportive stromal matrix and releasing stromal factors.^18^^,^^19^^,^^20^

Autologous HSPC gene therapy (GT) with corrective transgenes delivered by retro/lentiviral vectors has recently become a curative treatment for different inherited genetic disorders.^21^^,^^22^^,^^23^^,^^24^^,^^25^ Meanwhile, this rapidly evolving field is already witnessing the development of a new generation of advanced genetic therapies based on gene editing (GE) technologies, which employ programmable nucleases, such as clustered regularly interspaced short palindromic repeats (CRISPR)-Cas9, to achieve locus-specific gene correction, thus minimizing the risk of genome-wide vector integration.^26^ However, the efficiency of GE strategies based on homology-directed repair (HDR) remains limited in long-term (LT) repopulating HSPCs due to low proficiency of homologous recombination, limited permissiveness to the delivery of the DNA-repair template,^27^^,^^28^^,^^29^^,^^30^^,^^31^^,^^32^^,^^33^^,^^34^ and the induction of cellular responses hampering the proliferative capacity of edited cells.^35^ These constrains lead to a restricted yield of edited HSPCs and limit the number of LT repopulating HSPCs edited by HDR available for transplantation, which could cause delayed engraftment and increased risk of graft failure in patients.

Based on these premises, the identification of novel and effective strategies to further improve the *ex vivo* maintenance/expansion and/or the *in vivo* engraftment of GE-HSPCs is a critical requirement for successful clinical translation of GE.

Previous work demonstrated that the transient inhibition of p53 increases the yield of clonogenic and repopulating GE-HSPCs.^35^ However, a multifactorial approach would better control the distinct signals activated in HSPCs upon nuclease-induced double-strand breaks (DSBs), known to induce a protracted DNA-damage response (DDR) activation and proliferation delay.

For this reason, we performed CRISPR-Cas9 GE in human HSPCs, and we employed an MSC-based two-dimensional (2D) co-culture system to increase the number and the fitness of gene-edited HSPCs available for transplantation. We based our strategy on the critical role of stromal cells in supporting cell expansion and gene transfer into primitive HSPCs and considering their anti-inflammatory properties mediated by the secretion of multiple paracrine factors.^14^^,^^15^^,^^16^^,^^18^^,^^19^ In this study, we investigate for the first time the supportive activity of MSCs on GE HSPCs, demonstrating that MSCs attenuate the proliferation block and the inflammatory response associated with the GE procedure in HSPCs, resulting in an improved transplantation outcome of GE-HSPCs.

The results of our work provide the biological basis for the clinical use of MSCs in HSPC-based GE applications.

## Results

### Characterization of the hematopoietic supportive capacity of human MSCs

Human MSCs were isolated according to standard protocols^36^ from pediatric sibling donors undergoing BM harvest (median age: 12 years, range: 4–18 years) after parental informed consent. BM-MSCs were *ex vivo* expanded and characterized in terms of clonogenic capacity, proliferation, expression of MSC markers, and differentiation potential according to the minimal criteria defined by the International Society for Cellular Therapy^4^ (Figures S1A–S1F).

In detail, all the MSC samples used in this study were capable of forming colony-forming unit fibroblasts (CFU-Fs) (Figure S1A), acquired a spindle-like morphology (Figure S1B), and efficiently proliferated between passage 3 to 6 *in vitro* (Figure S1C). All samples expressed the canonical MSC surface markers (CD90, CD73, CD105) and lacked the expression of hematopoietic (CD45, CD34, CD14), endothelial (CD31), and HLA class II (HLA-DR) markers (Figure S1D). As expected, they differentiated *in vitro* into adipocytes, osteoblasts, and chondrocytes (Figures S1E and S1F).

Importantly, all *ex vivo* expanded MSC samples show a higher expression than human fibroblasts of several transcripts encoding for hematopoietic supportive factors known to sustain and regulate the fate of human HSPCs in the BM niche (Figure S1G), some of which were also detected in the MSC-conditioned medium (Figure S1H). MSCs also released several anti-inflammatory molecules, as shown in Figure S1I.

Based on these results, we set the MSC-based co-culture conditions to exploit the hematopoietic supportive activity of *ex vivo* expanded human MSCs. In detail, MSCs were expanded in their proper medium for 2 days and exposed to HSPC culture medium for 24 h to enrich it with MSC-derived hematopoietic supportive factors. Commercially available human UCB-CD34^+^ cells (hCD34^+^) were co-cultured on an MSC feeder for 72 h in the previously obtained MSC-conditioned HSPC culture medium supplemented with the proper early-acting cytokines (+cytokines)^31^ (Figure S2A). At the end of the co-culture, we determined the total number of hCD34^+^ cells and evaluated their phenotypic composition and clonogenic capacity (Figures S2B–S2E). To this aim, we applied a novel flow cytometer gating strategy to prospectively identify the most primitive HSPC subpopulation as CD45^+^, Lin^−^, CD34^high^, CD90^+^, CD45RA^−^ cells (Figure S3A).^37^^,^^38^^,^^39^^,^^40^^,^^41^^,^^42^ Human HSPCs proliferated in culture in the presence of cytokines, reaching a significantly higher number of cells (p = 0.0079) when co-cultured with MSCs (Figure S2B). Phenotypic analysis resulted in a significantly higher number of HSPCs with a primitive phenotype (p = 0.02) (Figures S2C and S2D) characterized by an increased clonogenic capacity (p = 0.02) (Figure S2E) when cultured in the presence of MSCs compared with controls.

Overall, these data confirm that BM-MSCs promoted survival and maintenance of HSPCs with a primitive phenotype, and this effect was also evident in stress conditions, such as in the absence of supplementation with early-acting cytokines (−cytokines) (Figures S2B–S2E).

### MSCs increase the number of GE-HSPCs available for HSCT

One of the main limitations to the clinical employment of HDR-edited HSPCs is the limited number of gene-corrected repopulating cells available due to the strong activation of a p53-mediated DDR elicited by the converging effect of nuclease-induced DNA DSBs and the cellular sensing of the adeno-associated viral vector serotype 6 (AAV6) used as source for DNA donor template delivery. As a consequence, exacerbated DDR constrains the proliferative and LT repopulating capacity of edited HSPCs.^35^

**Figure 1:**
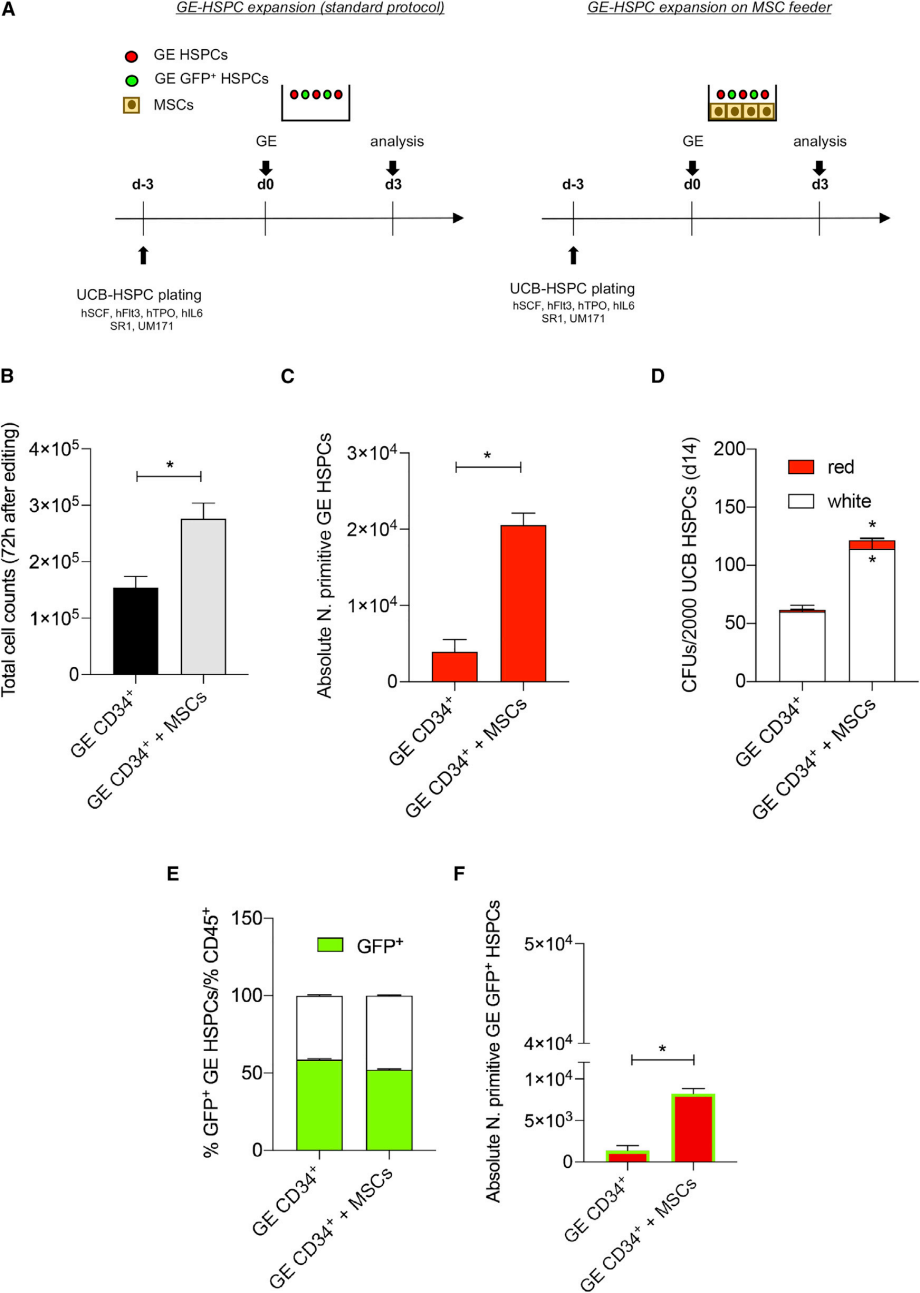
MSCs favored the expansion of GE-HSPCs (A) Schematic representation of the co-culture protocol used to support the expansion of GE-HSPCs. HSPCs were pre-stimulated for 3 days in HSPC GE medium (hSCF, hFLT3, hTPO, hIL-6, SR1, UM171) and recovered in HSPC medium conditioned from MSCs in the presence of MSCs for 72 h. GE-HSPCs recovered in HSPC medium on plastic according to our standard method were used as controls. (B) GE-HSPC total cell count after 72 h expansion. Gray bars: GE-HSPCs expanded on MSC feeder; black bars: GE-HSPCs expanded in plastic dishes. (C) Absolute number of GE-HSPCs with a primitive phenotype was determined after 72 h expansion. Each error bar shows means ± SEM (n = 4). (D) CFU assay at 7 days after plating GE-HSPCs into methylcelluose medium. (E) Percentage of GFP^+^ HSPCs 72 h after gene editing in the presence of MSCs (GE CD34^+^ + MSCs) and according to standard protocol (GE CD34^+^) (n = 4). (F) Absolute number of phenotypically primitive GFP^+^ GE-HSPCs after 72 h expansion. For all experiments, each error bar shows means ± SEM (n = 4). p values were determined by Mann-Whitney test (∗p ≤ 0.05).

We reasoned that the BM-MSC supporting feeder could counteract GE-induced DDR and cell-cycle arrest, thus favoring the expansion of GE-HSPCs. To this purpose, human CD34^+^ cells were gene edited with a CRISPR-Cas9 ribonucleoprotein (RNP) targeting the *AAVS1* safe harbor locus and transduced with a cognate AAV6 donor DNA suitable for HDR and encoding for GFP under the transcriptional control of the phosphoglycerate kinase (PGK) promoter,^35^^,^^43^ further referred as standard protocol. Cells treated for GE were expanded on MSCs for 72 h, as represented in Figure 1A and described in the materials and methods. Total count, phenotype, and absolute number of HSPCs were evaluated to assess the hematopoietic supportive capacity of MSCs on HSPCs treated for GE (Figures 1B–1F).

The number of GE-HSPCs co-cultured with MSCs was twice the number of control cells (p = 0.05) (Figure 1B). Importantly, MSCs favored the maintenance of the GE-HSPC subset with a primitive phenotype as shown by flow cytometry analysis (Figure S3B) and absolute counts of CD45^+^, Lin^−^, CD34^high^, CD90^+^, CD45RA^−^ GE-HSPCs (Figure 1C), which were significantly higher (p = 0.02) in the presence of MSCs. Moreover, GE-HSPCs formed a higher number of colonies (white colonies: p = 0.02; red colonies: p = 0.05), confirming an enrichment of highly clonogenic cells in GE-HSPCs co-cultured with MSCs (Figure 1D). Importantly, while we observed that MSCs did not alter the efficiency of DSB repair via HDR (Figure 1E), when dissecting the phenotype of GFP^+^ GE-HSPCs, we found a higher number of GFP^+^ GE-HSPCs with a primitive phenotype (CD45^+^, Lin^−^, CD34^high^, CD90^+^, CD45RA^−^) after MSC co-culture (p = 0.02) (Figure 1F).

Altogether, these results indicate that the *ex vivo* co-culture of HSPCs treated for GE with BM-MSCs represents a suitable strategy to expand GE-HSPCs while preserving the HSPC subsets with a primitive phenotype, including HSCs (CD90+, CD45RA−), multimyeloid progenitors, MMPs (CD90^−^, CD45RA^−^), and multilymphoid progenitors (MLPs; CD90^−^, CD45RA^+^) available for transplantation.

Despite the protective effects of MSCs, we noted that the 72 h culture of GE-HSCPs significantly reduced the frequency of CD34^+^ cells with a primitive phenotype compared with the 24 h recovery time, likely due to the culture-induced HPSC activation (Figure S3C). These results (Figures 1, S2, S3B, and S3C) correlate with the time of GE-HSPC transplantation: indeed, HSPCs are usually *ex vivo* cultured for 72 h prior to genetic engineering and infused 24 h after GE in pre-clinical mouse models of HSCT.

**Figure 2:**
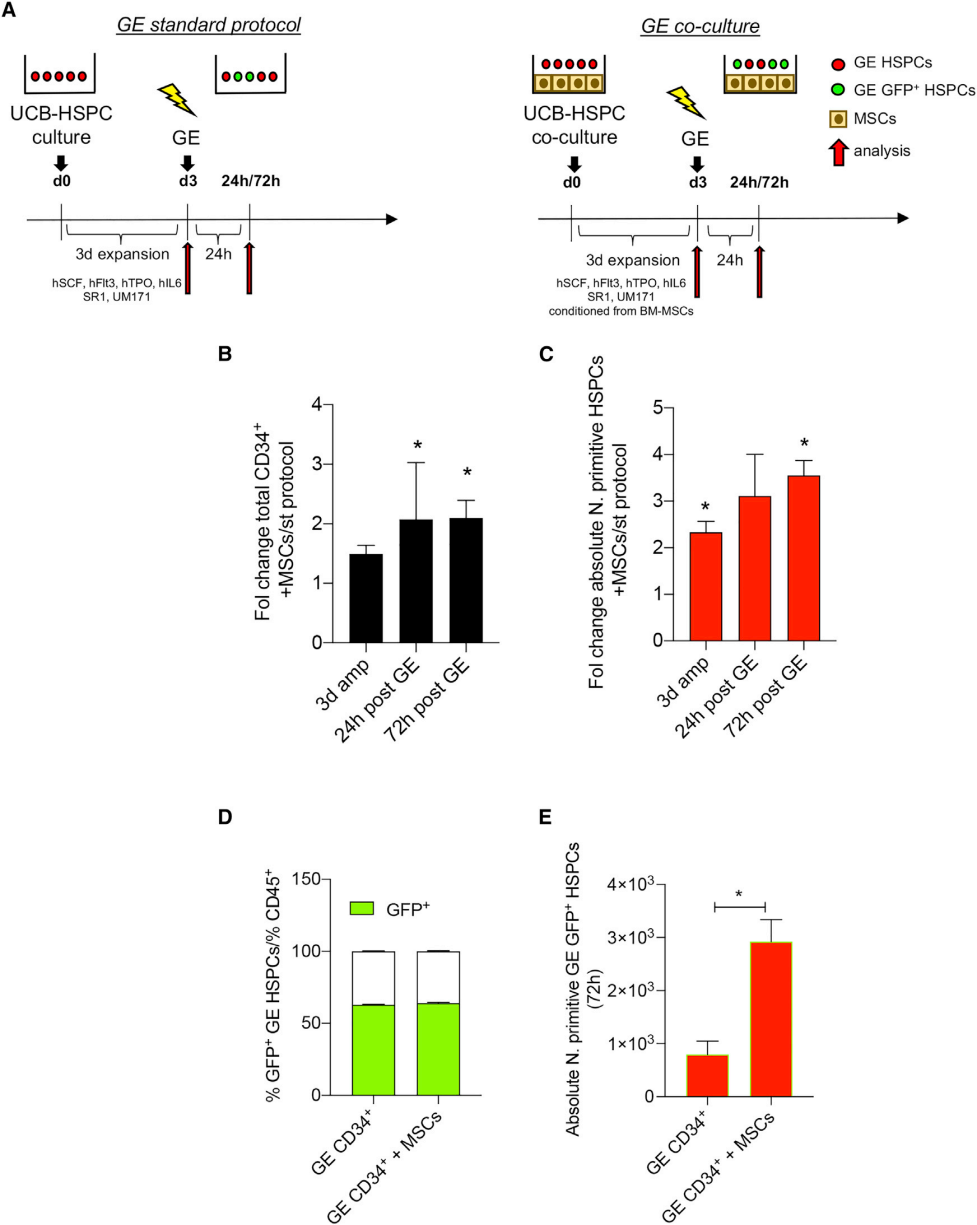
MSCs sustain the expansion and maintenance of GE-HSPCs when used as supportive feeder in standard protocol of gene editing (A) Schematic representation of the experimental plan used to support UCB-HSPCs undergoing gene editing. BM-MSCs were expanded for 72 h before co-culture with GE-HSPCs. HSPCs were pre-stimulated and recovered upon gene editing on BM-MSC feeder in GE medium 24 h conditioned from BM-MSCs. HSPCs gene edited according to our standard protocol were used as controls. (B and C) Fold change analysis of CD34+ total cell counts (B) and absolute number of HSPCs with a primitive phenotype (C) after 3 days of expansion before editing (3d amp) and after 24 and 72 h recovery after editing in the presence of MSCs compared with standard protocol. (D) Percentage of GFP^+^ GE-HSPCs cells after 72 h expansion on MSC feeder (GE CD34^+^ + MSCs) or in plastic dishes (GE CD34^+^). (E) Absolute number of HDR gene-edited (GFP^+^) GE-HSPCs after co-cultured with MSCs (GE CD34^+^ + MSCs) or expanded in plastic dishes (GE CD34^+^) according to standard protocol. For all the experiments, each error bar shows means ± SEM (n ≥ 3). p values were determined by Mann-Whitney test (∗p ≤ 0.05).

Based on this, we reasoned to exploit the hematopoietic supportive activity of MSCs in both the stimulation (3 day expansion before GE) and recovery phase post-GE of our GE protocol (Figure 2A).

We analyzed the total cell count, phenotype, and absolute number of HSPCs after 72 h of pre-stimulation, at 24 h recovery, and 72 h expansion post-editing. For all time points, we found a significantly higher (pre-stimulation: p = 0.05, 24 h recovery: p = 0.05; 72 h expansion: p = 0.03) number of CD34^+^ in the presence of MSCs compared with controls (Figures 2B and S3D). We confirmed that the co-culture with MSCs favored the expansion of cells treated for GE, and we also observed a diminished loss of Lin^−^, CD34^high^, CD90^+^, CD45RA^−^ HSPCs when CD34^+^ cells were pre-stimulated in co-culture with MSCs (Figures 2C, S3D, and S3E). Indeed, we found that MSCs prevented the reduction of phenotypically primitive HSPCs at 24 h post-editing and at longer time points, despite the additional effect of culture-induced HSPC differentiation (Figures 2C and S3F).

Importantly, we found that the efficiency of gene correction was not affected by the presence of MSCs in co-culture with HSPCs (Figure 2D). We also observed a significantly higher number of HSPCs gene edited by HDR (GFP^+^) with a primitive phenotype in the presence of MSCs (Figure 2E). The combined use of MSCs to expand HSPCs before GE and to recover HSPCs after GE procedures confirmed that the presence of MSCs enriched the total number of GFP^+^ GE-HSPCs with a primitive phenotype (Figure 2E). We also showed a similar percentage of HDR and NHEJ at 72 h post-editing in cells gene edited in the presence of MSCs compared with our standard controls (Figure S3G).

Considering the fundamental role of the vascular niche in the control of HSPC homeostasis and the functional interaction of endothelial cells with MSCs,^2^ we aimed at further improving the hematopoietic support of our 2D co-culture system by using a mixed feeder of human umbilical vein endothelial cells (HUVECs) and MSCs. We observed that the total cell counts and the absolute number of phenotypically primitive HSPCs tended to increase in the presence of a mixed feeder (MSCs:HUVECs, 10:1) compared with standard protocol and the use of MSCs alone (Figures S3H and S3I). However, when we translated this approach to GE-HSPCs, HUVECs died in the presence of UM171 and SR1, which are important to support maintenance and expansion of GE HSPCs in culture (Figure S3J).

In conclusion, we exploited the use of MSCs alone to increase the number of gene-targeted cells available for transplantation and to preserve their primitive phenotype.

### HSPCs gene edited in the presence of MSCs showed a superior engraftment capacity

We next investigated whether the increased absolute number of HSPCs obtained using BM-MSCs as a feeder during the GE procedures correlated with an improved transplantation efficacy. To assess the clonal composition and dynamics of edited cells in the human xenograft, we performed GE in the same locus but using an AAV6 also embedding unique molecular barcodes to individually track HDR-edited cell clones by barcode analysis by sequencing (BAR-seq) technology.^44^^,^^45^

We transplanted the outgrown cells of starting matched doses (1 × 10^5^) of UCB-derived GE-HSPCs co-cultured with MSCs or standard GE-HSPCs, as control, into sublethally irradiated immunodeficient non-obese diabetic (NOD)-severe combined immunodeficiency (SCID)-IL2Rg^−/−^ (NSG) mice. Human cell engraftment and hematological reconstitution were evaluated in the peripheral blood (PB) of transplanted mice at different time points and in the BM at the end of the experiment (16 weeks) (Figure S4A).

**Figure 3:**
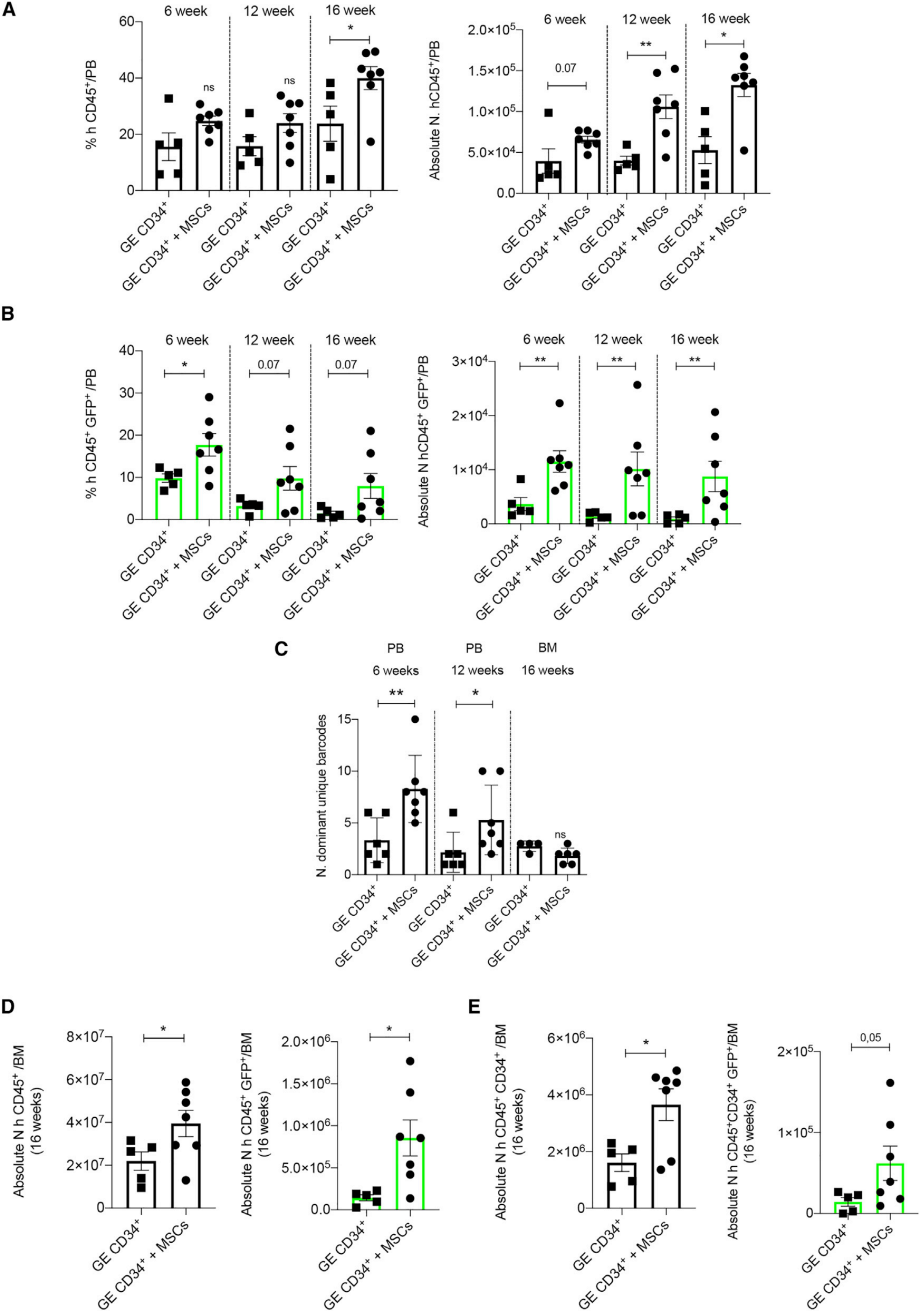
Improved HSCT outcome after transplantation of HSPCs gene edited in the presence of MSCs into a pre-clinical xenograft model (A) Flow cytometry analysis of human hematopoietic cell engraftment represented as percentage of hCD45^+^ cells on total live cells based on physical parameters in the peripheral blood (PB) of mice transplanted with GE-HSPCs co-cultured with BM-MSCs (rounded points) or according to standard protocol (squared points) at different time points after transplantation (left panel). The absolute number of hCD45^+^ cells was determined by flow cytometry adding known amount of Count Beads to the blood sample and using the counting equation according to the manufacturer protocol of absolute counting beads for flow cytometry (right panel). (B) Engraftment analysis of HDR gene-edited (GFP^+^) human CD45^+^ cells on total live cells based on physical parameters in the PB of transplanted mice at different time points after cell infusion (left panel). The absolute number of hCD45^+^ cells was determined by flow cytometry using absolute counting beads (right panel). (C) Number of unique dominant barcodes identified in the PB (6 and 12 week time points) and in the BM (16 week time point) of mice transplanted with GE-HSPCs + MSCs (GE CD34^+^ + MSCs) or standard GE-HSPCs (GE CD34^+^) at different time points after cell infusion. (D) Absolute number of human CD45^+^ cells (left panel) and human HDR gene-edited (GFP^+^) CD45^+^ cells (right panel) on total live cells based on physical parameters engrafted in the BM of transplanted mice at sacrifice. (E) Absolute number of human CD34^+^ (left panel) and HDR gene-edited (GFP^+^) CD34^+^ (right panel) cells within the human CD45^+^ engraftment on total live cells in the bone marrow at sacrifice (16 weeks). For all plots, individual data points represent an animal. p values were determined by Mann-Whitney test (∗p ≤ 0.05; ∗∗p ≤ 0.001).

We showed an increased human engraftment, which was statistically significant (p = 0.004) at 12 and 16 weeks, in the PB of mice transplanted with GE-HSPCs + MSCs (Figure 3A), indicating that *ex vivo* co-culture with MSCs favored durable human CD45^+^ cell engraftment. In these mice, we also found an increased number of HDR-edited (GFP^+^) cells, which was maintained over time post-transplantation (Figure 3B).

Moreover, BAR-seq analysis showed an increase in repopulating HDR-edited clones,^44^^,^^45^ as supported by the higher number of dominant unique barcodes retrieved from PB of the mice, at 6 and 12 weeks after transplantation of HSPCs gene edited in the presence of MSCs (Figures 3C and S5A). On the contrary, the number of gene-edited human CD45^+^ cells decreased overtime in the PB of mice transplanted with standard GE-HSPCs, and overall fewer clones participated in the hematological reconstitution, as expected by the kinetics of hematopoietic reconstitution and also as previously described (Figures 3C and S5A).^39^^,^^44^^,^^46^ Despite a trend for decreased engraftment of human GFP^+^ cells in the PB at a later time point (16 week), the number of mice with a detectable level of GFP^+^ (>0.1%) was higher in the GE-HSPC + MSC group (Figure 3B) compared with mice transplanted according to our standard procedures.

In addition, the absolute number of human B cells (CD19^+^), T lymphocytes (CD3^+^), and myeloid cells (CD13^+^CD33^+^) was significantly higher in the PB of mice transplanted with GE-HSPCs + MSCs (Figures S4B–S4D). At the endpoint (16 weeks), we observed a trend for a higher level of human engraftment (Figure 3D, left panel) and a significant increase in the absolute number of hCD45^+^CD34^+^ cells (Figure 3E, left panel) in the BM of mice transplanted with GE-HSPCs cultured in the presence of MSCs. In these mice, the absolute number of human cells gene edited by HDR (GFP^+^) engrafted in the BM was significantly higher (p = 0.02) (Figure 3D, right panel) and showed a similar clonal reconstitution capacity compared with standard GE procedures (Figures 3C and S5A).

In conclusion, our results suggest that MSCs favored the expansion *in vitro* of early hematopoietic progenitors contributing to the improved early-phase engraftment and graft clonality while preserving the HSC compartment (CD34^high^CD90^+^CD45RA^−^) (Figure S5A). This correlated with the higher number of transplanted cells when co-cultured with MSCs. In this specific case, we confirmed that MSCs favored HSPC expansion (fold change on starting matched dose: 2.28 + MSCs; 1.7 standard protocol), reduced post-GE detrimental cellular response (percentage of cell death: + MSCs: 10%; standard protocol: 22.5%), and reduced the loss of phenotypically primitive GE-HSPCs, resulting in a higher absolute number of transplanted primitive cells (+MSCs: 1,114; standard protocol: 280). We calculated the number of effectively transplanted cells per mouse and observed an averaged 1.7-fold change difference between GE-HSPCs + MSCs and GE-HSPCs (Figure S4E). We determined the ratio between the absolute counts of hCD45^+^ cells in the PB and the number of effectively transplanted cells in both the transplantation conditions, and we calculated the fold change enrichment, which reflects the higher number of outgrown cells of starting matched doses co-cultured with MSCs (6 weeks: 1; 12 weeks: 1.53; 16 weeks: 1.45) (Figure S4F). We also determined the absolute counts of CD45^+^CD34^+^GFP cells in the BM of transplanted mice (+MSCs: 62,058; standard protocol: 14,378), and we normalized for the average number of transplanted outgrowing cells. Based on this calculation, we found an enrichment of a 2.5-fold change of gene-edited cells in the BM of mice transplanted with cells treated for GE in the presence of MSCs (Figure S4G), which is higher than the difference of effectively transplanted cells (1.7-fold), suggesting a specific supportive function of MSCs in sustaining GE cells.

### MSCs mitigate DDR-induced cell-cycle arrest in GE-HSPCs

Recently, some of us showed that the robust activation of the p53-mediated DDR pathway limits the proliferation of GE-HSPCs and their LT repopulating capacity upon transplantation into immunocompromised mice.^35^

To further dissect the molecular mechanisms through which BM-MSCs promote the expansion and maintenance of HSPCs treated for GE, we analyzed the extent of DDR activation in GE-HSPCs co-cultured with MSCs at 24 and 72 h after GE (GE CD34^+^ + MSCs). HSPCs gene edited according to our standard procedures were analyzed as control (GE CD34^+^).

**Figure 4:**
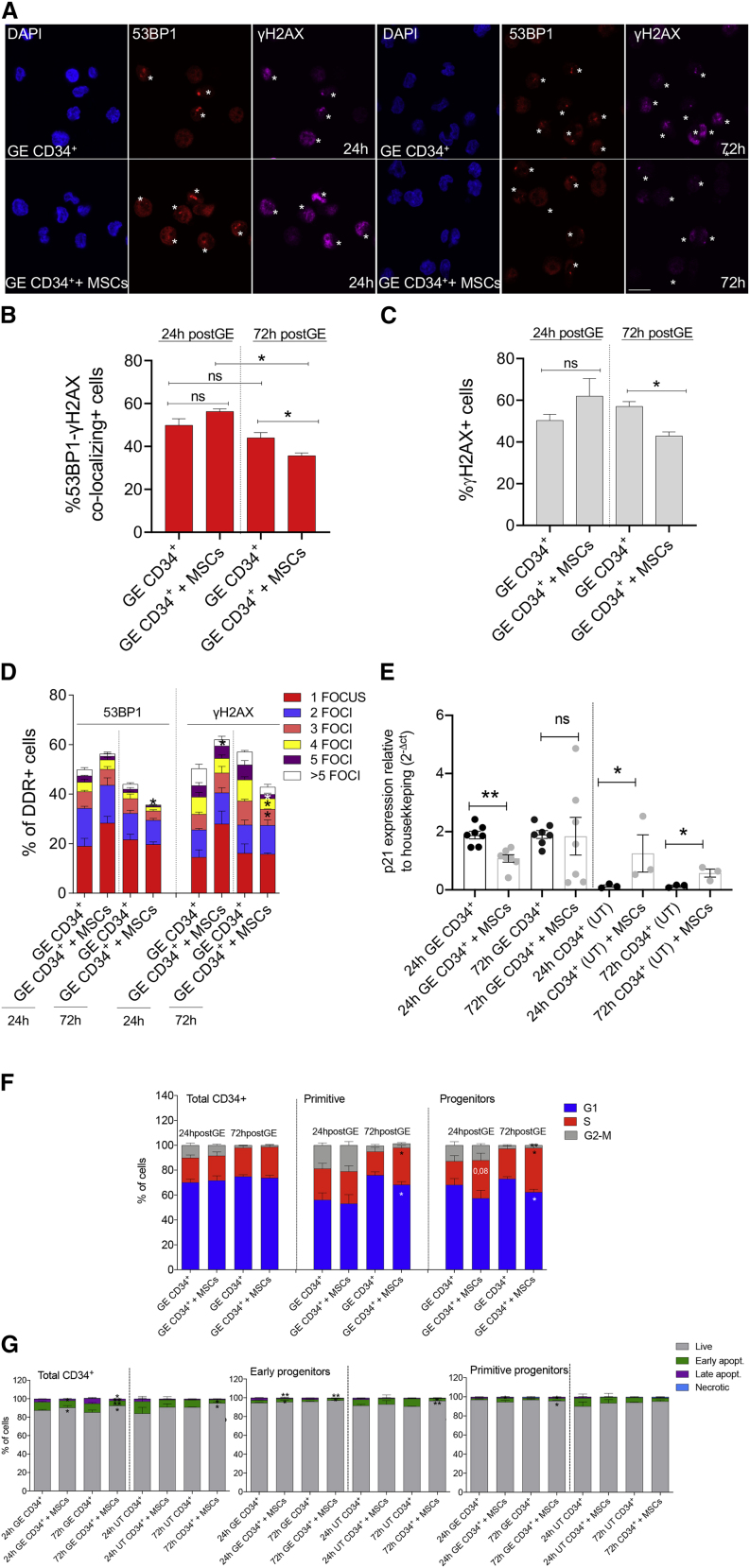
MSCs accelerate DDR foci resolution, preventing the cell-cycle delay and reducing the level of apoptosis in GE-HSPCs (A) Confocal images of 53BP1-positive foci (red), γH2AX-positive foci (purple), and DAPI-positive nuclei (blue) in HSPCs gene edited according to standard procedures (GE CD34^+^) or in the presence of MSCs (GE CD34^+^ + MSCs) at 24 and 72 h upon gene editing. Asterisks indicate positive foci. Scale bar represents 20 μm. (B and C) Quantification of 53BP1-γH2AX co-localizing foci (B) and all γH2AX (C) foci from (A); 24 h (53BP1): n = 4; 72 h (53BP1): n = 4; 24 h (γH2AX): n = 4; 72 h (γH2AX): n = 3. Each error bar shows means ± SEM. p values were determined by Mann-Whitney test (ns p > 0,05; ∗p < 0.05). (D) Quantification of 53BP1- and γH2AX-positive foci from (A)–(C). Positive cells were divided in subgroups according to the number of foci identified per cell (from 1 to more than 5). 24 h (53BP1): n = 4; 72 h (53BP1): n = 4; 24 h (γH2AX): n = 4; 72 h (γH2AX): n = 3. Each error bar shows means ± SEM. p values were determined by Mann-Whitney test for each subgroup (only significant comparisons were reported; ∗p ≤ 0.05). (E) qPCR expression analysis of *p21* in untreated (UT) and gene-edited (GE) HSPCs co-cultured with MSCs or in plastic dishes for 24 and 72 h. Each error bar shows means ± SEM (n ≥ 3). Gene expression was calculated as 2^−ΔCT^ relative to *GUSB* gene. (F) Percentage of GE-HSPCs (distinguished between primitive cells and progenitors) in the indicated cell-cycle phases measured at 24 (n = 7) and 72 h (n = 6) after editing. Significance was calculated for each time point comparing GE CD34^+^ + MSCs with GE CD34^+^. Each error bar shows means ± SEM. p values were determined by Mann-Whitney test for each cell-cycle phase (only significant comparisons were reported; ∗p ≤ 0.05). (G) Percentage of apoptotic cells within different GE-HSPC subsets (primitive cells and progenitors) at the indicated time points after gene editing. Live: Annexin V^−^, 7AAD^−^; early apoptotic: Annexin V^+^, 7AAD^−^; late apoptotic: Annexin V^+^, 7AAD^+^; necrotic: Annexin V^−^, 7AAD^+^. n = 3 for each time point analyzed.

We first measured the subnuclear accumulation of 53BP1 and γH2AX foci in GE-HSPCs,^35^ as surrogate markers of both nuclease-induced DSB (1–2 foci/nucleus) and culture-induced stress (>2 foci/nucleus) (Figure 4A). Twenty-four h after editing, we observed a higher number of 53BP1- and γH2AX-positive HSPCs compared with untreated (UT) samples and comparable levels of DDR foci when HSPCs were edited in the presence or absence of MSCs (Figures 4B and 4C). However, at later time points (72 h), we found a lower percentage of cells positive for 53BP1-γH2AX foci and a faster DDR resolution in GE-HSPCs co-cultured with MSCs. While the percentage of cells with one or two DDR foci per nucleus was similar in HSPCs gene edited in the presence or absence of BM-MSCs (Figure 4D), we found that the fraction of cells displaying more than 2 DDR foci, likely reflecting the DNA replication stress induced by their excessive proliferation in culture, was reduced in GE-HSPCs + MSCs, and this reduction was more evident in GE-HSPCs than in UT samples (Figure 4D).

Altogether, these data suggest that MSCs may favor the recovery of GE-HSPCs by both accelerating DDR foci resolution over time and protecting HSPCs from culture-induced stress or possibly by facilitating the growth of healthier cells over cells displaying a higher DDR burden.

We next measured the expression levels of *CDKN1A* (*p21*), a cell-cycle inhibitor downstream of the p53-DDR activation cascade, which was upregulated in GE-HSPCs compared with UT controls Figure 4E), in line with the induction of a stronger DDR in GE-HSPCs.^35^ The upregulation of *p21* was mitigated in GE-HSPCs after 24 h of co-culture with MSCs (Figure 4E), indicating that MSCs may protect GE-HSPCs from p53-mediated cell-cycle arrest and apoptosis. Of note, UT HSPCs co-cultured with MSCs displayed higher levels of *p21*, possibly reflecting a feedback mechanism to control MSC-induced excessive proliferation in control cells.^47^

We also analyzed the impact of GE procedures on cell-cycle distribution and observed a decreased percentage of primitive and progenitor GE-HSPCs arrested in the G1 phase with a concomitant increase in cells in the S phase of the cell cycle 72 h post-GE in the presence of a supportive layer of MSCs (Figure 4F). In the same condition, we also detected a decrease in the percentage of cells in the G2/M phase in the more committed progenitor fraction (Figure S6A). No differences in cell-cycle distribution were observed in UT samples co-cultured with MSCs (Figure S6B).

Moreover, the presence of BM-MSCs reduced the apoptotic rate in the GE-HSPC progenitor subsets and the percentage of pan nuclear-stained γH2AX-positive cells (a surrogate marker for apoptosis) while increasing the expression of two key pro-survival factors, *BCL2α* and *BCL2β*, 72 h after editing (Figures 4G and S6C–S6G). For some of these molecular readouts, we also report a beneficial anti-apoptotic effect of MSCs on UT cells (Figures S6C–S6G).

We further investigated the expression of inflammatory cytokines in GE-HSPCs, considering the capacity of MSCs to sense and regulate the inflammation state of surrounding cells.^48^ We found that the expression of CXC chemokine ligand 8 (*CXCL8*; hereafter named *IL-8*) and monocyte chemoattractant protein 1 (*MCP-1*; also known as *CCL2*), two pro-inflammatory cytokines activated in response to stress,^49^^,^^50^ were induced by the GE procedure compared with UT cells, and their levels were reduced in GE-HSPCs cultured in the presence of an MSC feeder (Figures S6H and S6I), whereas the expression of another pro-inflammatory molecule, tumor necrosis factor alpha (*TNF-α*), did not change in the presence of MSCs (Figure S6J). An early upregulation of *IL-8* was observed when UT cells were co-cultured with MSCs (Figure S6H).

To determine whether the capacity of MSCs to mediate the repair proficiency and attenuate the inflammatory programs in GE-HSPCs was maintained over time, we replated GE-HSPCs previously cultured with or without MSCs into a medium with a low concentration of selected cytokines (20 ng/mL hTPO; 100 ng/mL hSCF; 1 μM SR1; 50 nM UM171) and in the absence of MSCs. In these conditions, we found a significant downregulation of *p21* and *CCL2* expression, as well as a trend toward decreasing *IL8* in GE-HSPCs previously co-cultured with MSCs, compared with control. Consistent with previous observations, *TNF-α* expression was still maintained similar to standard protocol GE-HSPCs (Figures S6K and S6L).

This suggested that MSCs mitigated the inflammatory signaling cascade in HSPCs treated for GE, keeping DDR activation under control, possibly due to their capacity to activate an anti-inflammatory program in response to inflammatory signals.^48^

We next performed a Luminex Multiplex assay on the supernatant of GE-HSPCs co-cultured with MSCs for 72 h after GE to identify possible paracrine factors supporting GE-HSPCs (Figure S7). In particular, we focused on those factors that could eventually protect HSPCs from the detrimental effects of DDR activation upon GE, including cell-cycle arrest and the activation of an inflammatory or apoptotic cascade.

We found that MSC-conditioned medium in the absence of additional cytokines contained significantly higher levels of hematopoietic supportive factors, including IL-2, IL-6, SDF1α, and VEGFA, compared with standard medium^51^^,^^52^^,^^53^^,^^54^ (Figure S7A). We also observed an increase of FGF-2 and HGF, which could contribute to preventing the cell-cycle arrest in HSPCs after the GE procedure^55^^,^^56^ (Figure S7B). Furthermore, we reported increased levels of LIF, which is known to regulate the proliferation of primitive HSPCs^57^ (Figure S7B). Conversely, we showed a significant reduction of MIP-1a and SCF in the MSC-conditioned medium (Figure S7C).

We next performed RNA sequencing analysis to identify genes specifically modulated in MSCs compared with MSCs exposed to GE-CD34^+^ cells (GEO: GSE168834). Principal-component analysis (PCA) showed a strong separation of samples belonging to the two groups (Figure S8A), which was also reflected in the high number of differentially expressed genes (DEGs) (false discovery rate [FDR] < 0.01 and |logFC| > 2) between MSCs and MSCs co-cultured with HSPCs treated for GE. In particular, we found a total of 2,551 DEGs, of which 2,307 were upregulated and 244 were downregulated (Table S1). Gene set enrichment analysis (GSEA) against the Gene Ontology (GO)-Biological Processes (BP) database on genes ranked by log fold change (FC) values highlighted the upregulation of pathways involved in cytokine secretion, response to external signals, including inflammatory molecules, cell-cycle control, and DNA-repairing mechanisms in MSCs co-cultured with GE-HSPCs (Figures S8B–S8D).

In the attempt to identify MSC factors supporting GE-HSPCs, we further analyzed the cytokine gene profiling of MSCs co-cultured with GE-HSPCs compared with MSCs alone. In MSCs co-cultured with GE-HSPCs, we found an enriched expression of TNF and TNF superfamily cytokines (Figures S8D and S8E), known to facilitate HSPC functions^58^ and to enhance the anti-inflammatory activity of MSCs, increasing their survival,^59^ (Figure S8C) and the MSC production of IL-10 and other supportive factors.^60^

We also observed that MSCs massively expressed *IL-10* and *IL-18* after co-culture with GE-HSPCs (Figure S8E). The release of IL-10 could mediate the reduction of inflammatory cytokines in GE-HSPCs,^48^ whereas IL-18 was described to control HSC quiescence in a murine setting.^61^ We also found that MSCs express a higher level of *IL-1B* after co-culture with GE-HSPCs. The inflammatory milieu associated with the GE procedure could induce *IL-1B* expression in MSCs,^62^ acting as a positive loop to increase the anti-inflammatory and supporting properties of MSCs.

Altogether, our results suggested that MSCs might be capable of sensing the inflammatory environment caused by the activation of the DNA-damage-induced stress signals in GE-HSPCs, activating, as a consequence, a pro-survival and anti-inflammatory signaling program to sustain GE cells (Figures 4 and S6–S8; Table S2).

We concluded that BM-MSCs contributed to faster resolution of DNA damage foci, in addition to reducing the culture stress in HSPCs treated for GE through the secretion of supportive, anti-inflammatory, and pro-survival factors.

### MSC paracrine activity and cell-to-cell contact are required to support human HSPCs in several pre-clinical applications of GE technology

We further dissected the mechanisms of MSC function in support of GE-HSPCs by analyzing the phenotype, the absolute number of phenotypically primitive GE-HSPCs, and the expression of pro-apoptotic, inflammatory, and survival genes in HSPCs gene edited according to our standard protocol in the presence of MSCs (direct co-culture and transwell) and MSC-conditioned medium.

**Figure 5:**
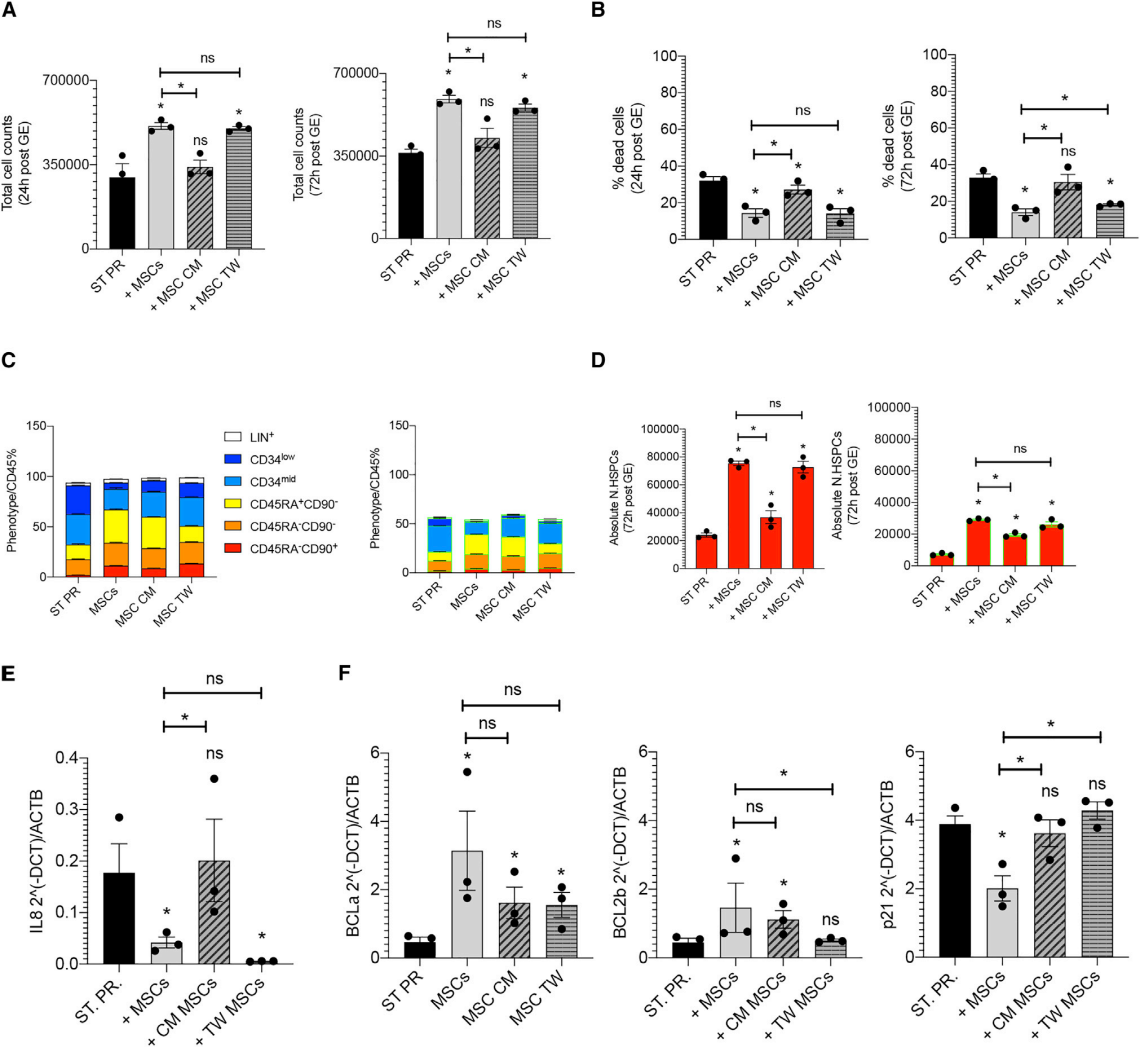
Comparison of the supportive capacity of MSC-conditioned medium, direct contact, and indirect transwell co-culture (A and B) Total cell count (A) and percentage of death (B) of GE-HSPCs recovered for 24 (left panel) and 72 h (right panel) after editing in the presence of MSC-conditioned medium and in direct (+MSCs) and indirect transwell co-culture (+MSC TW) with BM-MSCs. GE-HSPCs maintained in culture according to our standard protocol conditions were used as control (ST PR). (C and D) Phenotypic analysis of GE-HSPCs expanded in culture for 72 h after editing in the presence of MSC-conditioned medium, in direct (+MSCs) and indirect transwell co-culture (+MSC TW) with BM-MSCs, and according to our standard protocol (ST PR). The absolute number of phenotypically primitive GE-HSPCs and HSPCs GE by HDR (GFP^+^) is reported in (D). (E and F) Gene-expression analysis of inflammatory (E), pro-survival, and cell-cycle genes (F) in GE-HSPCs expanded in culture for 72 h after editing in the presence of MSC-conditioned medium, in direct (+MSCs) and indirect transwell co-culture (+MSC TW) with MSCs, and according to our standard protocol (ST PR). In our experimental setting, we could not distinguish properly the effects of direct contact from transwell MSC co-culture on GE-HSPCs since the majority of UCB CD34+ pre- and post-gene editing migrated toward the MSC feeder in the transwell bottom during the co-culture. Indeed, MSCs secrete SDF1α, a potent chemoattract for HSPCs.^53^ For all experiments, each error bar shows means ± SEM (n ≥ 3). p values were determined by Mann-Whitney test (∗p ≤ 0.05).

We observed that the MSC-conditioned medium alone supported the proliferation of GE-HSPCs (Figures 5A and 5B) and the maintenance of primitive GE-HSPCs less efficiently than our co-culture system (Figure 5C). The absolute count of phenotypically primitive GE-HSPCs and HSPCs gene edited by HDR (GFP^+^) was higher in the presence of MSC-conditioned medium than in the standard protocol conditions (Figure 5D), suggesting that MSCs are required to efficiently support GE-HSPCs. The level of *IL-8* expression decreased in HSPCs cultured in the MSC-conditioned medium and even more efficiently in the presence of MSCs (both direct contact and transwell co-culture) (Figure 5E).

The expression level of pro-survival genes *BCL2α* and *BCL2β* increased in GE-HSPCs exposed to the conditioned medium and co-cultured with MSCs. On the contrary, *p21* was significantly downregulated only in the presence of MSCs (Figure 5F).

We concluded that the direct contact with MSCs allows for a superior efficiency to protect HSPCs from the detrimental effects of GE, possibly because the cells represent a continuous source of hematopoietic supportive factors in addition to controlling HSPC homeostasis through direct cell contact in co-culture.

We also determined whether the use of MSCs could be clinically relevant to support HSPCs undergoing editing-based gene disruption (knockout [KO]). We used our experimental setting to co-culture KO-edited HSPCs on an MSC feeder, and we performed KO of the AAVS1 locus with a previously published high-specificity guide RNA.^35^ We analyzed the total cell count, the phenotype, and the absolute number of HSPCs with primitive phenotype 72 h after editing. We observed a higher number of KO-edited HSPCs and reduced mortality in the presence of MSCs (Figures S9A and S9B). MSCs also induced an expansion of hematopoietic progenitors while preserving the primitive HSPC subset (Figures S9C and S9D). The absolute number of KO-edited HSPCs with a primitive phenotype increased in the presence of MSCs. These results correlate with the upregulation of pro-survival genes and reduced expression of *BAX* in KO-edited HSPCs co-cultured with MSCs (Figure S9E). On the contrary, we did not observe a significant impact on the expression of cell-cycle arrest and inflammatory-related genes (Figures S9F and S9G), consistent with the observation that the activation of a DDR stress response and inflammatory program was more exacerbated in HSPCs undergoing HDR-mediated GE.^35^

### MSCs exert pro-survival and anti-inflammatory effects also on mobilized PB (mPB) GE CD34+ cells, resulting in an improved transplantation outcome

Considering that mPB CD34^+^ cells represent a clinically relevant source of HSPCs for transplantation, we applied our MSC-based co-culture system also to support mPB CD34^+^ cells undergoing GE.

**Figure 6:**
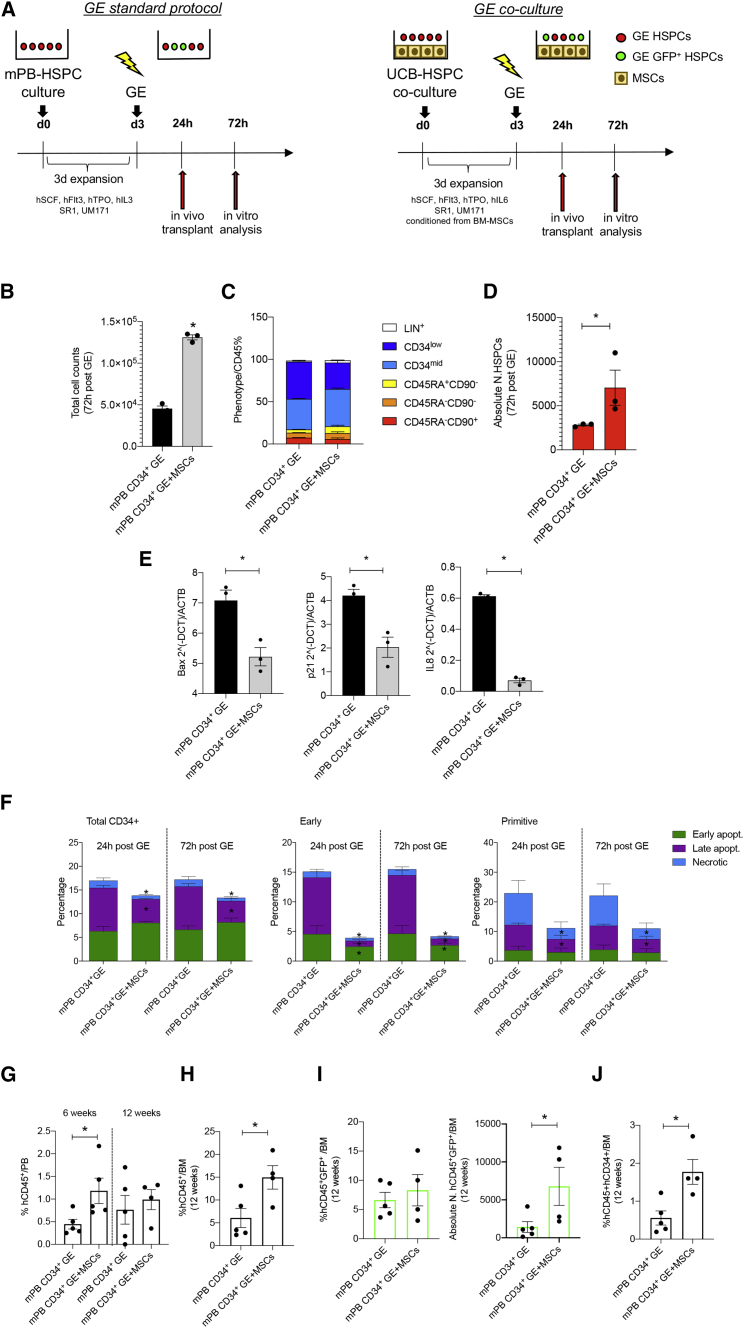
*In vitro* and *in vivo* analysis of the supporting activity of MSCs on GE mobilized PB (GE-mPB) HSPCs (A) Schematic representation of the *in vitro* and *in vivo* experiments using GE-mPB CD34^+^ cells. BM-MSCs were expanded for 72 h before co-culture with mPB HSPCs. Thereafter, mPB CD34^+^ cells were pre-stimulated in co-culture with BM-MSCs in GE medium conditioned from BM-MSCs and similarly recovered upon gene editing. GE-mPB HSPCs were transplanted into NSG mice after 24 h recovery, while GE-HPSCs were maintained in culture with BM-MSCs for 72 h for the *in vitro* analysis. mPB HSPCs GE according to our standard protocol were used as controls for the transplantation experiments at 24 h after gene editing and for the *in vitro* analysis after 72 h of culture. (B and C) Total cell counts (B) and phenotypic analysis (C) of GE-mPB recovered in culture 72 h after gene editing in the presence of MSCs (mPB CD34^+^ GE + MSCs). mPB HSPCs GE according to our standard protocol conditions were used as control (mPB CD34^+^ GE). The absolute number of phenotypically primitive GE-mPB HSPCs is reported in (D). *B*AX, *p21*, and *IL-8* expression analysis is reported in (E), whereas evaluation of apoptosis in GEmPB HSPCs recovered in culture 72 h after gene editing in the presence (mPB CD34^+^ GE + MSCs) or absence (mPB CD34^+^ GE) of an MSC feeder is reported in (F). (G) Percentage of human cell engraftment (%hCD45^+^) on total live cells in the PB of NSG mice transplanted with the outgrown of 0.5 × 10^6^ mPB CD34^+^ cells 24 h after GE in the presence of MSCs (mPB CD34^+^ GE + MSCs) or according to our standard protocol (mPB CD34^+^ GE) at 6 and 12 weeks after transplantation. (H) Analysis of human engraftment in the BM of transplanted mice at sacrifice (12 weeks). (I) Percentage (left panel) and absolute number (right panel) of HDR GE cells (GFP+) in the BM of transplanted mice at 12 weeks after transplantation. (J) Percentage of human cells positive for CD45 and CD34 engrafted on total live cells based on physical parameters in the BM after the transplantation of mPB CD34+ cells GE in the presence of MSCs (mPB CD34^+^ GE + MSCs) or according to our standard protocol (mPB CD34^+^ GE). For all plots, individual data points represent an animal. p values were determined by Mann-Whitney test (∗p ≤ 0.05; ∗∗p ≤ 0.001).

After 3 days of expansion in the presence of BM-MSCs, mPB CD34^+^ cells were gene edited and recovered for 72 h on an MSC feeder. mPB CD34^+^ cells gene edited according to our standard protocol were used as a control (Figure 6A). The total cell count of GE-mPB CD34^+^ cells significantly increased in co-culture with MSCs (Figure 6B). In the absence of MSCs, we observed a higher mortality rate after GE, and the number of cells retrieved after 72 h of culture was almost half of the initial number (1 × 10^5^) of cells undergoing GE, and we also observed an expansion of early progenitors when analyzing the phenotype of CD34^+^ cells after editing (Figure 6C). The absolute number of GE-mPB CD34^+^ cells with a primitive phenotype significantly increased in the presence of MSCs (Figure 6D). The percentage of HDR and non-homologous end joining (NHEJ) at 72 h post-editing in mPB cells gene edited in the presence of MSCs was similar to our standard controls (Figure S3G).

We reported a reduced expression of the pro-apoptotic gene *BAX*. *p21* was also downregulated in GE-mPB CD34^+^ cells co-cultured with MSCs. In addition, we also found a robust reduction of *IL-8* in GE-mPB CD34+ cells co-cultured with MSCs (Figure 6E). Consistently, we found that a significant reduction of late apoptotic cells was observed in GE-mPB CD34^+^ cells at 24 and 72 h after editing in the presence of MSCs. The pro-survival effects of MSCs were even more evident in early and primitive progenitors when dissecting the phenotype of co-cultured GE-mPB CD34^+^ cells (Figure 6F). We also found a robust reduction of *IL-8* in GE-mPB CD34^+^ cells co-cultured with MSCs (Figure 6E).

We then transplanted the culture equivalent of 0.5 × 10^6^ mPB CD34^+^ cells gene edited in the presence of MSCs or according to our standard protocol 24 h after *in vitro* recovery (Figure 6A). We followed the human cell engraftment in the PB, showing a higher engraftment of GEmPB CD34^+^ cells + MSCs at 6 weeks after transplantation (p = 0.019), which is in line with the improved early-phase engraftment observed in our previous experiments using UCB CD34^+^ cells (Figure 6G). At the endpoint, we observed a higher human engraftment (p = 0.03) and increased percentage of human CD34^+^ cells in the BM of mice transplanted with mPB CD34^+^ cells gene edited in the presence of MSCs (p = 0.01) (Figures 6H and 6J). In these samples, we also detected a higher number of human HDR-edited cells (CD45^+^GFP^+^) compared with our standard protocols (Figure 6I).

These data suggest that the pro-survival effects of MSCs on mPB CD34+ cells observed *in vitro* upon GE correlate with an improved transplantation efficacy.

## Discussion

In this study, we employed third-party human BM-MSCs as a supportive tool to sustain HSPCs undergoing GE. This strategy was based on pre-clinical and clinical data indicating that MSCs promote *ex vivo* expansion of unmanipulated HSPCs and facilitate their engraftment *in vivo*, thus improving the hematopoietic recovery of transplanted patients.^14^^,^^15^^,^^16^^,^^63^ These data prompted us to exploit the hematopoietic supportive capacity of MSCs in the context of HSPC GE. In this setting, extensive *ex vivo* manipulation impacts on self-renewal and differentiation potential of HDR gene-edited autologous HSPCs, thus altering their LT repopulating capacity.^35^ The activation of a strong DDR in cultured GE-HSPCs limits the clinical translation of GE technologies, potentially leading to a low dose of HDR-edited cells available for transplantation and accounting for delayed engraftment, oligoclonal hematopoietic reconstitution, and increased risk of graft failure in patients.

Our results demonstrate the ability of MSCs to favor the *ex vivo* expansion of GE-HSPCs and attenuate the loss of the host-repopulating HSPC subset, this translating into an improved transplantation outcome. While the *ex vivo* expansion effect driven by MSCs has been previously reported in unmanipulated HSPCs co-cultured with MSCs,^15^^,^^16^ no detailed analysis has been performed so far on the impact of MSC co-culture on the phenotype and functionality of HSPCs upon GE procedure. To this aim, we developed a novel strategy to dissect the phenotypic composition of *ex vivo* cultured HSPCs. We observed that MSCs were capable of preserving the phenotypically primitive and LT repopulating HSPC subset upon GE when activation both by *ex vivo* expansion and the GE procedure occur. Indeed, our co-culture system allowed us to significantly increase the total number of CD34^+^ cells and the number of phenotypically primitive Lin^−^, CD34^high^, CD90^+^, CD45RA^−^ cells available for transplantation (Figure 2), resulting in improved engraftment of human CD45^+^ and faster hematological reconstitution when HSPCs, gene edited in the presence of MSCs, were transplanted into NSG mice. In particular, the ability of MSCs to favor the proliferation of early progenitors while preserving the phenotypically primitive HSPC subset allowed a faster and more polyclonal early-phase reconstitution, without affecting the clonogenic potential of LT repopulating cells, as also demonstrated by the BAR-seq analysis, which indicates an increase in repopulating HDR-edited clones during the early-phase reconstitution. Indeed, an enrichment of gene-edited cells was found at sacrifice in the BM of mice transplanted with CD34^+^ cells treated for GE in the presence of MSCs (Figure S4G), indicating a specific supportive effect of MSCs on GE cells.

Limited information is available on the mechanisms by which MSCs support HSPC functions. Our data indicate that MSCs may act on DDR-dependent cell-cycle dynamics and mitigate the apoptotic and inflammatory cascade occurring in GE-HSPCs. Together with a p53-mediated DDR activation, inflammatory gene categories were reported by some of us to be enriched in GE-HSPCs.^35^ Even if basal levels of key pro-inflammatory cytokines, such as TNF-α, are reported to support HSC survival and regeneration during inflammation,^64^ several studies have highlighted the detrimental effects of chronic inflammatory programs in HSPCs,^50^^,^^65^^,^^66^ which may alter their fitness, induce their premature differentiation, reduce the efficacy of HSCT, and even predispose to acute myeloid leukemia (AML) or myelodysplastic syndrome (MDS) development.^67^ Given the DDR-dependent proliferative defect and the concomitant activation of an inflammatory cascade in GE-HSPCs, we reasoned that the use of MSCs could represent a valid approach to simultaneously counteract both mechanisms. Importantly, we observed that in response to GE, MSCs favored a faster resolution of DNA damage foci in HSPCs, reduced *p21* induction, and prevented the cell-cycle arrest observed upon standard GE procedures without affecting cell-cycle progression of UT cells (Figures 4 and S6).

Moreover, we also reported that MSCs reduced the level of apoptosis in all GE-HSPC subsets by inducing the expression of pro-survival genes in GE-HSPCs (Figure S3), in line with previous works showing MSC pro-survival ability in injured cells.^68^

In addition, HSPCs gene edited in the presence of MSCs displayed a significant reduction of the culture-induced proliferative stress, as shown by the reduced accumulation of γH2AX foci at early and late time points after editing. We also reported a lower percentage of 53BP1-γH2AX foci-positive cells in HSPCs treated for GE in the presence of MSCs at later time points. This evidence is consistent with the reported role of MSCs in mitigating DNA damage or replication stress in *in vitro* expanded murine HSCs.^69^ Despite the reduced presence of nuclear 53BP1, the efficiency of on-target repair by HDR editing in HSPCs co-cultured with MSCs was similar to standard protocol GE-HSPCs, as previously reported.^44^

Moreover, in support of this finding, we have shown the enrichment of several supportive factors in the medium collected from GE-HSPCs co-cultured with MSCs (Figure S4), suggesting that MSC paracrine activity plays a fundamental role in the control of proliferation, DNA repair, apoptosis, culture-induced stress, and maintenance of GE-HSPC stemness.

With respect to the activation of an inflammatory cascade reported in HSPCs treated for GE, MSCs have been reported to display a unique capacity to sense environmental signaling and activate a specific paracrine program to sustain neighboring cells and reduce the level of inflammation.^48^ While the anti-inflammatory capacity of MSCs has been already described in several disease animal models and in patients,^70^^,^^71^^,^^72^^,^^73^ in our work, we point to a new role of MSCs in the control of the inflammatory cascade occurring during HSPC-GE procedures, possibly through the release of anti-inflammatory molecules.^48^ Indeed, we reported the transcriptional downregulation of *IL-8* and *CCL2,* two key inflammatory factors related to HSPC dysfunction and reduced *ex vivo* expansion,^74^ when GE-HSPCs are cultured in the presence of MSCs compared with controls (Figures S6I and S6J).

In accordance with our data, it has been described that inflammatory cytokines prime MSCs toward an anti-inflammatory and pro-survival phenotype^75^ that may reduce the inflammation of HSPCs after GE, favoring the maintenance of the HSPC primitive subset. Similarly, in response to the release of damage-associated molecules, MSCs produce indoleamine dioxygenase (IDO), an enzyme with a wide range of anti-inflammatory properties.^76^ Moreover, IDO is also responsible for enhancing MSCs anti-inflammatory properties by reducing IL-8 production.^77^

In conclusion, our findings suggest possible molecular mechanisms responsible for the MSC-based proliferative and functional support of GE-HSPCs. In line with these findings, GSEA against the GO-BP database on RNA sequencing (RNA-seq) data of MSCs showed the activation of regulatory pathways involved in the control of inflammation and the cell cycle, in addition to an enriched set of genes involved in cytokine production and release, in MSCs co-cultured with GE-HSPCs (Figures 5B–5D; Table S2). Interestingly, we found several cytokines in the MSC-conditioned medium upon co-culture with GE-HSPCs that were enriched in our GSEA dataset, confirming the supportive paracrine activity of MSCs (Table S1; Figure S4).

Moreover, our results indicate that the MSC supportive activity protecting HSPCs from the detrimental effects of GE is superior when GE-HSPCs are directly co-cultured with MSCs (Figure 6), highlighting the dual role of MSCs in the control of HSPC homeostasis by employing both cell-to-cell contact and paracrine mechanisms.^78^^,^^79^ In the co-culture setting, MSCs represent a continuous source of hematopoietic supportive factors, in addition to controlling HSPC homeostasis through direct cell-to-cell contact. In support of this observation, LT repopulating HSPCs are associated with osteoprogenitors in the BM niche,^2^ and they show a more primitive phenotype if in contact with MSCs *in vitro*.^80^

Collectively, our data define an MSC-based co-culture system to sustain GE-HSPCs with the ultimate goal of preserving their functions in HSCT. Altogether, our results demonstrate that MSCs are capable of efficiently increasing the number of HDR gene-edited HSPCs, mitigating the hurdle concerning the low cell dose available for transplantation. The hematopoietic supportive activity of MSCs favored not only HSPC expansion but also the maintenance of phenotypically primitive HSPCs in culture, in addition to protecting HSPCs from cell-cycle delay and excessive activation, which are associated with GE procedures and exacerbated by *ex vivo* culture, leading to improved HSPC engraftment and transplantation outcomes. The MSC-driven effect on the early phase of hematological reconstitution was also supported by the increased number of GE-HSPC clones detected through the barcoded vector and demonstrates for the first time the ability of MSCs to sustain polyclonal reconstitution after HSCT.

Similar results were obtained *in vitro* when MSCs were co-cultured with GE-mPB CD34^+^ cells, highlighting the possibility of improving the outcome of GE also when HSPCs derived from PB are employed, these representing a very relevant source for clinical application. In these experiments, we also observed a positive correlation between the number of GE-HSPCs with a primitive phenotype in culture with BM-MSCs and the level of human engraftment, confirming that the increased dose of HSPCs mediated by BM-MSCs is associated with an increased level of engraftment of GE cells in humanized model of transplantation.

Finally, we showed that MSC co-culture could be useful to also support HSPCs undergoing editing-based gene disruption.

A prompt and sustained hematological reconstitution represents a desirable goal for patients undergoing HSCT who are at risk of graft failure and of developing life-threatening infections, especially in the early post-transplant phase. Our data provide a proof of principle that in the setting of GE, where extensive *ex vivo* manipulation may impact on self-renewal and differentiation potential of HSPCs, MSC-based approaches could be employed to counteract GE-related detrimental effects, thus optimizing transplantation outcome.

The clinical implementation of such MSC-based approaches would require their testing within experimental trials given that technologies for large-scale and clinical-grade expansion of MSCs have been extensively optimized and employed in the clinical setting. MSC co-culture could be employed alone or in combination with other emerging strategies to increase HDR efficiency, such as transient p53 inhibition and adenovirus 5 E4orf6/7 protein expression.^27^

Since MSC-derived secretome is emerging as a medicinal product for the treatment of several inflammatory conditions,^81^ this solution may be practically advantageous also in the setting of GE-HSPCs and might be employed in combination with the GE enhancers to preserve HSPCs and improve the GE-HSPC transplantation outcome.

## Materials and methods

### Isolation of human BM-derived MSCs

MSCs were isolated from residual BM aspirates of healthy donors (median age: 12 years; range: 4–18 years), who donated BM for transplantation at the San Raffaele Scientific Institute, after obtaining informed consent according to TIGET09 research protocol. TIGET09 is a protocol for the collection of biological material from healthy volunteers and patients with rare genetic diseases. It was approved by the Ethics Committee of San Raffaele Hospital on June 7^th^, 2017. MSCs were isolated according to a previously published protocol.^36^

### Expansion of human UCB-HSPCs on MSC feeder

MSCs were plated at a density of 10,000 cells/cm^2^ and expanded for 3 days in proper MSC medium. Twenty-four h before starting the co-culture experiment, MSC medium was replenished with HSPC medium with or without proper pre-stimulating factors for conditioning. Commercial UCB CD34^+^ HSPCs (Lonza, catalog 2C-101) were thawed at a density of 5 × 10^5^/mL and expanded in MSC-conditioned HSPC medium. As control, UCB CD34^+^ HSPCs were thawed and expanded in HSPC medium with or without the addition of proper stimulating cytokines (100 ng/μL hSCF, 100 ng/μL hFLT3, 20 ng/μL hTPO, 20 ng/ μL hIL-6). In the case of GE, SR1 (1 μM) and UM171 (50 nM) were added to the cytokine mix. After 72 h of culture, UCB CD34^+^ HSPCs were collected by gentle pipetting and analyzed in terms of total cell count, phenotype, and clonogenic capacity.

### LT expansion of GE-HSPCs on MSC feeder

MSC feeder was prepared as described above including medium conditioning. Commercial UCB CD34^+^ HSPCs (Lonza, catalog 2C-101) were edited following standard procedures^31^ and plated at a density of 5 × 10^5^ cells/mL on an MSC feeder in HSPC medium conditioned by MSCs for 72 h expansion. GE-HSPCs expanded for 72 h on plastic in the absence of MSCs were used as controls.

### Expansion and recovery of human HSPCs undergoing GE on MSC feeder

MSC feeder was prepared as described above including medium conditioning. Commercial UCB CD34^+^ HSPCs (Lonza, catalog 2C-101) undergoing GE were thawed at a density of 5 × 10^5^/mL on an MSC feeder and expanded for 3 days on an MSC feeder in HSPC medium conditioned by MSCs. After editing, GE HSPCs were maintained in culture for 24 h or expanded for 72 h on an MSC feeder at a density of 5 × 10^5^/mL in HSPC medium conditioned by MSCs. Human HSPCs edited according to standard protocol without the support of an MSC feeder were used as controls. Commercial mPB CD34^+^ cells (Stem Cell, catalog 70060) were thawed and expanded in HSPC medium (300 ng/μL hSCF, 300 ng/μL hFLT3, 100 ng/μL hTPO, 60 ng/μL hIL-3) with the addition of SR1 (1 μM) and UM171 (50 nM) in the presence or absence of MSCs. Upon GE, GE-HSPCs were recovered in culture on an MSC feeder using HSPC medium conditioned from MSCs or on plastic using HSPC medium.

### Flow cytometry

The capacity of MSCs to preserve HSPCs in culture was evaluated by flow cytometry analysis using the BD LSRFortessa. HSPCs were collected and washed with PBS + 2% FBS. 1 × 10^5^ cells were incubated with the proper antibody mix for 10 min at room temperature (RT) in the dark. The following antibodies were used: CD16 PE (BD Biosciences, 332779); CD14 PE (BioLegend, 301806); CD3 PE (BD Biosciences, 345765); CD15 PE (BioLegend, 301906); CD56 PE (BD Biosciences, 345812); CD19 PE (BD Biosciences, 345789); CD34 BV421 (BioLegend, 343610); CD45RA APC-H7 (BioLegend, 304128); CD45 BUV395 (BD Biosciences, 563792); and CD90 APC (BD Biosciences, 559869) for 10 min at RT in the dark. After washing with PBS + 2% FBS, cells were centrifuged for 5 min at 1,500 RPM and resuspended into 100 μL PBS + 2% FBS. Unstained cells were used as a negative control. All flow cytometry assays were standardized using SPHERO Rainbow Calibration Particles (8 peaks) (Spherotech, RCP305A). The primitive HSPC cell subset was phenotypically identified as CD45^+^, Lin^−^, CD34^high^, CD45RA^−^, CD90^+^ cells. Human cell engraftment and hematological reconstitution were determined on PB (50 μL) and BM samples (100 μL) of transplanted mice by flow cytometry. Red blood cells were lysed using ammonium-chloride-potassium (ACK) lysing solution for 15 min at RT, after the addition of precision counting beads (Biolegend, 424902). After washing twice with PBS + 2% FBS, cells were stained with the following antibody mix: CD45 APC (Biolegend, 304037); CD3 PE (BD Biosciences, 345765); CD19 PE Cy7 (BD Biosciences, 302216); CD33 VioBlue (Biolegend, 130-099-485); and CD13 PerCP Cy5.5 (BD Biosciences, 561361). BM samples were processed using the same protocol and stained with the following antibody mix: CD34 PB (Biolegend, 343511) PB and CD45 APC (Biolegend, 304037). Samples were run on BD FACSCanto II cytometer (BD Biosciences). At least 10,000 were recorded. Analysis of all fold cytometry results was performed using FlowJo software (Tree Star).

### Cell-cycle phase analysis by EdU/Hoechst staining

0.5–1 × 10^5^ cells were treated with 2 μM EdU for 4 h in culture (Thermo Fisher Scientific, C10636), washed with PBS + 1% BSA, and stained with the following antibodies mix for 15 min at 4°C: CD34 PE (Miltenyi Biotec 130-081-00), CD90 APC (BD Biosciences, 559869), and CD133/1 PECy7 (Miltenyi Biotec, 130-101-652). Cells were then fixed with 100 μL of Click-iT fixative for 15 min at RT upon cell-surface staining to distinguish the different HSPC subsets. After washing with PBS + 1% BSA, cells were permeabilized with 100 μL 1X Click-iT saponin for 15 min. Detection of EdU-DNA was performed by incubating cells with 500 μL Click-iT Plus reaction cocktail for 30 min at RT protected from light. Cells were subsequently washed with PBS + 1% BSA before overnight DNA staining with Hoechst at RT protected from light. Fluorescence was measured by flow cytometry on FACSymphony A5 SORP (BD Biosciences). Analysis of flow cytometry results was performed using FlowJo software.

### Apoptosis assay

Cell apoptosis was evaluated using Pacific Blue Annexin V (Biolegend, 640918) and 7-AAD Viability Staining Solution (BioLegend, 420403). In detail, GE-HSPCs were stained with the following antibodies mix for 15 min at 4°C: CD34 PE (Miltenyi Biotec 130-081-00), CD90 APC (BD Biosciences, 559869), and CD133/1 PECy7 (Miltenyi Biotec, 130-101-652). Cells were then washed with diluted 1:10 10X Annexin V Binding Buffer (BD Pharmingen, 556454) upon surface marker staining and stained with Pacific Blue Annexin V (Biolegend, 640918) and 7-AAD Viability Staining Solution (BioLegend, 420403) for 15 min at RT in the dark. After staining, cells were washed in 1X Annexin V Binding Buffer and acquired in 10 min. All samples were run on BD FACSCanto II cytometer (BD Biosciences). At least 10,000 were recorded. Analysis of flow cytometry results was performed using FlowJo software.

### Immunofluorescence analysis

Multitest slides (MP Biomedicals, 096041505) were coated with Poly-L-lysine solution (Sigma-Aldrich, P8920-500ML). After two washes with PBS solution, 0.3–0.5 × 10^5^ cells were seeded on covers for 20′ and fixed with 4% PFA (Santa Cruz Biotechnology, sc-281692) for 20′. Cells were then permeabilized with 0.1% Triton X-100. After blocking with 0.5% BSA and 0.2% fish gelatin in PBS, cells were stained with the indicated primary antibodies (53BP1 antibody, Bethyl Laboratories; anti-phospho histone H2A.X (Ser139) antibody, clone JBW301, Merck). Cells were than washed with PBS and incubated with Alexa 568- and 647-labeled secondary antibodies (Invitrogen). Nuclear DNA was stained with DAPI at 0.2 μg/mL concentration (Sigma-Aldrich, D9542), and covers were mounted with Aqua-Poly/Mount solution (TebuBio, 18606-20) on glass slides (Bio-Optica). Fluorescent images were acquired using Leica SP5 confocal microscopes. Quantification of DDR foci in immunofluorescence images was conducted using Cell Profiler.

### Mice

NSG mice were purchased from The Jackson Laboratory and maintained in specific-pathogen-free (SPF) conditions. The procedures involving animals were designed and performed with the approval of the Animal Care and Use Committee of the San Raffaele Hospital (IACUC #1039 and #1068) and communicated to the Ministry of Health and local authorities according to Italian law.

### GE-HSPC xenotransplantation experiments

Seven-week-old NSG mice were sublethally irradiated (150–180 Gy) and intravenously transplanted. 1 × 10^5^ HSPCs (t0 culture equivalent) were gently collected by pipetting and injected 24 h after GE according to standard procedures or in the presence of MSCs. In the case of GE mPB CD34^+^ cell transplantation, the outgrown cells of the 0.5 × 10^6^ dose were transplanted as described above. In both mouse models, human cell engraftment and the presence of HDR gene-edited cells were monitored by serial collection of PB from the mouse tail, and, at the end of the experiment (16 weeks after transplantation), BM was collected for endpoint analyses.

### Data availability

All relevant data are included in the manuscript. Sequencing data are deposited in the Gene Expression Omnibus with the access code GSE206904: RNA-seq (GSE168834) and BAR-Seq (GSE206900).

## Supplemental information

Document S1. Figures S1–S9 and supplemental methods

Table S1. List of DEGs in MSCs exposed to GE-HSPCs compared with MSCs

Table S2. List of the significantly enriched GO terms derived from the GSEA of the pre-ranked gene list resulting from the comparison between MSCs and MSCs co-cultured with GE-HSPCs

Document S2. Article plus supplemental information
