# Mesenchymal stromal cells improve the transplantation outcome of CRISPR-Cas9 gene-edited human HSPCs

**DOI:** 10.1016/j.ymthe.2022.09.005

**Published:** 2022-09-11

**Authors:** Stefania Crippa, Anastasia Conti, Valentina Vavassori, Samuele Ferrari, Stefano Beretta, Silvia Rivis, Roberto Bosotti, Serena Scala, Stefania Pirroni, Raisa Jofra-Hernandez, Ludovica Santi, Luca Basso-Ricci, Ivan Merelli, Pietro Genovese, Alessandro Aiuti, Luigi Naldini, Raffaella Di Micco, Maria Ester Bernardo

## Main text

(Molecular Therapy *31*, https://doi.org/10.1016/j.ymthe.2022.08.011; January 2023)

In the originally published version of this article, all sequencing data was deposited but not correctly reported in the data availability section. The sentence “RNA-seq data have been deposited in the Gene Expression Omnibus with the following access code: GEO: GSE168834.” has been changed to read: “Sequencing data are deposited in the Gene Expression Omnibus with the access code GSE206904: RNA-seq (GSE168834) and BAR-Seq (GSE206900).” The authors apologize for the inconvenience, and this error has been corrected online.

