# Supplementary material for: Mesenchymal stromal cells improve the transplantation outcome of CRISPR-Cas9 gene-edited human HSPCs
Source: Mol Ther. 2022 Aug 17;31(1):230–48. doi: 10.1016/j.ymthe.2022.08.011 (PMC9840125; doi:10.1016/j.ymthe.2022.08.011)
Supplement: Document S1. Figures S1–S9 and supplemental methods [file mmc1.pdf]

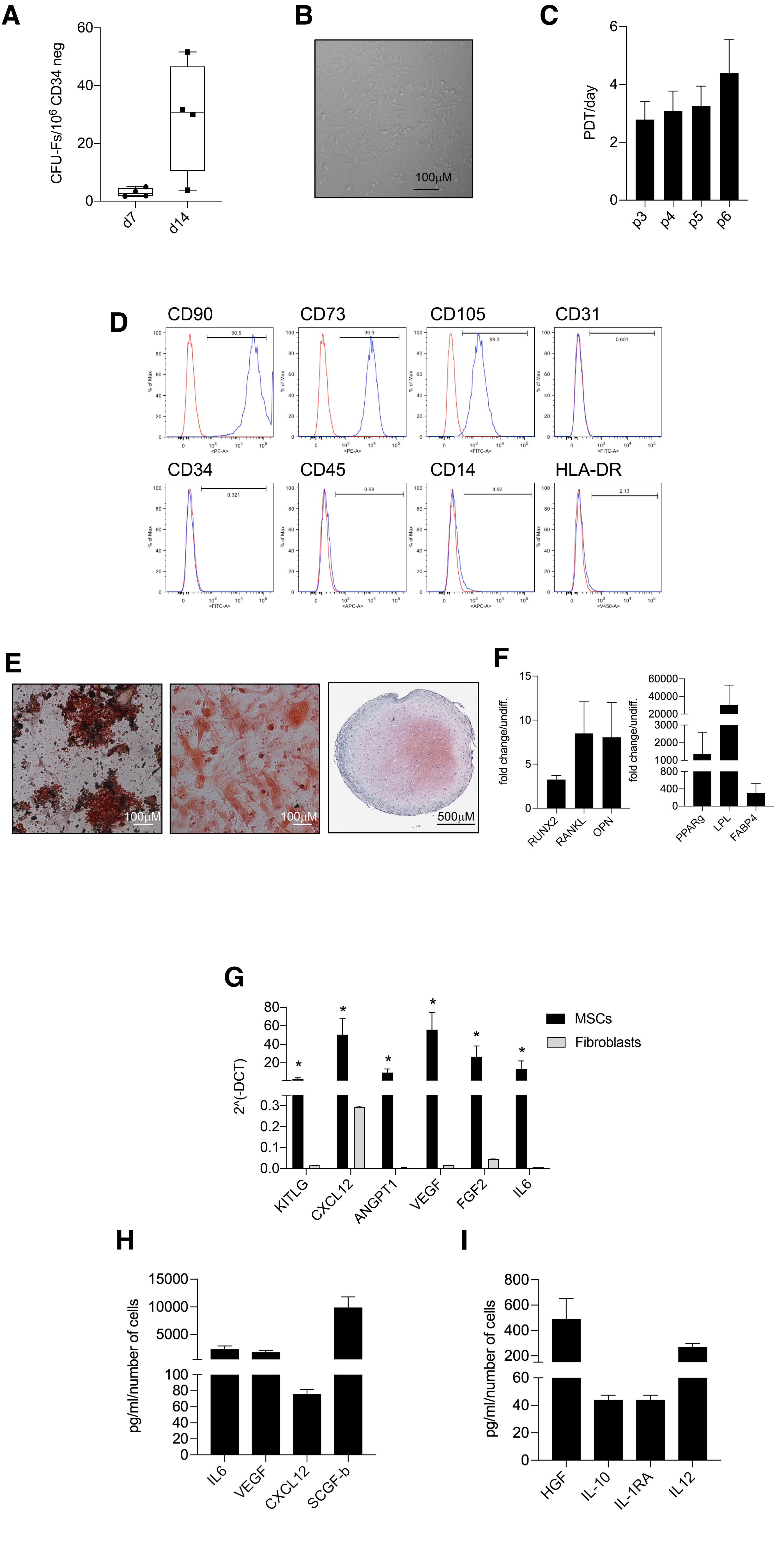
A
CFU-Fs/10^6 CD34 neg
d7
d14
B
100µM
C
PDT/day
p3
p4
p5
p6
D
CD90
CD73
CD105
CD31
CD34
CD45
CD14
HLA-DR
E
100µM
100µM
500µM
F
fold change/undiff.
RUNX2
RANKL
OPN
PPARg
LPL
FABP4
G
2^(-DCT)
MSCs
Fibroblasts
KITLG
CXCL12
ANGPT1
VEGF
FGF2
IL6
H
pg/ml/number of cells
IL6
VEGF
CXCL12
SCGF-b
I
pg/ml/number of cells
HGF
IL-10
IL-1RA
IL12

Figure S1

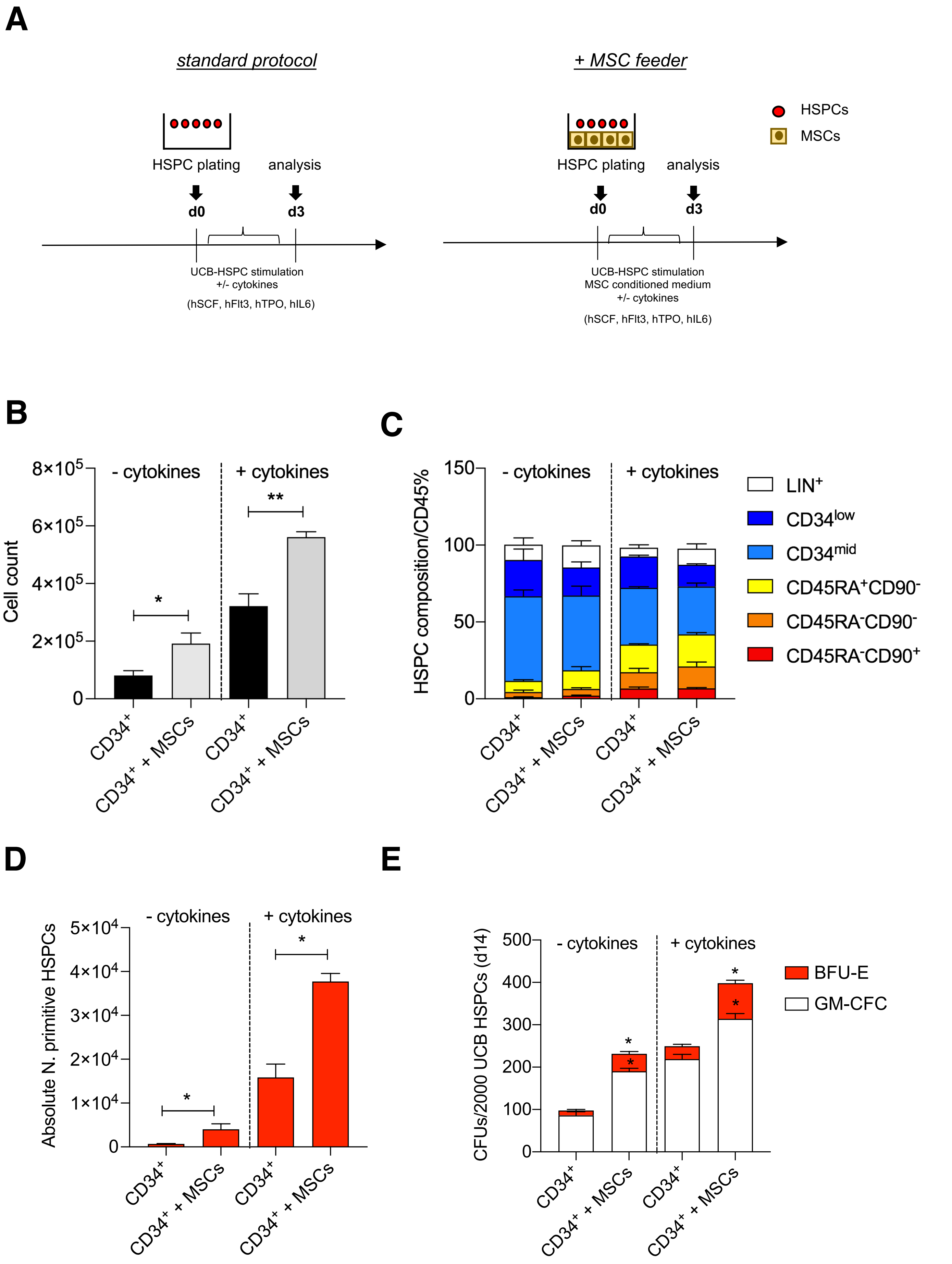
A
standard protocol
+ MSC feeder
HSPCs
MSCs
HSPC plating
analysis
d0
d3
UCB-HSPC stimulation
+/- cytokines
(hSCF, hFlt3, hTPO, hIL6)
UCB-HSPC stimulation
MSC conditioned medium
+/- cytokines
(hSCF, hFlt3, hTPO, hIL6)
B
Cell count
- cytokines
+ cytokines
CD34+
CD34+ + MSCs
C
HSPC composition/CD45%
LIN+
CD34low
CD34mid
CD45RA+CD90-
CD45RA-CD90-
CD45RA-CD90+
D
Absolute N. primitive HSPCs
E
CFUs/2000 UCB HSPCs (d14)
BFU-E
GM-CFC

Figure S2

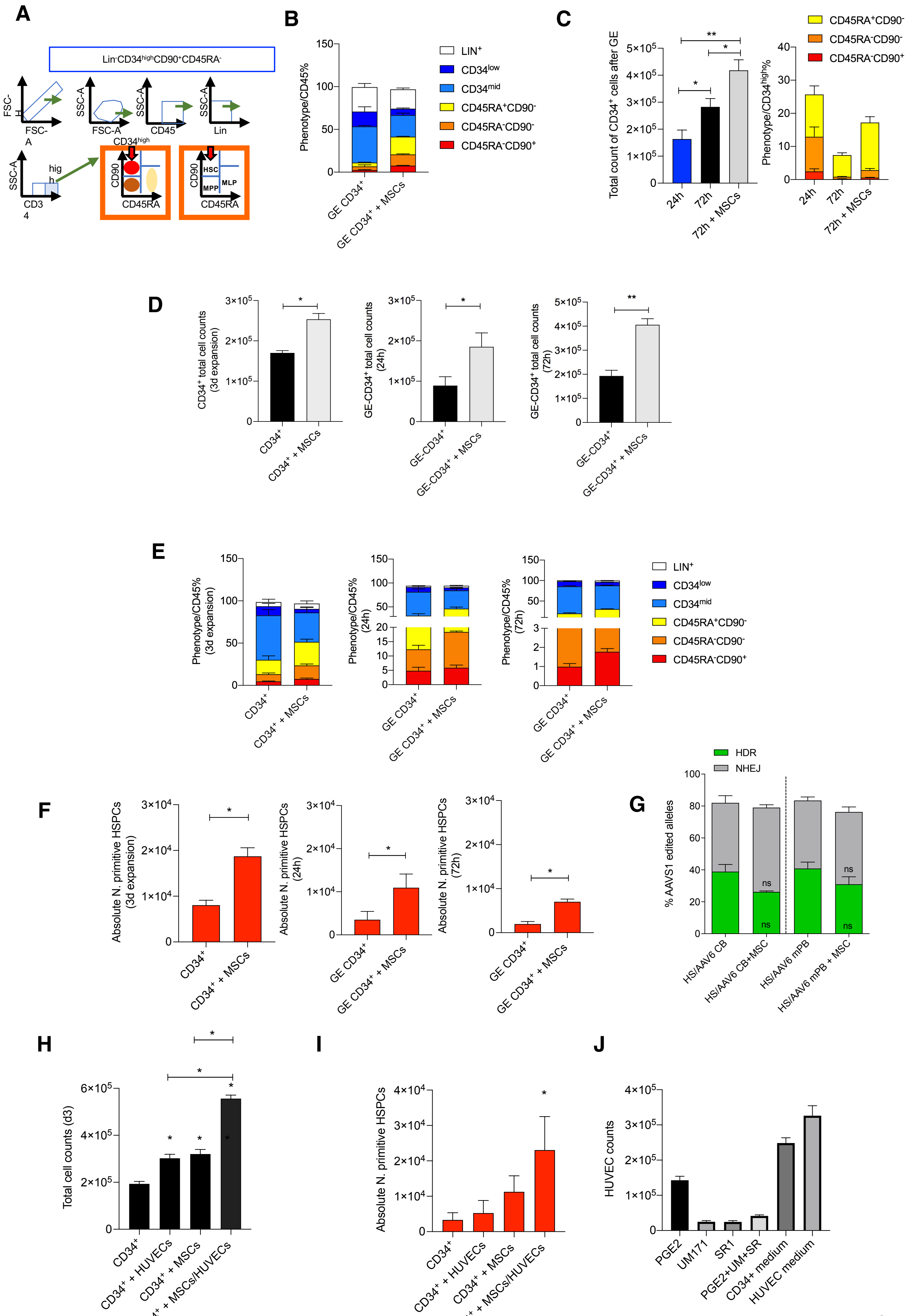
A
Lin-CD34highCD90+CD45RA-
FSC-A
SSC-A
CD45
Lin
CD34
CD34high
CD90
CD45RA
HSC
MLP
MPP
B
Phenotype/CD45%
LIN+
CD34low
CD34mid
CD45RA+CD90-
CD45RA-CD90-
CD45RA-CD90+
GE CD34+
GE CD34+ + MSCs
C
Total count of CD34+ cells after GE
24h
72h
72h + MSCs
Phenotype/CD34high%
D
CD34+ total cell counts (3d expansion)
GE-CD34+ total cell counts (24h)
GE-CD34+ total cell counts (72h)
CD34+
CD34+ + MSCs
GE-CD34+
GE-CD34+ + MSCs
E
Phenotype/CD45% (3d expansion)
Phenotype/CD45% (24h)
Phenotype/CD45% (72h)
F
Absolute N. primitive HSPCs (3d expansion)
Absolute N. primitive HSPCs (24h)
Absolute N. primitive HSPCs (72h)
G
HDR
NHEJ
% AAVS1 edited alleles
HS/AAV6 CB
HS/AAV6 CB+MSC
HS/AAV6 mPB
HS/AAV6 mPB + MSC
ns
H
Total cell counts (d3)
CD34+
CD34+ + HUVECs
CD34+ + MSCs
CD34+ + MSCs/HUVECs
I
Absolute N. primitive HSPCs
J
HUVEC counts
PGE2
UM171
SR1
PGE2+UM+SR
CD34+ medium
HUVEC medium

Figure S3

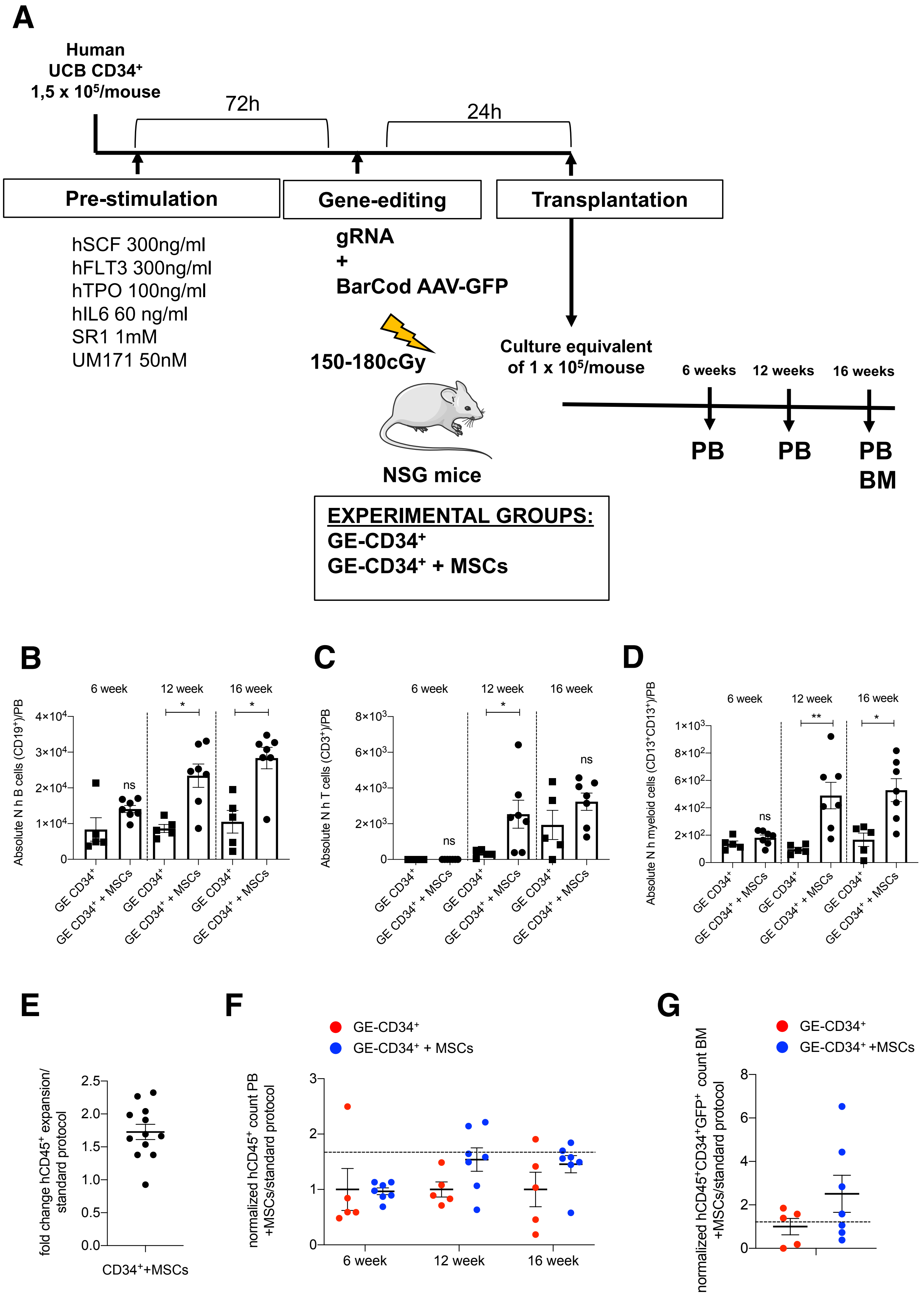
A
Human
UCB CD34+
1,5 x 10^5/mouse
72h
24h
Pre-stimulation
Gene-editing
Transplantation
hSCF 300ng/ml
hFLT3 300ng/ml
hTPO 100ng/ml
hIL6 60 ng/ml
SR1 1mM
UM171 50nM
gRNA
+
BarCod AAV-GFP
150-180cGy
NSG mice
Culture equivalent
of 1 x 10^5/mouse
6 weeks
12 weeks
16 weeks
PB
PB
PB
BM
EXPERIMENTAL GROUPS:
GE-CD34+
GE-CD34+ + MSCs
B
Absolute N h B cells (CD19+)/PB
6 week
12 week
16 week
ns
GE CD34+
GE CD34+ + MSCs
C
Absolute N h T cells (CD3+)/PB
D
Absolute N h myeloid cells (CD13+CD13+)/PB
E
fold change hCD45+ expansion/
standard protocol
CD34++MSCs
F
GE-CD34+
GE-CD34+ + MSCs
normalized hCD45+ count PB
+MSCs/standard protocol
G
GE-CD34+
GE-CD34+ +MSCs
normalized hCD45+CD34+GFP+ count BM
+MSCs/standard protocol

Figure S4

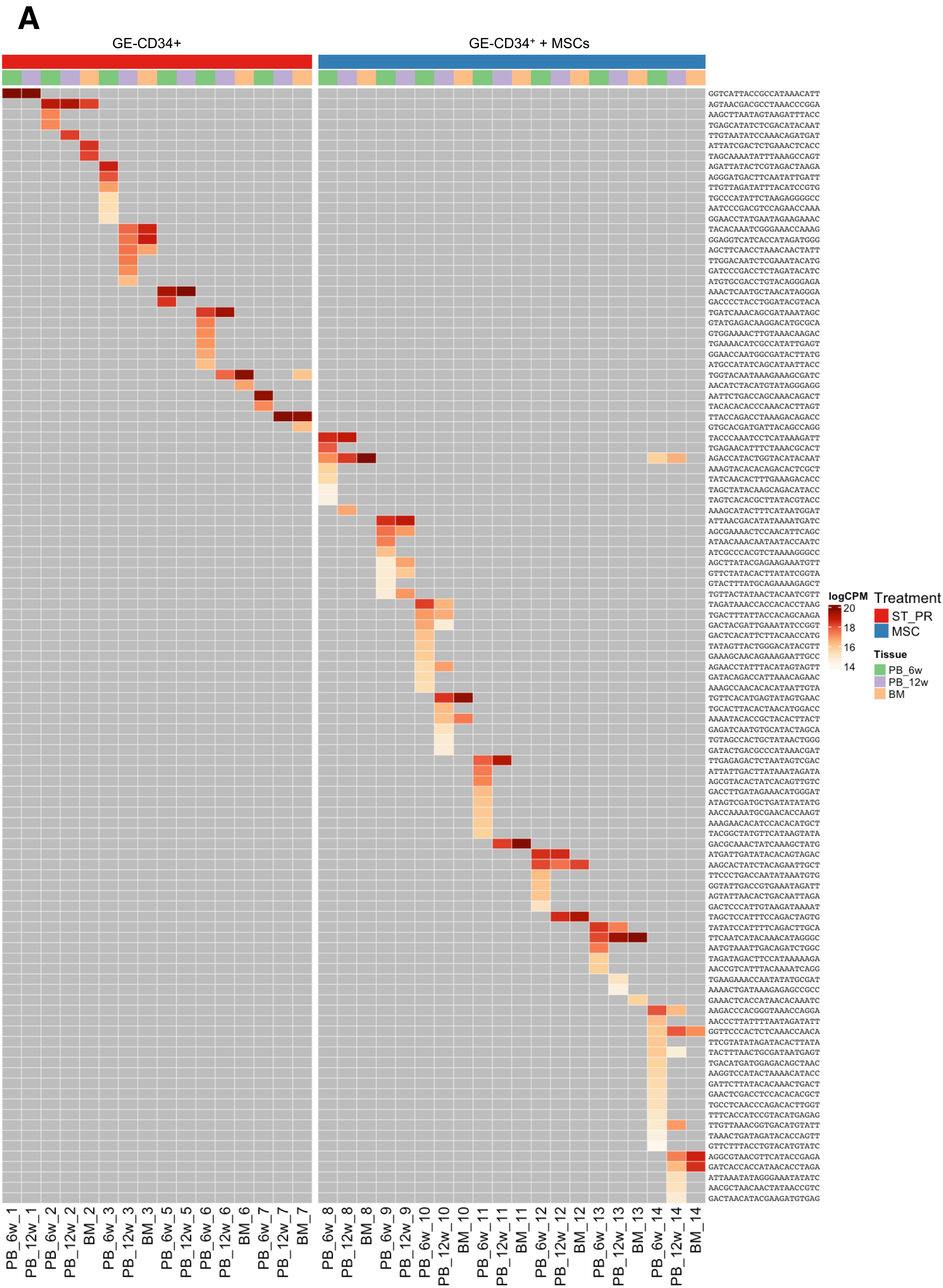
A
GE-CD34+
GE-CD34⁺ + MSCs
GGTCATTACCGCCATAAACATT
AGTAACGACGCCTAAACCCGGA
AAGCTTAATAGTAAGATTTACC
TGAGCATATCTCGACATACAAT
TTGTAATATCCAAACAGATGAT
ATTATCGACTCTGAAACTCACC
TAGCAAAATATTTAAAGCCAGT
AGATTATACTCGTAGACTAAGA
AGGGATGACTTCAATATTGATT
TTGTTAGATATTTACATCCGTG
TGCCCATATTCTAAGAGGGGCC
AATCCCGACGTCCAGAACCAAA
GGAACCTATGAATAGAAGAAAC
TACACAAATCGGGAAACCAAAG
GGAGGTCATCACCATAGATGGG
AGCTTCAACCTAAACAACTATT
TTGGACAATCTCGAAATACATG
GATCCCGACCTCTAGATACATC
ATGTGCGACCTGTACAGGGAGA
AAACTCAATGCTAACATAGGGA
GACCCCTACCTGGATACGTACA
TGATCAAACAGCGATAAATAGC
GTATGAGACAAGGACATGCGCA
GTGGAAAACTTGTAAACAAGAC
TGAAAACATCGCCATATTGAGT
GGAACCAATGGCGATACTTATG
ATGCCATATCAGCATAATTACC
TGGTACAATAAAGAAAGCGATC
AACATCTACATGTATAGGGAGG
AATTCTGACCAGCAAACAGACT
TACACACACCCAAACACTTAGT
TTACCAGACCTAAAGACAGACC
GTGCACGATGATTACAGCCAGG
TACCCAAATCCTCATAAAGATT
TGAGAACATTTCTAAACGCACT
AGACCATACTGGTACATACAAT
AAAGTACACACAGACACTCGCT
TATCAACACTTTGAAAGACACC
TAGCTATACAAGCAGACATACC
TAGTCACACGCTTATACGTACC
AAAGCATACTTTCATAATGGAT
ATTAACGACATATAAAATGATC
AGCGAAAACTCCAACATTCAGC
ATAACAAACAATAATACCAATC
ATCGCCCACGTCTAAAAGGGCC
AGCTTATACGAGAGAAATGTT
GTTCTATACACTTATATCGGTA
GTACTTTATGCAGAAAAGAGCT
TGTTACTATAACTACAATCGTT
TAGATAAACCACCACACCTAAG
TGACTTTATTACCACAGCAAGA
GACTACGATTGAAATATCCGGT
GACTCACATTCTTACAACCATG
TATAGTTACTGGGACATACGTT
GAAAGCAACAGAAAGAATTGCC
AGAACCTATTTACATAGTAGTT
GATACAGACCATTAAACAGAAC
AAAGCCAACACACATAATTGTA
TGTTCACATGAGTATAGTGAAC
TGCACTTACACTAACATGGACC
AAAATACACCGCTACACTTACT
GAGATCAATGTGCATACTAGCA
TGTAGCCACTGCTATAACTGGG
GATACTGACGCCCATAAACGAT
TTGAGAGACTCTAATAGTCGAC
ATTATTGACTTTATAAATAGATA
AGCGTACACTATCACAGTTGTC
GACCTTGATAGAAACATGGGAT
ATAGTCGATGCTGATATATATG
AACCAAAATGCGAACACCAAGT
AAAGAACACATCCACACATGCT
TACGGCTATGTTCATAAGTATA
GACGCAAACTATCAAAGCTATG
ATGATTGATATACACAGTAGAC
AAGCACTATCTACAGAATTGCT
TTCCCTGACCAATATAAATGTG
GGTATTGACCGTGAAATAGATT
AGTATTAACACTGACAATTAGA
GACTCCCATTGTAGATAAAAT
TAGCTCCATTTCCAGACTAGTG
TATATCCATTTTCAGACTTGCA
TTCAATCATACAAACATAGGGC
AATGTAAATTGACAGATCTGGC
TAGATAGACTTCCATAAAAAGA
AACCGTCATTTACAAAATCAGG
TGAAGAAACCAATATATGCGAT
AAAACTGATAAAGAGAGCCGCC
GAAACTCACCATAACACAAATC
AAGACCCACGGGTAAACCAGGA
AACCCTATTTTATAGATATT
GGTTCCCACTCTCAAACCAACA
TTCGTATATAGATACACTTATA
TACTTTAACTGCGATAATGAGT
TGACATGATGGAGACAGCTAAC
AAGGTCCATACTAAAACATACC
GATTCTTATACACAAACTGACT
GAACTCGACCTCCACACACGCT
TGCCTCAACCCAGACACTTGGT
TTTCACCATCCGTACATGAGAG
TTGTTAAACGGTGACATGTATT
TAAACTGATAGATACACCAGTT
GTTCTTTACCTGTACATGTATC
AGGCGTAACGTTCATACCGAGA
GATCACCACCATAACACCTAGA
ATTAAATATAGGGAAATATATC
AACGCTAACAACTATAACCGTC
GACTAACATACGAAGATGTGAG
PB_6w_1
PB_12w_1
PB_6w_2
PB_12w_2
BM_2
PB_6w_3
PB_12w_3
BM_3
PB_6w_5
PB_12w_5
PB_6w_6
PB_12w_6
BM_6
PB_6w_7
PB_12w_7
BM_7
PB_6w_8
PB_12w_8
BM_8
PB_6w_9
PB_12w_9
PB_6w_10
PB_12w_10
BM_10
PB_6w_11
PB_12w_11
BM_11
PB_6w_12
PB_12w_12
BM_12
PB_6w_13
PB_12w_13
BM_13
PB_6w_14
PB_12w_14
BM_14
logCPM
20
18
16
14
Treatment
ST_PR
MSC
Tissue
PB_6w
PB_12w
BM

Figure S5

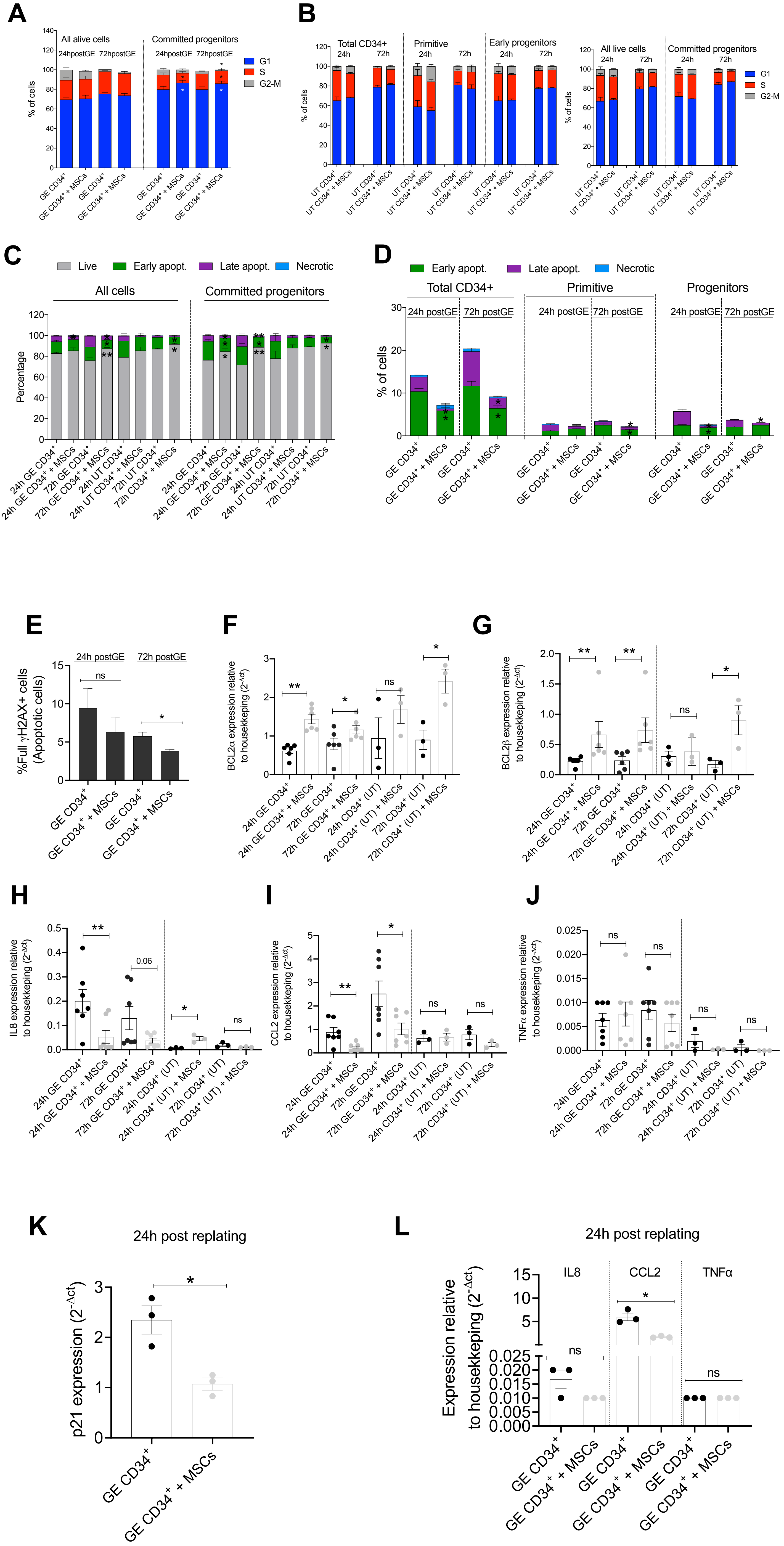
A
All alive cells
Committed progenitors
24hpostGE
72hpostGE
% of cells
G1
S
G2-M
GE CD34+
GE CD34+ + MSCs
B
Total CD34+
Primitive
Early progenitors
All live cells
Committed progenitors
24h
72h
UT CD34+
UT CD34+ + MSCs
C
Live
Early apopt.
Late apopt.
Necrotic
All cells
Committed progenitors
Percentage
24h GE CD34+
24h GE CD34+ + MSCs
72h GE CD34+
72h GE CD34+ + MSCs
24h UT CD34+
24h UT CD34+ + MSCs
72h UT CD34+
72h CD34+ + MSCs
D
Total CD34+
Primitive
Progenitors
24h postGE
72h postGE
% of cells
E
%Full γH2AX+ cells (Apoptotic cells)
ns
F
BCL2α expression relative to housekeping (2-Δct)
G
BCL2β expression relative to housekeping (2-Δct)
H
IL8 expression relative to housekeping (2-Δct)
0.06
I
CCL2 expression relative to housekeping (2-Δct)
J
TNFα expression relative to housekeping (2-Δct)
24h CD34+ (UT)
24h CD34+ (UT) + MSCs
72h CD34+ (UT)
72h CD34+ (UT) + MSCs
K
24h post replating
p21 expression (2-Δct)
L
24h post replating
IL8
CCL2
TNFα
Expression relative to housekeping (2-Δct)

Figure S6

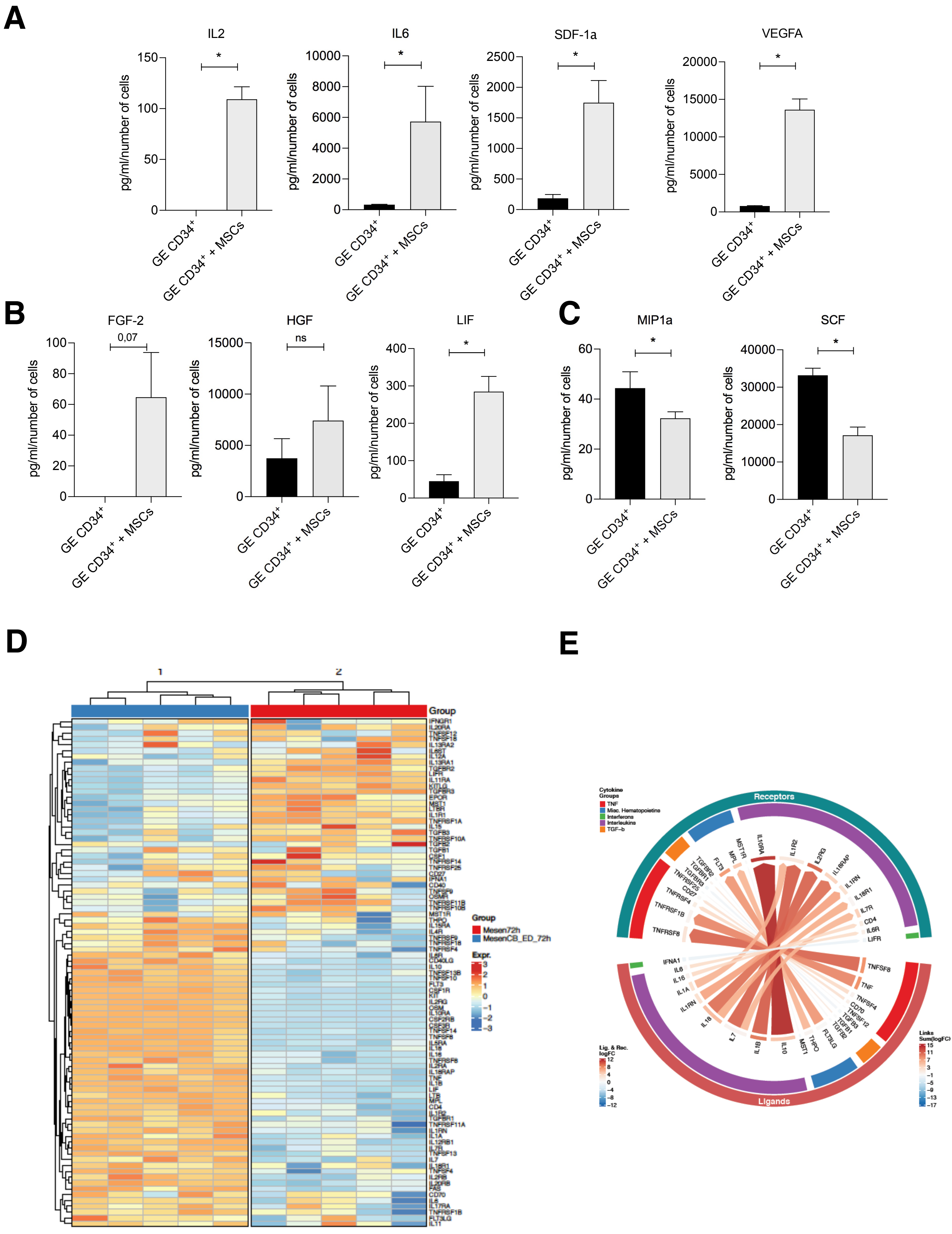
A
IL2
IL6
SDF-1a
VEGFA
pg/ml/number of cells
GE CD34+
GE CD34+ + MSCs
*
B
FGF-2
HGF
LIF
0,07
ns
C
MIP1a
SCF
D
Group
Mesen72h
MesenCB_ED_72h
Expr.
E
Cytokine Groups
TNF
Misc. Hematopoietins
Interferons
Interleukins
TGF-b
Receptors
Ligands
Lig. & Rec. logFC
Links Sum(logFC)

Figure S7

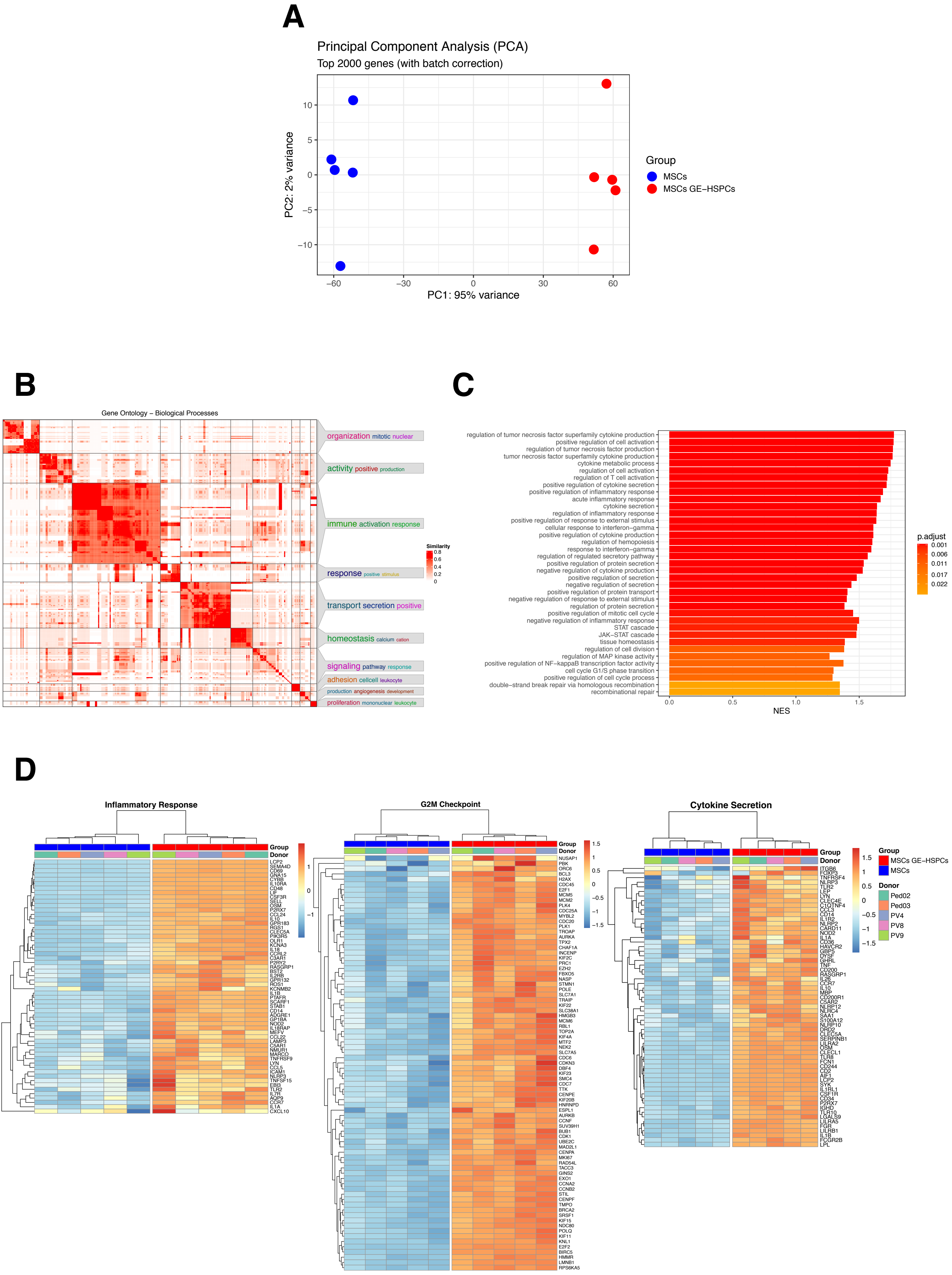
A
Principal Component Analysis (PCA)
Top 2000 genes (with batch correction)
PC2: 2% variance
PC1: 95% variance
10
5
0
−5
−10
−60
−30
0
30
60
Group
MSCs
MSCs GE−HSPCs
B
Gene Ontology − Biological Processes
Similarity
0.8
0.6
0.4
0.2
0
C
regulation of tumor necrosis factor superfamily cytokine production
positive regulation of cell activation
regulation of tumor necrosis factor production
tumor necrosis factor superfamily cytokine production
cytokine metabolic process
regulation of cell activation
regulation of T cell activation
positive regulation of cytokine secretion
positive regulation of inflammatory response
acute inflammatory response
cytokine secretion
regulation of inflammatory response
positive regulation of response to external stimulus
cellular response to interferon−gamma
positive regulation of cytokine production
regulation of hemopoiesis
response to interferon−gamma
regulation of regulated secretory pathway
positive regulation of protein secretion
negative regulation of cytokine production
positive regulation of secretion
negative regulation of secretion
positive regulation of protein transport
negative regulation of response to external stimulus
regulation of protein secretion
positive regulation of mitotic cell cycle
negative regulation of inflammatory response
STAT cascade
JAK−STAT cascade
tissue homeostasis
regulation of cell division
regulation of MAP kinase activity
positive regulation of NF−kappaB transcription factor activity
cell cycle G1/S phase transition
positive regulation of cell cycle process
double−strand break repair via homologous recombination
recombinational repair
0.0
0.5
1.0
1.5
NES
p.adjust
0.001
0.006
0.011
0.017
0.022
D
Inflammatory Response
G2M Checkpoint
Cytokine Secretion
Group
Donor
MSCs GE−HSPCs
MSCs
Ped02
Ped03
PV4
PV8
PV9

Figure S8

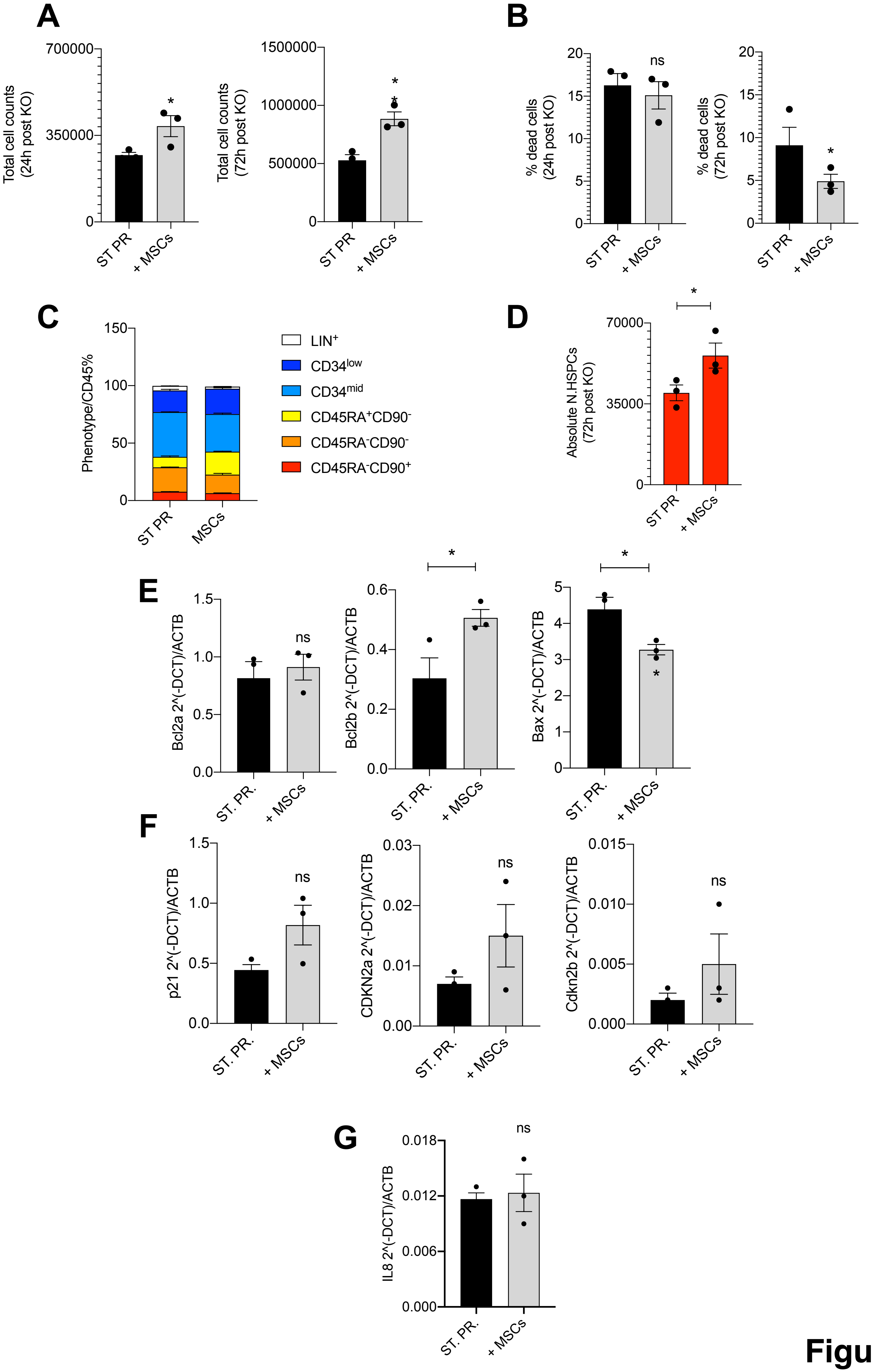
A
Total cell counts (24h post KO)
Total cell counts (72h post KO)
B
% dead cells (24h post KO)
% dead cells (72h post KO)
ns
ST PR
+ MSCs
C
Phenotype/CD45%
LIN+
CD34low
CD34mid
CD45RA+CD90-
CD45RA-CD90-
CD45RA-CD90+
MSCs
D
Absolute N.HSPCs (72h post KO)
E
Bcl2a 2^(-DCT)/ACTB
Bcl2b 2^(-DCT)/ACTB
Bax 2^(-DCT)/ACTB
ST. PR.
F
p21 2^(-DCT)/ACTB
CDKN2a 2^(-DCT)/ACTB
Cdkn2b 2^(-DCT)/ACTB
G
IL8 2^(-DCT)/ACTB

Figure S9

Supplementary Figure Legend

Fig. S1. MSC isolation and characterization.

Fig. S2. MSCs support the expansion of UCB-HSPCs while preserving the HSPC subset with a primitive phenotype.

Fig. S3. Phenotypic analysis of *ex-vivo* expanded UCB-HSPCs.

Fig. S4. Analysis of hematological reconstitution in transplanted NSG mice.

Fig. S5. Analysis of clonal reconstitution dynamics in transplanted NSG mice.

Fig. S6. Analysis of cell-cycle, survival and inflammatory state in GE-HSPCs.

Fig. S7. Cytokine analysis of conditioned medium from MSCs co-cultured with GE-HSPCs.

Fig. S8. RNA sequencing analysis of MSCs compared to MSCs co-cultured with GE-HSPCs.

Fig. S9. MSCs in support of KO-edited HSPCs.

Fig. S1: Isolation and characterization of BM-MSCs. A) Clonogenic capacity of BM-MSCs used in this study, determined as CFU-Fs at 7 and 14 days after plating the CD34 negative cellular fraction of healthy donor BM aspirates. Each error bars show means ± s.e.m of CFU-Fs normalized for the number of plated cells. n=6. **B**) Representative picture of *ex-vivo* expanded MSCs acquiring a spindle-like morphology in culture. **C**) Proliferation capacity of MSCs evaluated as population doubling time (PDT)/day starting from passage 3. Each error bars show means ± s.e.m of PDT calculated as (https://www.doubling-time.com/compute.php). **D**) Representative flow cytometer analysis used to characterize the expression of canonical MSC markers (CD90, CD73, CD105) and lack of expression of hematopoietic (CD34, CD45, CD14), MHC class II (HLA-DR) and endothelial markers (CD31) in the MSC samples used in this study. **E**) Representative pictures of MSCs differentiated into osteoblasts (Alizarin red, left panel), adipocytes (Oil Red O, middle panel) and chondrocytes (Safranin O-Fast Green, right panel). **F**) RT-qPCR expression analysis of early (*PPARγ*) and late genes (LPL, FABP4) of adipogenic differentiation in MSCs cultured in proper adipogenic differentiation medium for 21 days. Expression analysis of early (*RUNX2*) and late (*RANKL*, *OPN*) genes of osteogenic differentiation in MSCs differentiated for 21 days in osteogenic medium in reported in the right panel. Each error bars show means ± s.e.m gene expression calculated as $2^{-\Delta CT}$ relative to Actin-beta (*ACTB*) gene (left panel). **G**) Fold change of hematopoietic supportive gene expression in BM-derived MSCs (black bars) compared to fibroblasts (grey bars) expanded in culture for 3-4 passages. Each error bars show means ± s.e.m. of gene expression calculated as $2^{-\Delta CT}$ relative to *ACTB* gene expression. Each error bars show means ± s.e.m ($n \geq 3$). *p*-values were determined by Mann-Whitney test (*$p \leq 0.05$). (n =7). Quantification (pg/ml) of hematopoietic supportive cytokines (**H**) and inflammatory molecules (**I**) in the conditioned medium from MSCs expanded for 72 hours in culture. Each error bars show means ± s.e.m. normalized for the number of cells originally plated (n = 6).

Fig. S2. MSCs support the expansion of UCB-HSPCs while preserving the HSPC subset with a primitive phenotype. A) Schematic representation of the cell culture protocol used to expand UCB-HSPCs on MSC feeder. HSPCs expanded on plastic using standard protocols [5] were used as controls. **B**) Human HSPC ($CD34^+$) total cell count after 72 hours of culture with (+ cytokines) or without (-cytokines) the addition of proper cytokines in the medium (hSCF, hFLT3, hTPO, hIL-6) [5]. Grey bars: UCB-HSPCs co-cultured with MSCs; black bars: UCB-HSPCs cultured in plastic dishes. Each error bars show means ± s.e.m. of total cell count (n=5). *p*-values were determined by Mann-Whitney test (*p ≤ 0.05; **p ≤ 0.001). **C**) Phenotypic composition of UCB-HSPCs expanded for 72 hours in culture with (+ cytokines) or without (-cytokines) the addition of proper cytokines in the medium on MSC feeder ($CD34^+$ + MSCs) or according to standard protocols ($CD34^+$). The primitive HSPC subset was phenotypically defined as $CD45^+$, $CD34^{high}$, $CD90^+$, $CD45RA^-$ subset. Each error bars show means ± s.e.m. of percentage of each subset to the percentage of human $CD45^+$ cells (n = 4). **D**) Absolute number of HSPCs with a primitive phenotype present in culture after 72 hours of expansion (n=4). Absolute number was calculated as percentage of $CD45^+$, $CD34^{high}$, $CD90^+$, $CD45RA^-$ of total cell counts (**B**). Each error bars show means ± s.e.m. *p*-values were determined by Mann-Whitney test (*p ≤ 0.05). **E**) CFUs assay 14 days after plating *ex vivo* expanded UCB-HSPCs into methylcelluose medium. Red = Burst Forming Units-Erythroid; white = granulocyte-macrophage progenitors. Each error bars show means ± s.e.m. (n = 4). *p*-values were determined by Mann-Whitney test (*p ≤ 0.05).

Fig. S3. Phenotypic analysis of *ex-vivo* expanded UCB-HSPCs. A) Schematic representation of the gating strategy developed to phenotypically identify primitive HSPC in culture. **B**) Phenotypic analysis of GE-HSPCs expanded on MSC feeder for 72h (GE $CD34^+$ + MSCs) after gene-editing. GE-HSPCs expanded on plastic were used as controls (GE $CD34^+$). Each error bars show means ± s.e.m. of the percentage of each phenotypic subset on human $CD45^+$ cells (n=4). **C**) Total cell count

(left panel) and phenotypic analysis (right panel) of the primitive HSPC subset in UCB-HSPCs cultured for 72 hours upon gene editing in the presence or absence of an MSC feeder compared to GE-HPSCs recovered on plastic for 24 hours. **D**) Total cell counts of UCB-HSPCs co-cultured with MSCs ($CD34^+$ + MSCs) for 3 days before editing (left panel). Total cell counts of GE-HSPCs recovered in culture for 24 hours and expanded for 72 hours after editing in the presence (GE $CD34^+$ + MSCs) or absence (GE $CD34^+$) of MSC feeder are shown in the middle and right panel, respectively. For each condition, the phenotypic analysis of cultured $CD34^+$ is reported in **E**. **F**) Absolute number of HSPCs with a primitive phenotype ($CD45^+$, $CD34^{high}$, $CD90^+$, $CD45RA^-$) before editing (left panel), at 24 hours after editing (middle panel) and 72 hours after editing (right panel). The absolute number of HSPCs with a primitive phenotype in co-culture with MSCs (grey bars) was compared to the ones observed using our standard protocol (black bars) of gene-editing. **G**) Percentage of HDR and NHEJ 72 hours after editing UCB (CB) and mobilized peripheral blood (mPB) $CD34^+$ cells edited in the presence or not of MSCs (n = 3); Wilcoxon matched-pairs signed rank test. Total cell counts (**H**) and absolute number of phenotypically primitive HSPCs (**I**) after 72-hour co-culture of $CD34^+$ cells in the presence of MSCs, HUVEC, and mixed MSC-HUVEC feeder. $CD34^+$ cells expanded on plastic were used as controls. **J**) HUVEC cell counts 72 hours after the exposure to the gene-editing drugs required by our protocol for efficient gene transfer. For all the experiments, each error bars show means ± s.e.m ($n \geq 3$). *p*-values were determined by Mann-Whitney test (*$p \leq 0.05$).

Fig. S4. Flow cytometry analysis of hematological reconstitution in NSG transplanted mice. A) Schematic representation of the experimental plan used for the transplantation of the culture outgrowing cells of UCB-HSPCs gene editing in the presence of MSCs (GE CD34+ + MSCs) or according to standard protocol (GE CD34+) into NSG mice. Absolute number of human B cells ($CD19^+$) (**B**), T cells ($CD3^+$) (**C**) and myeloid cells ($CD13^+$, $CD33^+$) (**D**) on total $hCD45^+$ cells in the peripheral blood (PB) of NSG mice transplanted with HSPCs gene-edited in the presence of MSCs

(+MSCs) or according to standard protocol (st.pr) at different time points post transplantation. **E**) Ratio between the number of outgrowing cells effectively transplanted upon gene editing in the presence of MSCs and the number of cells gene-edited according to our standard protocol. **F**) Fold change of human engraftment in the peripheral blood of transplanted mice calculated as ratio of human $CD45^+$ cell counts normalized for the number of effectively transplanted cells in mice treated with GE-HPSCs + MSCs compared to standard GE-HSPC transplants. **G**) Fold change of $CD45^+CD34^+GFP^+$ cell engraftment in the BM of transplanted mice represented as ratio of $CD45^+CD34^+GFP^+$ absolute counts normalized for the number of transplanted cells in mice transplanted with GE-HSPCs+MSCs compared to standard GE-HSPCs. For all plots, individual data points represent an animal. *p*-values were determined by Mann-Whitney test (*$p \leq 0.05$;** $p \leq 0.01$).

Fig. S5. Clonal tracking analysis. **A**) Heatmap showing the logCPM abundance (red-scaled palette) of dominant unique "barcode" sequences (rows) in the PB at 6 and 12 weeks after transplantation and in the BM at sacrifice of mice transplanted with HSPCs gene-edited in presence of MSCs or standard GE-HSPCs.

Fig. S6. Characterization of the DDR response in GE-HSPCs. **A**) Percentage of committed progenitors in the indicated cell-cycle phase at 24 (n = 8) and 72 hours (n = 7) after gene-editing. Significance was calculated for each time point comparing GE $CD34^+$ + MSCs to GE $CD34^+$ at each cell-cycle phase. Each error bars show means ± s.e.m. p-values were determined by Mann-Whitney test (only significant comparisons were reported). **B**) Cell-cycle analysis at the indicated time points in untreated (UT) and gene-edited (GE) committed progenitors. Live: Annexin V^-, $7AAD^-$, Early apoptotic: Annexin V^+, $7AAD^-$, Late apoptotic: Annexin V^+, $7AAD^+$, Necrotic: Annexin V^-, $7AAD^+$ ($n \geq 3$ for each time point). Each error bars show means ± s.e.m. p-values were determined by Mann-

Whitney test for each apoptotic phase (only significant comparisons were reported; *$p < 0.05$). **C**) Apoptosis analysis at the indicated time points in untreated (UT) and gene-edited (GE) committed progenitors. Live: Annexin V^-, $7AAD^-$, Early apoptotic: Annexin V^+, $7AAD^-$, Late apoptotic: Annexin V^+, $7AAD^+$, Necrotic: Annexin V^-, $7AAD^+$ ($n \geq 3$ for each time point). Each error bars show means ± s.e.m. p-values were determined by Mann-Whitney test for each apoptotic phase (only significant comparisons were reported; *$p < 0.05$). **D**) Apoptosis analysis at the indicated time points in the different subsets of gene-edited (GE) HSPCs. Early apoptotic: Annexin V+, 7AAD-, Late apoptotic: Annexin V+, 7AAD+, Necrotic: Annexin V-, 7AAD+ ($n \geq 3$ for each time point). Each error bars show means ± s.e.m. p-values were determined by Mann-Whitney test for each apoptotic phase (only significant comparisons were reported; *$p < 0.05$). **E**) Quantification of full-stained γH2AX positive cells from Fig. 3A-C. (n=4 for each time point). Each error bars show means ± s.e.m. p-values were determined by Mann-Whitney test comparing GE $CD34^+$ + MSCs to GE $CD34^+$ (ns $p>0.05$; *$p < 0.05$). RT-qPCR expression analysis of *BCL2α* (**F**) and *BCL2β* (**G**) in untreated (UT) and gene-edited HSPCs in the presence or absence of MSC feeder after 24 and 72 hours of culture. RT-qPCR expression analysis of *IL-8* (**H**), *CCL2* (**I**) and *TNFα* (**J**) in untreated (UT) and gene-edited (GE) HSPCs co-cultured with MSCs (+ MSCs) for 24 and 72 hours compared to our standard protocol condition. Expression analysis of *p21* (**K**), *IL-8*, *CCL2*, and *TNFα* (**L**) in GE-HSPCs maintained in culture for 24 hours in a medium poor of cytokines after 72 hour-expansion in the presence (GE $CD34^+$ + MSCs) or absence (GE $CD34^+$) of MSCs. For all the experiments, error bars show means ± s.e.m. and the level of gene expression was calculated as 2 -ΔCT relative to *GUSB* gene ($n \geq 3$ for each time point). p-values were determined by Mann-Whitney test comparing GE $CD34^+$ + MSCs to GE $CD34^+$ and comparing UT $CD34^+$ + MSCs to UT $CD34^+$(ns $p>0.05$; *$p < 0.05$).

Fig. S7. Cytokine analysis of the conditioned from the co-culture of GE-HSPCs with MSCs. Quantification (pg/ml) of hematopoietic supportive cytokines (**A**) and growth factors (**B**) in the

conditioned medium from MSCs co-cultured with GE-HSPCs for 72 hours. Conditioned medium from GE-HSPCs expanded in plastic dishes for 72 hours was used as control. Quantification was normalized for the number of plated cells. MIP1a and SCF were the only two factors downregulated in the co-culture medium (**C**). Each error bars show means ± s.e.m. (n=4). p-values were determined by Mann-Whitney test (*$p \leq 0.05$). **D**) Heatmap representation of gene expression analysis of cytokines in MSCs (n=5) and MSC co-cultured with GE-HSPCs (n=5) with unsupervised clustering showing the separation of the two groups. **E**) Chord diagram showing the results of the DGE analysis on ligand-receptor cytokine pairs. For each pair, the logFC values resulting from the comparison of MSC with MSC co-cultured with GE-HSPCs samples are reported, while links represent the score of the pairs computed by summing the logFC values of both ligands and receptors. Cytokine pairs are organized into groups based on their function: TNF, Miscellaneous Hematopoietins, Interferons, Interleukins, and TGF-β.

Fig. S8. RNA sequencing analysis of MSCs compared to MSCs co-cultured with GE-HSPCs. A) Principal component analysis (PCA) plot of the RNA-seq samples. **B**) Heatmap showing the similarity of significant Gene Ontology (BP) terms resulting from the Gene Set Enrichment Analysis (GSEA). **C**) Bar plot of the inflammation, cell cycle, and cytokine production GO terms with the corresponding NES values. Colors represent adjusted p-values. **D**) Heatmap of regularized log-normalized read counts of GSEA core-enriched genes of the inflammatory response hallmark category (left panel). Heatmap of regularized log-normalized read counts of GSEA core-enriched genes of the G2M checkpoint hallmark category (middle panel). Heatmap of regularized log-normalized read counts of GSEA core-enriched genes of the cytokine secretion category (right panel).

Fig. S9. MSCs in support of KO-edited HSPCs. Total cell count (**A**) and percentage of death (**B**) of KO-edited HSPCs recovered for 24 (left panel) and 72 hours (right panel) on a MSC feeder

(+MSCs) or on plastic according to our standard protocol (ST. PR.). Phenotypic analysis (**C**) and absolute number of phenotypically primitive (**D**) KO-HSPCs 72 hours after editing in the presence (+MSCs) or absence (ST. PR.) of MSCs. **E-G**) Gene expression analysis of pro-survival (*BCL2*α, *BCL2*β), apoptotic (*BAX*), cell-cyle (*p21*, *CDKN2A*, *CDKN2B*) and inflammatory genes (*IL-8*) in KO-edited HSPCs recovered in culture for 72 hours after editing on a MSC feeder or according to our standard protocol conditions. For all the experiments, each error bars show means ± s.e.m ($n \geq 3$). *p*-values were determined by Mann-Whitney test (*$p \leq 0.05$).

Supplementary Tables

Table S1. List of differentially expressed gene (DGEs) in MSCs exposed to GE-HSPCs compared to MSCs.

Table S2. List of the significantly enriched GO terms derived from the GSEA of the pre-ranked gene list resulting from the comparison between MSCs and MSCs co-cultured with GE-HSPCs.

Supplementary Materials

Material and Methods.

Cell Culture. BM-MSCs were cultured in DMEM+GlutaMAX (Thermo Fisher Scientific, 10566-016) supplemented with 5% platelet lysate (Stemcell, 06963) and 1% penicillin/streptomycin (Gibco, 15140122). UCB $CD34^+$ cells (Lonza, 2C-101) were cultured in Stemspan-SFEM II (Stemcell Technologies, 09650), supplemented with 1% penicillin/streptomycin, 2% L-glutamine (Gibco, A2916801), human recombinant thrombopoietin (TPO) (20ng/ml) (PeproTech, 300-18), interleukin-6 (IL-6) (20ng/ml) (PeproTech, 200-06), Flt3 ligand (Flt3L) (100 ng/ml) (PeproTech, 300-19) and stem cell factor (SCF) (100 ng/ml) (PeproTech, 300-07). PGE2 (10 ug/ml) (Cayman, 14750) was added to facilitate the recovering of thawed cells. After editing, pyrimidoindole derivative (UM171) (50nM) (Stemcell, 72912) and StemRegenin 1 (SR1) (1 uM) (Biovision, 1967) were added as supplement in Stemspan medium. HUVEC cells were cultured in M199 medium (ThermoFisher Scientific, 11150059) supplemented with 20% fetal bovine serum (FBS) (ThermoFisher Scientific, A4766801), 1% penicillin/streptomycin, 2 mM L-glutamine, 10 mM 4-(2-hydroxyethyl)-1-piperazineethanesulfonic acid (HEPES) (ThermoFisher Scientific, 15630080), 50 mg/ml heparin (Stemcell Technologies, 07980) and 50 mg/ml endothelial cell growth supplement (ECGS) (Merck, E2759).

Fibroblast colony-forming unit assay. The CD34 negative fraction of BM mononuclear cells was plated at a density of 2×10^5 cells/cm^2 in DMEM+GlutaMAX supplemented with 5% platelet lysate and 1% penicillin/streptomycin. Colony-forming units were stained with 1% crystal violet (Sigma-Aldrich, catalog C0775) and manually counted after 7 and 14 days.

Population doubling time. BM-MSCs were plated at a density of 3×10^4 cells/cm^2, detached, and counted when reaching 80%–90% of confluence using Trypan blue to distinguish live cells. Proliferative capacity was calculated as population doubling time according to http://www.doubling-time.com/ compute.php. Cell proliferation was followed from passage 3 to 6.

Flow cytometry MSC characterization. MSCs were immunophenotypically characterized by flow-cytometry (FC) at passage 2/3 to evaluate the expression of MSC canonical markers. Cells were detached and washed with PBS + 2% FBS. 1×10^5 cells were incubated with the proper antibody mix for 10 minutes at RT in the dark. The following antibodies were used: CD90 PE (BioLegend, 328110), CD105 FITC (BioLegend, 323204), CD73 PE (BD Biosciences 555445), CD146 V450 (BD Biosciences, 562136), CD271 PE (BD Biosciences, 557196), CD45 APC (BD Biosciences, 340910), CD34 FITC (BD Biosciences, 345801), CD14 APC (BD Biosciences, 555399), CD31 FITC (BD Biosciences, 55027) and HLA-DR PB (BioLegend, 307624). Unstained cells were used as negative control. All samples were run on BD FACSCanto II cytometer (BD Biosciences). At least 10.000 were recorded.

***In vitro* adipogenic, osteogenic and chondrogenic differentiation of BM-MSCs.** For adipogenic and osteogenic differentiation, BM-MSCs were cultured in proper induction medium according to a previously published protocol [1]. Adipogenic differentiation medium: alpha MEM (Thermo Fisher Scientific, 12571), 1% penicillin/streptomycin, and 10% MesenCult MSC Stimulatory Supplement (Stemcell Technologies, 05402), supplemented with 10^{-10} M dexamethasone (Sigma, D4902), 50 µg/ml l-ascorbic acid (Sigma, A92902), 10 µg/ml insulin, 5 µM 3-isobutyl-1-methylxanthine (Sigma, I5879), 0.2^{-10} M indomethacin (Sigma, I7378), and 0.5 mM β-glycerol

phosphate (Sigma, G9422). Osteogenic differentiation medium: alpha MEM, 1% penicillin/streptomycin, and 10% MesenCult MSC Stimulatory Supplement, supplemented with 10^{-10} M dexamethasone and 50 µg/ml l-ascorbic acid. Starting from day 7 of differentiation, 5 mM β-glycerol phosphate was added to the medium. Chondrogenic differentiation of MSCs was obtained by plating MSCs at a concentration of $2.5x10^5$ cells/ml in StemMACS ChondroDiff Media (MiltenyiBiotech, 130-091-679), following the manufacturer's instruction. Detection of chondrocytes was performed on PFA 4% fixed pellets using Safranin O-Fast Green (Sigma, 84120). MSC differentiation was evaluated after 21 days by proper staining and/or gene expression analysis.

Oil Red O staining. BM-MSC-differentiated cells, were fixed with PFA 4% for 15' (Sigma, 1004968350), washed with Isopropanol 60% and stained with Oil Red 0 solution (Merck, 102419) for 15' to evaluate the efficiency of adipogenic differentiation. After washing with H_20, cells were stained with hematoxylin solution for 10' (Sigma, MHS80).

Alizarin Red staining. BM-MSC -differentiated cells, were fixed with PFA 4% for 15' (Sigma, 1004968350), washed with H_20 and stained with Alizarin Red (Merck, TMS-008-C) for 15' to evaluate the efficieny of osteogenic differentiation. After washing with H_20, cells were stained with hematoxylin solution for 10' (Sigma, MHS80).

RNA extraction. RT-PCR and real-time PCR. Total RNA was extracted using RNeasy Micro Kit (Qiagen, 74004) or miRNeasy Micro Kit (Qiagen, 217084), according to the manufacturer's instructions and DNase treatment was performed using RNase-free DNase Set (Qiagen,79254). cDNA was synthesized from 1 µg total RNA using the high-capacity reverse transcription kit (Applied Biosystems, 4319983) or from 30 ng total RNA using iScript cDNA Synthesis Kit (Bio-

Rad, 170-8891) and then pre-amplified using TaqMan PreAmp Master Mix (2X) (ThermoFisher, 4488593). SYBR Green based quantitative PCR was performed using QuantiFast SYBR Green PCR Kit (Qiagen, 1039712) or Fast SYBR Green Master Mix (Thermofisher, 4385618), starting from 10 ng of cDNA with a Viia7 real-time PCR system (Thermofisher). The following primers were used after standard curve method optimization to reach the 100% primer efficiency:

**Gene**	**Primers**
KITLG	FW 5’AGCCAGCTCCCTTAGGAATG 3’<br>RW 5’ GACTTGGCTGTCTCTTCTTCCA 3’
CXCL12	FW 5’ TGCCCTTCAGATTGTAGCCC 3’<br>RW 5’ CGAGTGGGTCTAGCGGAAAG 3’
FGF2	FW 5’GCTGTACTGCAAAAACGGGG 3’<br>RW 5’ TAGCTTGATGTGAGGGTCGC 3’
IL6	FW 5’GATGGATGCTTCCAATCTGG 3’<br>RW 5’ TGTTCTGGAGGTACTCTAGG 3’
VEGFA	FW 5’ CATCTTCAAGCCATCCTGTG 3’<br>RW 5’ GGAAGCTCATCTCTCCTATG 3’
ANGPT1	FW 5’ ACATGGGCAATGTGCCTACA 3’<br>RW 5’ TCTCAAGTTTTTGCAGCCACTG 3’
PPARg	FW 5’ TCAGAAATGCCTTGCAGTGG 3’<br>RW 5’ TATCACTGGAGATCTCCGCCAA 3’
FABP4	FW 5’ AAACTGGTGGTGGAATGCGT 3’<br>RW 5’ GCGAACTTCAGTCCAGGTCA 3’
LPL	FW 5’ CCGCCGACCAAAGAAGAGAT 3’<br>RW 5’ TAGCCACGGACTCTGCTACT 3’
RUNX2	FW 5’ CCGGAATGCCTCTGCTGTTA 3’<br>RW 5’ TGTCTGTGCCTTCTGGGTTC 3’
RANKL	FW 5’ GACACAACTCTGGAGAGTCA 3’<br>RW 5’ TCTGCTCTGATGTGCTGTGATC 3’
OPN	FW 5’ TAGGCATCACCTGTGCCATA 3’<br>RW 5’ CTCAGAACTTCCAGAATCAGCC 3’
IL8	FW 5’ CACCGGAAGGAACCATCTCA 3’<br>RW 5’ TGGCAAAACTGCACCTTCACA 3’
TNF	FW 5’ CCTGCTGCACTTTGGAGTGA 3’<br>RW 5’ GAGGGTTTGCTACAACATGGG 3’
CCL2	FW 5’ AGAATCACCAGCAGCAAGTGTCC 3’<br>RW 5’ TCCTGAACCCACTTCTGCTTGG 3’
BAX	FW 5’ CCCCGAGAGGTCTTTTTCCG 3’<br>RW 5’ TGGTTCTGATCAGTTCCGGC 3’

CDKN1A	FW 5’ CAGCATGACAGATTTCTACCACTC 3’<br>RW 5’ CTCGCGCTTCCAGGACTG 3’
CDKN2A	FW 5’ CACCAGCGTGTCCAGGAAG 3’<br>RW 5’ CAACTGCGCCGACCCC 3’
CDKN2B	FW 5’ CAGACGACCCCAGGCATC 3’<br>RW 5’ CTGCCACTCTCACCCGAC 3’
BCLα	FW 5’ CTTTGAGTTCGGTGGGGTCA 3’<br>RW 5’ GGGCCGTACAGTTCCACAAA 3’
BCLβ	FW 5’ GGTGAACTGGGGGAGGATTG3’<br>RW 5’ GCCCAGACTCACATCACCAA 3’
ACTB	FW 5’ ACAGAGCCTCGCCTTTGCC 3’<br>RW 5’ GATATCATCATCCATGGTGAGCTGG 3’
GUSB	FW 5’ CTGACACCTCCAAGTATCCCAAG 3’<br>RW 5’ GTCGTGTACAGAAGTACAGACCGC 3’

Colony-Forming Units (CFUs) assays. Clonogenic capacity of HSPCs and GE HSPCs cultured *in vitro* was evaluated by CFUs assay using methylcellulose-based MethoCult (Stemcell H4434). 500 were plated in 1,1ml of MethoCult in 35mm dish in triplicates for each condition. The number and the type colonies was determined at day 7 or day 14 using the Zeiss Axio Observer inverted microscope.

Luminex assay. The presence of hematopoietic supportive factors and inflammatory cytokines was quantified (pg/ml) in the medium conditioned from BM-MSCs using the Immune Monitoring 65-Plex Human ProcartaPlex™ Panel (EPX650-10065-901) and normalized for the number of cells plated according to the kit assay protocol.

Gene editing of UCB CD34$^+$ HSPCs. UCB CD34$^+$ cells (purchased from Lonza) were seeded at a density of 5x10^5 cells/ml in serum-free StemSpan medium (StemCell Technologies) supplemented with penicillin, streptomycin, glutamine, 1 mM SR-1(Biovision), 50 nM UM171 (STEMCell Technologies), 10 mM PGE2 added only at the beginning of the culture (Cayman),

and human early-acting cytokines (SCF 100 ng/ml, Flt3-L 100 ng/ml, TPO 20 ng/ml, and IL-6 20 ng/ml; all purchased from Peprotech).

G-CSF mPB CD34+ HSPCs were purified with the CliniMACS CD34 Reagent System (Miltenyi Biotec) from Mobilized Leukopak (AllCells) upon approval by the Ospedale San Raffaele Bioethical Committee (TIGET-HPCT) according to manufacturer's instructions. HSPCs were seeded at the concentration of 5x10^5 cells/ml in serum-free StemSpan medium (StemCell Technologies) supplemented with 100 IU/ml penicillin, 100 μg/ml streptomycin, 2% glutamine, 300 ng/ml hSCF, 300 ng/ml hFlt3-L, 100 ng/ml hTPO and 10 μM PGE2 (at the beginning of the culture). Culture medium was also supplemented with 1 μM SR1 and 35 nM UM171. HSPCs were cultured in a 5% CO2 humidified atmosphere at 37C. After 3 days of pre-stimulation in early active cytokine medium, HSPCs were washed with PBS and electroporated using P3 Primary Cell 4D-Nucleofector X Kit and program EO-100 (Lonza). Cells were electroporated with 1,25 μM of RNPs. The following gRNA was used: *5' GTCACCAATCCTGTCCCTAGTGG 3'*. Transduction with AAV6 was performed at a dose of 1-2 x10^4 vg/cell 15' after electroporation. AAV6 donor templates were generated from a construct containing AAV2 inverted terminal repeats, produced at the TIGEM Vector Core by a triple-transfection method and purified by ultracentrifugation on a cesium chloride gradient. An AAV6 donor template carrying a PGK-GFP-polyA cassette flanked by homology arms for the AAVS1 locus was used for the *in vitro* experiments [2], while a barcoded version of the same vector was used in xenograft experiments to follow the clonal reconstitution dynamics of edited cells, as previously described [3]. In any case, gene editing efficiency was measured by flow cytometry as percentage of GFP+ cells in GE-HSPCs at 24 and 72 hours after electroporation. When indicated, in the absence of AAV6 donor template, HSPCs were gene knockout (KO) by NHEJ.

HDR and NHEJ molecular analysis. For molecular analysis of HDR and NHEJ, genomic DNA was isolated with QIAamp DNA Micro Kit (Qiagen) according to the manufacturer's instructions. HDR-mediated integration was quantified by ddPCR, using primers and probes designed on the junction between the vector sequence and the targeted locus and on control sequence used for normalization (human TTC5 gene) as previously published (2).

Nuclease activity was measured by mismatch-sensitive endonuclease assay by PCR-based amplification of the targeted locus followed by digestion with T7 Endonuclease I (NEB) according to the manufacturer's instructions. Digested DNA fragments were resolved and quantified by capillary electrophoresis on TapeStation (ThermoFisher) according to the manufacturer's instructions.

RNA sequencing. MSCs expanded in culture and MSCs co-cultured with GE-HSPCs for 72 hours were washed two times with PBS, incubated with trypsin and collected for RNA extraction. RNA was extracted using RNeasy Micro Kit (Qiagen, 74004), according to the manufacturer's instructions and DNase treatment was performed using RNase-free DNase Set (Qiagen,79254). RNA quality control was performed on HSRNA chip (Tape Station 2100, Agilent), and only samples with RIN>7 were processed. RNA library preparation was performed using the Illumina Ribo-Zero Plus rRNA Depletion Kit (Illumina) following the manufacturer's instruction. Briefly, starting from 100ng of total RNA, RNA libraries were barcoded, pooled, denatured and diluted to an 0.8 pM final concentration. The SBS (sequencing by synthesis) was performed onto Novaseq 6000 (Illumina) set to 100 cycles, yielding an average of 30-40x106 clusters for each sample in SR mode. Demultiplexing was performed using bcl2fastq. Quality check of RNA-seq sequences was performed using FastQC and low-quality reads were trimmed with Cutadapt. Alignment

against the human reference genome (GRCh38/hg38) was done using STAR, with standard input parameters, and gene counts were produced using Subread featureCounts and Genecode v.34 as gene annotation. Transcript counts were processed with the R/Bioconductor package edgeR, normalizing for library size using trimmed mean of M values and correcting p-values using FDR. Moreover, differential gene expression (DGE) analysis between MSCs and MSCs co-cultured with HSPCs treated for GE was done with the edgeR glmFit function (which fits a negative binomial generalized log-linear model for each gene and conducts genewise statistical tests), and differentially expressed genes (DEGs) were identified accordingly (FDR < 0.01 and |logFC| > 2). Gene Set Enrichment Analysis was performed by the R/Bioconductor package ClusterProfiler on pre-ranked (by logFC) gene lists against the Gene Ontology (GO) – Biological Processes (BP) database.

BAR-Seq amplicon library preparation, sequencing and bioinformatic analyses

Library preparation, purification and sequencing for BAR-Seq were performed as previously described [4]. Barcode sequences were extracted from input FASTQ files using the BAR-Seq bioinformatic pipeline, analyzed with default parameters (edit-distance = 3, min-count = 3, and saturation = 90), and quantified by computing logCPM value

Flow cytometry HSPC characterization. For immunophenotypic analyses (performed on FACSCanto II; BD PharMingen), we used CD90 APC (BD Biosciences, 559869). CD133 PECy7 (Miltenyi Biotec, 130-101-652), CD34 PE (Miltenyi Biotec, 130-081-002). Single stained and Fluorescence Minus One stained cells were used as controls. 7-AAD Viability Staining Solution (BioLegend) were included in the sample preparation for flow cytometry to exclude dead cells

from the analysis. Apoptosis analysis was performed by 7-AAD staining in combination with Annexin V (BioLegend) according to the manufacturer's instructions.

Cell cycle phases analysis by EdU/ Hoechst staining EdU (5-ethynyl-20 -deoxyuridine), supplied with Click-iT EdU Alexa Fluor 647 Imaging Kit (#C10424, Thermo-Fisher Scientific, Waltham, MA, USA), was diluted in DMSO to a final concentration of 10 mM and kept at -20°C. 0.5-1x105 cells were treated with 2mM EdU for 4 hours in culture. Cells (approx. 0,5-1 3 105 cells) were washed with 3 mL of 1% BSA in PBS and fixed with 100 mL of Click-iT fixative for 15 min. Cells were washed again with 3 mL of 1% BSA in PBS and permeabilized with 100 mL of 1X Click-iT saponin-based permeabilization for 15 min. Detection of EdU-DNA was performed by incubating cells with 500 mL of Click-iT Plus reaction cocktail for 30 min at RT protected from light. Cells were subsequently washed with 3 mL of 1% BSA in PBS before staining of DNA with Hoechst for 1h at RT protected from light and immediately after their fluorescence was measured by flow cytometry.

Immunofluorescence Analysis. Multitest slides (10 well, MP Biomedicals) were treated for 20' with Poly-L-lysine solution (Sigma-Aldrich) at 1mg/ml concentration. After two washes with DPBS solution, approximately 0.5/1x105 cells were seeded on covers for 20' and fixed with 4% paraformaldehyde (Santa Cruz Biotechnology) for other 20'. Cells were then permeabilized with 0.1% Triton X-100. After blocking with 0.5% BSA and 0.2% fish gelatin in DPBS, cells were probed with the indicated primary antibodies. After primary antibodies incubation (53BP1 Antibody, Bethyl Laboratories; Anti-phospho Histone H2A.X (Ser139) Antibody, clone JBW301, Merck), cells were washed three times with DPBS and incubate with Alexa 488-, 568- and/or 647-labeled secondary antibodies (Invitrogen). Nuclear DNA was stained with DAPI at 0.2 mg/ml

concentration (Sigma-Aldrich) and covers were mounted with Aqua-Poly/Mount solution (Polysciences. Inc.) on glass slides (Bio-Optica). Fluorescent images were acquired using Leica SP2 and Leica SP5 Confocal microscopes. Quantification of DDR foci in immunofluorescence images was conducted using Cell Profiler (version 2.1.1, revision 6c2d896).

Statistical analyses

Data were expressed as means ± SEM or dot plots with mean values indicated as a line. "n" indicates biologically independent samples/animals/experiments. Mann-Whitney test was performed to compare two independent groups. Analyses were performed using GraphPad Prism v8. Differences were considered statistically significant at $*p < 0.05$, $**p < 0.01$. "ns" indicates not significant comparison.

In our gene-editing experiments we used biological replicates of MSCs derived from healthy donors of the same age range, cultured using a standardized method and co-cultured with HSPCs at a similar *in vitro* passage (p3-p5). For each experiment, we co-cultured MSC biological replicates with a pool of N. 3 UCB-CD34$^+$ cells derived from different healthy donors.

Supplementary References

1. Crippa, S. *et al.* Bone marrow stromal cells from beta-thalassemia patients have impaired hematopoietic supportive capacity. *J Clin Invest* **129**, 1566-1580 (2019).
2. Schiroli, G. *et al.* Precise Gene Editing Preserves Hematopoietic Stem Cell Function following Transient p53-Mediated DNA Damage Response. *Cell Stem Cell* **24**, 551-565 e558 (2019).
3. Ferrari, S. *et al.* Efficient gene editing of human long-term hematopoietic stem cells validated by clonal tracking. *Nat Biotechnol* **38**, 1298-1308 (2020).
4. Ferrari, S. *et al.* BAR-Seq clonal tracking of gene-edited cells. *Nat Protoc* **16**, 2991-3025 (2021).
