# Supplementary material for: Mesenchymal stromal cells improve the transplantation outcome of CRISPR-Cas9 gene-edited human HSPCs
Source: Mol Ther. 2022 Aug 17;31(1):230–48. doi: 10.1016/j.ymthe.2022.08.011 (PMC9840125; doi:10.1016/j.ymthe.2022.08.011)
Supplement: Document S2. Article plus supplemental information [file mmc4.pdf]

Molecular Therapy
Original Article

Mesenchymal stromal cells improve the transplantation outcome of CRISPR-Cas9 gene-edited human HSPCs

Stefania Crippa,[1,8] Anastasia Conti,[1,8] Valentina Vavassori,[1] Samuele Ferrari,[1] Stefano Beretta,[1] Silvia Rivis,[2] Roberto Bosotti,[1] Serena Scala,[1] Stefania Pirroni,[3] Raisa Jofra-Hernandez,[1] Ludovica Santi,[1] Luca Basso-Ricci,[1] Ivan Merelli,[1,4] Pietro Genovese,[1,5] Alessandro Aiuti,[1,6,7] Luigi Naldini,[1,7] Raffaella Di Micco,[1] and Maria Ester Bernardo[1,6,7]

[1]San Raffaele Telethon Institute for Gene Therapy (SR-TIGET), IRCCS San Raffaele Scientific Institute, 20132 Milan, Italy; [2]Laboratory of Tumor Inflammation and Angiogenesis, VIB-KULeuven, 3000 Leuven, Belgium; [3]Cagliari University-Hospital, 09100 Cagliari, Italy; [4]National Research Council, Institute for Biomedical Technologies, 20132 Milan, Italy; [5]Dana-Farber/Boston Children's Cancer and Blood Disorders Center, Department of Pediatric Oncology, Harvard Medical School, Boston, MA 02115, USA; [6]Pediatric Immunohematology and Bone Marrow Transplantation Unit, San Raffaele Scientific Institute, 20132 Milan, Italy; [7]"Vita Salute" San Raffaele University, 20132 Milan, Italy

Mesenchymal stromal cells (MSCs) have been employed *in vitro* to support hematopoietic stem and progenitor cell (HSPC) expansion and *in vivo* to promote HSPC engraftment. Based on these studies, we developed an MSC-based co-culture system to optimize the transplantation outcome of clustered regularly interspaced short palindromic repeats (CRISPR)-Cas9 gene-edited (GE) human HSPCs. We show that bone marrow (BM)-MSCs produce several hematopoietic supportive and anti-inflammatory factors capable of alleviating the proliferation arrest and mitigating the apoptotic and inflammatory programs activated in GE-HSPCs, improving their expansion and clonogenic potential *in vitro*. The use of BM-MSCs resulted in superior human engraftment and increased clonal output of GE-HSPCs contributing to the early phase of hematological reconstitution in the peripheral blood of transplanted mice. In conclusion, our work poses the biological bases for a novel clinical use of BM-MSCs to promote engraftment of GE-HSPCs and improve their transplantation outcome.

INTRODUCTION

Mesenchymal stromal cells (MSCs) are crucial elements in the bone marrow (BM) niche, where they are physically associated with hematopoietic stem and progenitor cells (HSPCs) and regulate their homeostasis mainly through the release of paracrine factors.[1–3]

Ex vivo expanded human MSCs[4] have been employed in pre-clinical models of HSC transplantation (HSCT) and phase I/II clinical trials to favor the engraftment of transplanted HSPCs and their hematopoietic reconstitution.[5–9] Despite transplanted MSCs not persisting long term,[10] previous works indicate that the MSC secretion of anti-inflammatory molecules reduces the inflammatory response to pre-transplant conditioning, rendering the BM niche a more favorable environment for the engraftment of transplanted HSPCs.[11–13]

In addition to their *in vivo* use, MSCs were extensively studied to enhance the *ex vivo* expansion of umbilical cord blood (UCB)-derived HSPCs before HSCT. MSCs improve the outcome of UCB-HSPC transplantation due to their ability to secrete hematopoietic supportive factors[14–16] and directly interact with HSPCs to promote survival and proliferation while preventing their differentiation.[17] Moreover, several works indicated that the use of stromal cells in co-culture with HSPCs also improves the gene-transfer efficiency in long-term repopulating HSPCs by producing a supportive stromal matrix and releasing stromal factors.[18–20]

Autologous HSPC gene therapy (GT) with corrective transgenes delivered by retro/lentiviral vectors has recently become a curative treatment for different inherited genetic disorders.[21–25] Meanwhile, this rapidly evolving field is already witnessing the development of a new generation of advanced genetic therapies based on gene editing (GE) technologies, which employ programmable nucleases, such as clustered regularly interspaced short palindromic repeats (CRISPR)-Cas9, to achieve locus-specific gene correction, thus minimizing the risk of genome-wide vector integration.[26] However, the efficiency of GE strategies based on homology-directed repair (HDR) remains limited in long-term (LT) repopulating HSPCs due to low proficiency of homologous recombination, limited permissiveness

Received 25 February 2022; accepted 12 August 2022;
https://doi.org/10.1016/j.ymthe.2022.08.011.

[8]These authors contributed equally

Correspondence: Stefania Crippa, PhD, San Raffaele Telethon Institute for Gene Therapy (SR-TIGET), IRCCS San Raffaele Scientific Institute, 20132 Milan, Italy.
E-mail: crippa.stefania@hsr.it
Correspondence: Maria Ester Bernardo, MD, PhD, San Raffaele Telethon Institute for Gene Therapy (SR-TIGET), IRCCS San Raffaele Scientific Institute, 20132 Milan, Italy.
E-mail: bernardo.mariaester@hsr.it

 © 2022 The Authors.
This is an open access article under the CC BY license (http://creativecommons.org/licenses/by/4.0/).

to the delivery of the DNA-repair template,[27–34] and the induction of cellular responses hampering the proliferative capacity of edited cells.[35] These constrains lead to a restricted yield of edited HSPCs and limit the number of LT repopulating HSPCs edited by HDR available for transplantation, which could cause delayed engraftment and increased risk of graft failure in patients.

Based on these premises, the identification of novel and effective strategies to further improve the *ex vivo* maintenance/expansion and/or the *in vivo* engraftment of GE-HSPCs is a critical requirement for successful clinical translation of GE.

Previous work demonstrated that the transient inhibition of p53 increases the yield of clonogenic and repopulating GE-HSPCs.[35] However, a multifactorial approach would better control the distinct signals activated in HSPCs upon nuclease-induced double-strand breaks (DSBs), known to induce a protracted DNA-damage response (DDR) activation and proliferation delay.

For this reason, we performed CRISPR-Cas9 GE in human HSPCs, and we employed an MSC-based two-dimensional (2D) co-culture system to increase the number and the fitness of gene-edited HSPCs available for transplantation. We based our strategy on the critical role of stromal cells in supporting cell expansion and gene transfer into primitive HSPCs and considering their anti-inflammatory properties mediated by the secretion of multiple paracrine factors.[14–16,18,19] In this study, we investigate for the first time the supportive activity of MSCs on GE HSPCs, demonstrating that MSCs attenuate the proliferation block and the inflammatory response associated with the GE procedure in HSPCs, resulting in an improved transplantation outcome of GE-HSPCs.

The results of our work provide the biological basis for the clinical use of MSCs in HSPC-based GE applications.

RESULTS

Characterization of the hematopoietic supportive capacity of human MSCs

Human MSCs were isolated according to standard protocols[36] from pediatric sibling donors undergoing BM harvest (median age: 12 years, range: 4–18 years) after parental informed consent. BM-MSCs were *ex vivo* expanded and characterized in terms of clonogenic capacity, proliferation, expression of MSC markers, and differentiation potential according to the minimal criteria defined by the International Society for Cellular Therapy[4] (Figures S1A–S1F).

In detail, all the MSC samples used in this study were capable of forming colony-forming unit fibroblasts (CFU-Fs) (Figure S1A), acquired a spindle-like morphology (Figure S1B), and efficiently proliferated between passage 3 to 6 *in vitro* (Figure S1C). All samples expressed the canonical MSC surface markers (CD90, CD73, CD105) and lacked the expression of hematopoietic (CD45, CD34, CD14), endothelial (CD31), and HLA class II (HLA-DR) markers (Figure S1D). As expected, they differentiated *in vitro* into adipocytes, osteoblasts, and chondrocytes (Figures S1E and S1F).

Importantly, all *ex vivo* expanded MSC samples show a higher expression than human fibroblasts of several transcripts encoding for hematopoietic supportive factors known to sustain and regulate the fate of human HSPCs in the BM niche (Figure S1G), some of which were also detected in the MSC-conditioned medium (Figure S1H). MSCs also released several anti-inflammatory molecules, as shown in Figure S1I.

Based on these results, we set the MSC-based co-culture conditions to exploit the hematopoietic supportive activity of *ex vivo* expanded human MSCs. In detail, MSCs were expanded in their proper medium for 2 days and exposed to HSPC culture medium for 24 h to enrich it with MSC-derived hematopoietic supportive factors. Commercially available human UCB-CD34$^+$ cells (hCD34$^+$) were co-cultured on an MSC feeder for 72 h in the previously obtained MSC-conditioned HSPC culture medium supplemented with the proper early-acting cytokines (+cytokines)[31] (Figure S2A). At the end of the co-culture, we determined the total number of hCD34$^+$ cells and evaluated their phenotypic composition and clonogenic capacity (Figures S2B–S2E). To this aim, we applied a novel flow cytometer gating strategy to prospectively identify the most primitive HSPC subpopulation as CD45$^+$, Lin$^-$, CD34^{high}, CD90$^+$, CD45RA$^-$ cells (Figure S3A).[37–42] Human HSPCs proliferated in culture in the presence of cytokines, reaching a significantly higher number of cells ($p = 0.0079$) when co-cultured with MSCs (Figure S2B). Phenotypic analysis resulted in a significantly higher number of HSPCs with a primitive phenotype ($p = 0.02$) (Figures S2C and S2D) characterized by an increased clonogenic capacity ($p = 0.02$) (Figure S2E) when cultured in the presence of MSCs compared with controls.

Overall, these data confirm that BM-MSCs promoted survival and maintenance of HSPCs with a primitive phenotype, and this effect was also evident in stress conditions, such as in the absence of supplementation with early-acting cytokines (−cytokines) (Figures S2B–S2E).

MSCs increase the number of GE-HSPCs available for HSCT

One of the main limitations to the clinical employment of HDR-edited HSPCs is the limited number of gene-corrected repopulating cells available due to the strong activation of a p53-mediated DDR elicited by the converging effect of nuclease-induced DNA DSBs and the cellular sensing of the adeno-associated viral vector serotype 6 (AAV6) used as source for DNA donor template delivery. As a consequence, exacerbated DDR constrains the proliferative and LT repopulating capacity of edited HSPCs.[35]

We reasoned that the BM-MSC supporting feeder could counteract GE-induced DDR and cell-cycle arrest, thus favoring the expansion of GE-HSPCs. To this purpose, human CD34$^+$ cells were gene edited with a CRISPR-Cas9 ribonucleoprotein (RNP) targeting the *AAVS1* safe harbor locus and transduced with a cognate AAV6 donor DNA suitable for HDR and encoding for GFP under the transcriptional control of the phosphoglycerate kinase (PGK) promoter,[35,43]

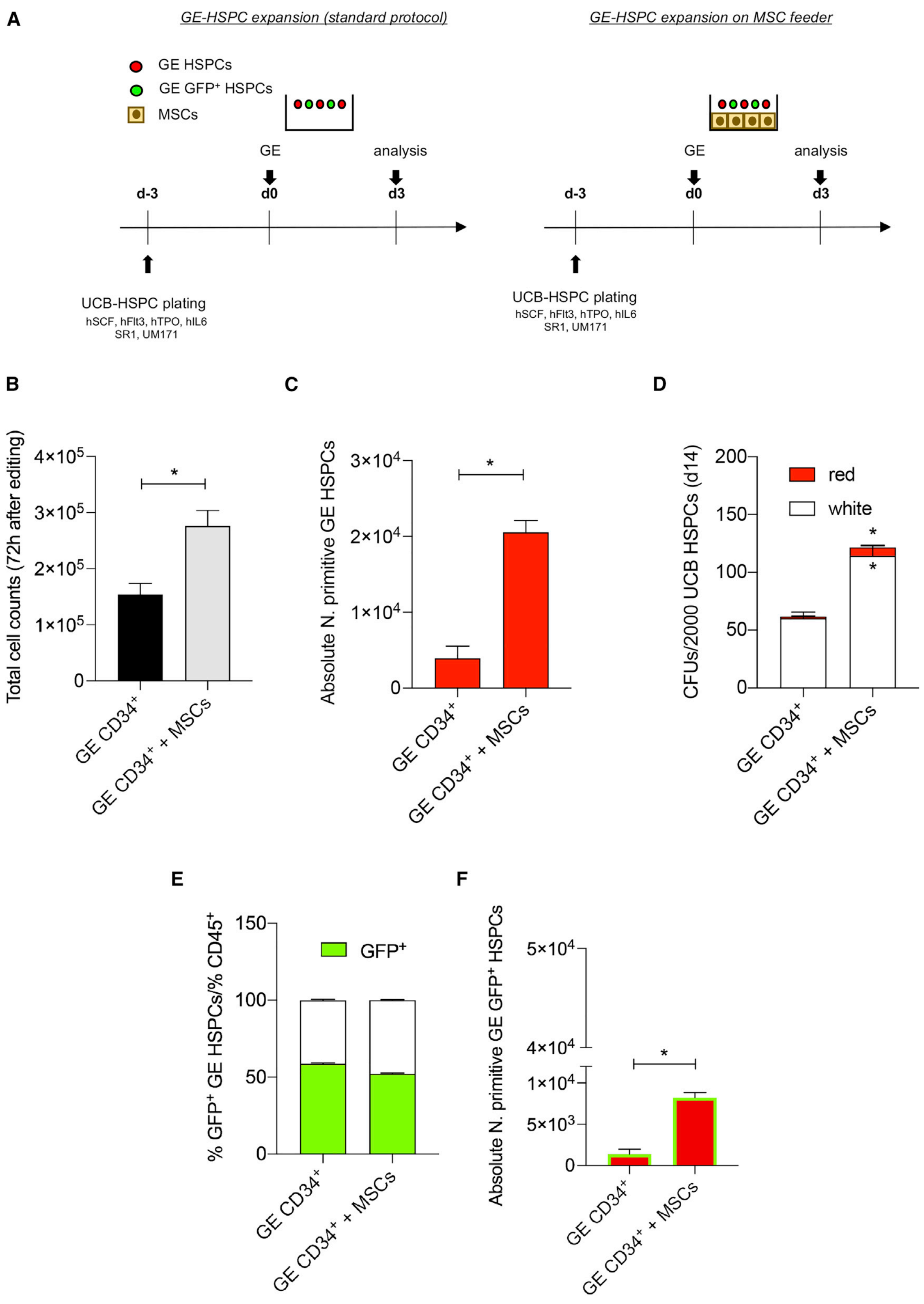

(legend on next page)

further referred as standard protocol. Cells treated for GE were expanded on MSCs for 72 h, as represented in Figure 1A and described in the materials and methods. Total count, phenotype, and absolute number of HSPCs were evaluated to assess the hematopoietic supportive capacity of MSCs on HSPCs treated for GE (Figures 1B–1F).

The number of GE-HSPCs co-cultured with MSCs was twice the number of control cells (p = 0.05) (Figure 1B). Importantly, MSCs favored the maintenance of the GE-HSPC subset with a primitive phenotype as shown by flow cytometry analysis (Figure S3B) and absolute counts of $CD45^+$, Lin^-, $CD34^{high}$, $CD90^+$, $CD45RA^-$ GE-HSPCs (Figure 1C), which were significantly higher (p = 0.02) in the presence of MSCs. Moreover, GE-HSPCs formed a higher number of colonies (white colonies: p = 0.02; red colonies: p = 0.05), confirming an enrichment of highly clonogenic cells in GE-HSPCs co-cultured with MSCs (Figure 1D). Importantly, while we observed that MSCs did not alter the efficiency of DSB repair via HDR (Figure 1E), when dissecting the phenotype of GFP^+ GE-HSPCs, we found a higher number of GFP^+ GE-HSPCs with a primitive phenotype ($CD45^+$, Lin^-, $CD34^{high}$, $CD90^+$, $CD45RA^-$) after MSC co-culture (p = 0.02) (Figure 1F).

Altogether, these results indicate that the *ex vivo* co-culture of HSPCs treated for GE with BM-MSCs represents a suitable strategy to expand GE-HSPCs while preserving the HSPC subsets with a primitive phenotype, including HSCs (CD90+, CD45RA−), multimyeloid progenitors, MMPs ($CD90^-$, $CD45RA^-$), and multilymphoid progenitors (MLPs; $CD90^-$, $CD45RA^+$) available for transplantation.

Despite the protective effects of MSCs, we noted that the 72 h culture of GE-HSCPs significantly reduced the frequency of $CD34^+$ cells with a primitive phenotype compared with the 24 h recovery time, likely due to the culture-induced HPSC activation (Figure S3C). These results (Figures 1, S2, S3B, and S3C) correlate with the time of GE-HSPC transplantation: indeed, HSPCs are usually *ex vivo* cultured for 72 h prior to genetic engineering and infused 24 h after GE in pre-clinical mouse models of HSCT.

Based on this, we reasoned to exploit the hematopoietic supportive activity of MSCs in both the stimulation (3 day expansion before GE) and recovery phase post-GE of our GE protocol (Figure 2A).

We analyzed the total cell count, phenotype, and absolute number of HSPCs after 72 h of pre-stimulation, at 24 h recovery, and 72 h expansion post-editing. For all time points, we found a significantly higher (pre-stimulation: p = 0.05, 24 h recovery: p = 0.05; 72 h expansion: p = 0.03) number of $CD34^+$ in the presence of MSCs compared with controls (Figures 2B and S3D). We confirmed that the co-culture with MSCs favored the expansion of cells treated for GE, and we also observed a diminished loss of Lin^-, $CD34^{high}$, $CD90^+$, $CD45RA^-$ HSPCs when $CD34^+$ cells were pre-stimulated in co-culture with MSCs (Figures 2C, S3D, and S3E). Indeed, we found that MSCs prevented the reduction of phenotypically primitive HSPCs at 24 h post-editing and at longer time points, despite the additional effect of culture-induced HSPC differentiation (Figures 2C and S3F).

Importantly, we found that the efficiency of gene correction was not affected by the presence of MSCs in co-culture with HSPCs (Figure 2D). We also observed a significantly higher number of HSPCs gene edited by HDR (GFP^+) with a primitive phenotype in the presence of MSCs (Figure 2E). The combined use of MSCs to expand HSPCs before GE and to recover HSPCs after GE procedures confirmed that the presence of MSCs enriched the total number of GFP^+ GE-HSPCs with a primitive phenotype (Figure 2E). We also showed a similar percentage of HDR and NHEJ at 72 h post-editing in cells gene edited in the presence of MSCs compared with our standard controls (Figure S3G).

Considering the fundamental role of the vascular niche in the control of HSPC homeostasis and the functional interaction of endothelial cells with MSCs,[2] we aimed at further improving the hematopoietic support of our 2D co-culture system by using a mixed feeder of human umbilical vein endothelial cells (HUVECs) and MSCs. We observed that the total cell counts and the absolute number of phenotypically primitive HSPCs tended to increase in the presence of a mixed feeder (MSCs:HUVECs, 10:1) compared with standard protocol and the use of MSCs alone (Figures S3H and S3I). However, when we translated this approach to GE-HSPCs, HUVECs died in the presence of UM171 and SR1, which are important to support maintenance and expansion of GE HSPCs in culture (Figure S3J).

In conclusion, we exploited the use of MSCs alone to increase the number of gene-targeted cells available for transplantation and to preserve their primitive phenotype.

HSPCs gene edited in the presence of MSCs showed a superior engraftment capacity

We next investigated whether the increased absolute number of HSPCs obtained using BM-MSCs as a feeder during the GE

Figure 1. MSCs favored the expansion of GE-HSPCs

(A) Schematic representation of the co-culture protocol used to support the expansion of GE-HSPCs. HSPCs were pre-stimulated for 3 days in HSPC GE medium (hSCF, hFLT3, hTPO, hIL-6, SR1, UM171) and recovered in HSPC medium conditioned from MSCs in the presence of MSCs for 72 h. GE-HSPCs recovered in HSPC medium on plastic according to our standard method were used as controls. (B) GE-HSPC total cell count after 72 h expansion. Gray bars: GE-HSPCs expanded on MSC feeder; black bars: GE-HSPCs expanded in plastic dishes. (C) Absolute number of GE-HSPCs with a primitive phenotype was determined after 72 h expansion. Each error bar shows means ± SEM (n = 4). (D) CFU assay at 7 days after plating GE-HSPCs into methylcelluose medium. (E) Percentage of GFP^+ HSPCs 72 h after gene editing in the presence of MSCs (GE $CD34^+$ + MSCs) and according to standard protocol (GE $CD34^+$) (n = 4). (F) Absolute number of phenotypically primitive GFP^+ GE-HSPCs after 72 h expansion. For all experiments, each error bar shows means ± SEM (n = 4). p values were determined by Mann-Whitney test (*$p \leq 0.05$).

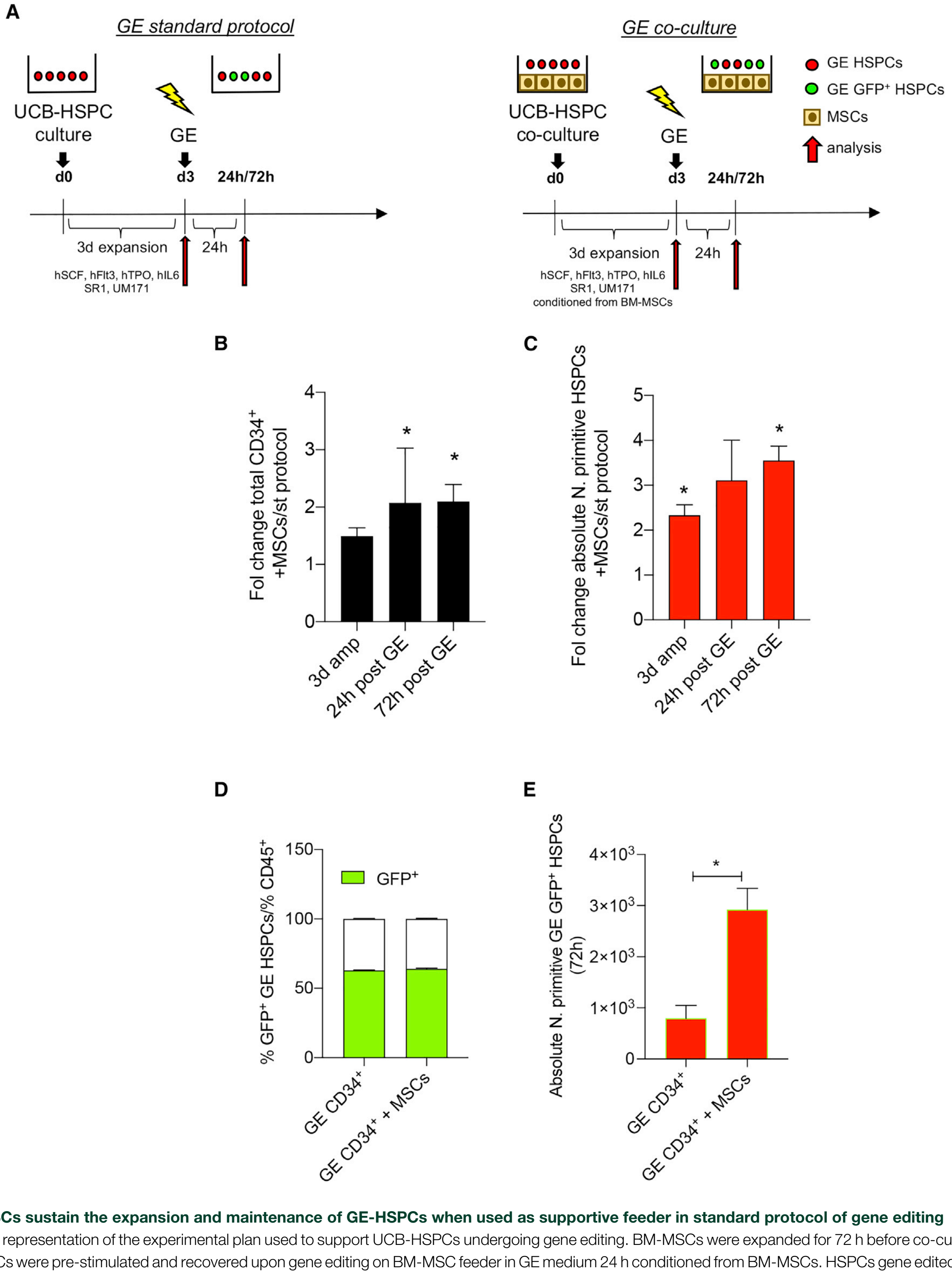

Figure 2. MSCs sustain the expansion and maintenance of GE-HSPCs when used as supportive feeder in standard protocol of gene editing

(A) Schematic representation of the experimental plan used to support UCB-HSPCs undergoing gene editing. BM-MSCs were expanded for 72 h before co-culture with GE-HSPCs. HSPCs were pre-stimulated and recovered upon gene editing on BM-MSC feeder in GE medium 24 h conditioned from BM-MSCs. HSPCs gene edited according to our standard protocol were used as controls. (B and C) Fold change analysis of CD34+ total cell counts (B) and absolute number of HSPCs with a primitive phenotype (C) after 3 days of expansion before editing (3d amp) and after 24 and 72 h recovery after editing in the presence of MSCs compared with standard protocol. (D) Percentage of GFP^+ GE-HSPCs cells after 72 h expansion on MSC feeder (GE $CD34^+$ + MSCs) or in plastic dishes (GE $CD34^+$). (E) Absolute number of HDR gene-edited (GFP^+) GE-HSPCs after co-cultured with MSCs (GE $CD34^+$ + MSCs) or expanded in plastic dishes (GE $CD34^+$) according to standard protocol. For all the experiments, each error bar shows means ± SEM ($n \geq 3$). p values were determined by Mann-Whitney test (*$p \leq 0.05$).

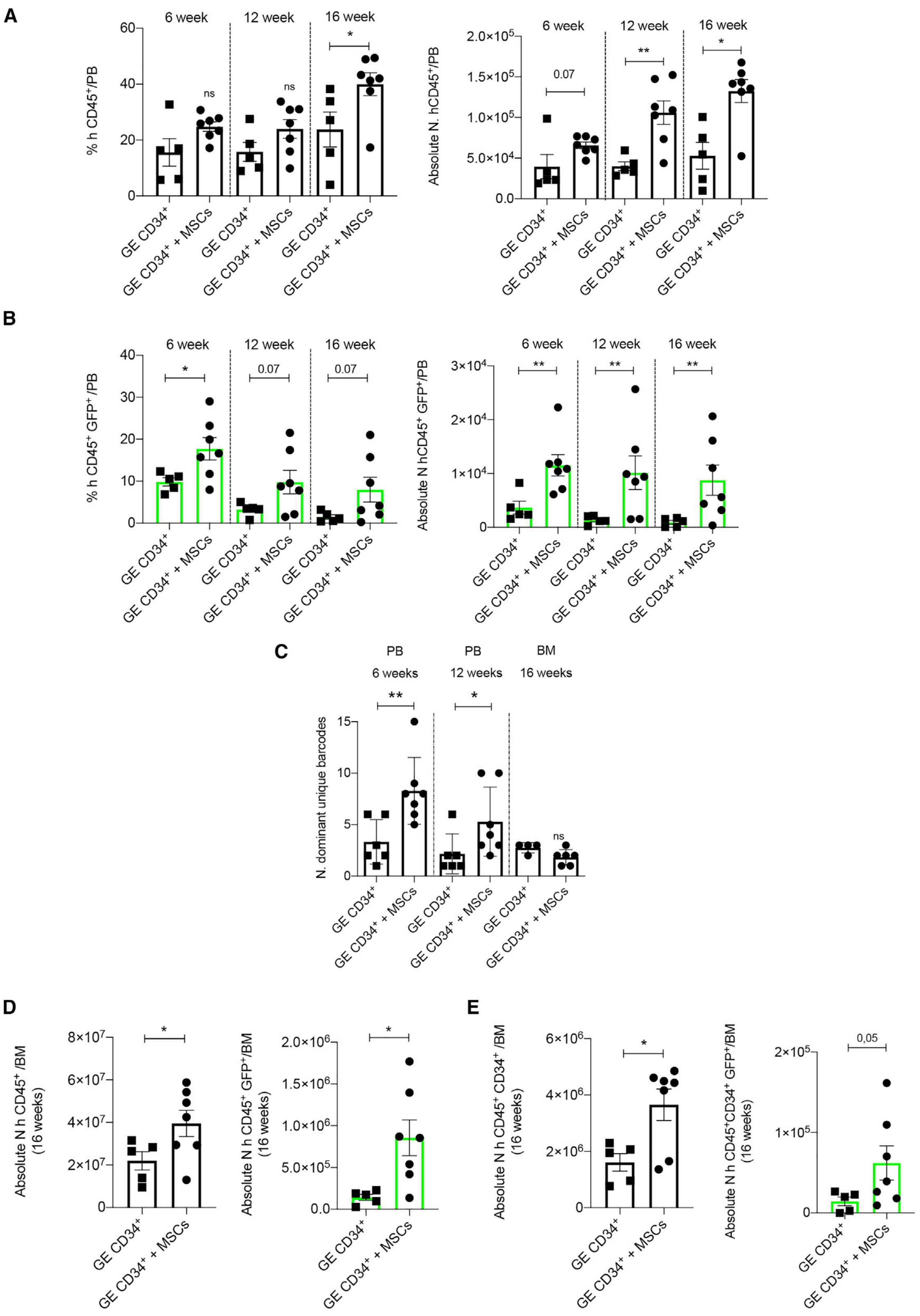

(legend on next page)

procedures correlated with an improved transplantation efficacy. To assess the clonal composition and dynamics of edited cells in the human xenograft, we performed GE in the same locus but using an AAV6 also embedding unique molecular barcodes to individually track HDR-edited cell clones by barcode analysis by sequencing (BAR-seq) technology.[44,45]

We transplanted the outgrown cells of starting matched doses (1×10^5) of UCB-derived GE-HSPCs co-cultured with MSCs or standard GE-HSPCs, as control, into sublethally irradiated immunodeficient non-obese diabetic (NOD)-severe combined immunodeficiency (SCID)-IL2Rg$^{-/-}$ (NSG) mice. Human cell engraftment and hematological reconstitution were evaluated in the peripheral blood (PB) of transplanted mice at different time points and in the BM at the end of the experiment (16 weeks) (Figure S4A).

We showed an increased human engraftment, which was statistically significant ($p = 0.004$) at 12 and 16 weeks, in the PB of mice transplanted with GE-HSPCs + MSCs (Figure 3A), indicating that *ex vivo* co-culture with MSCs favored durable human CD45$^+$ cell engraftment. In these mice, we also found an increased number of HDR-edited (GFP$^+$) cells, which was maintained over time post-transplantation (Figure 3B).

Moreover, BAR-seq analysis showed an increase in repopulating HDR-edited clones,[44,45] as supported by the higher number of dominant unique barcodes retrieved from PB of the mice, at 6 and 12 weeks after transplantation of HSPCs gene edited in the presence of MSCs (Figures 3C and S5A). On the contrary, the number of gene-edited human CD45$^+$ cells decreased overtime in the PB of mice transplanted with standard GE-HSPCs, and overall fewer clones participated in the hematological reconstitution, as expected by the kinetics of hematopoietic reconstitution and also as previously described (Figures 3C and S5A).[39,44,46] Despite a trend for decreased engraftment of human GFP$^+$ cells in the PB at a later time point (16 week), the number of mice with a detectable level of GFP$^+$ (>0.1%) was higher in the GE-HSPC + MSC group (Figure 3B) compared with mice transplanted according to our standard procedures.

In addition, the absolute number of human B cells (CD19$^+$), T lymphocytes (CD3$^+$), and myeloid cells (CD13$^+$CD33$^+$) was significantly higher in the PB of mice transplanted with GE-HSPCs + MSCs (Figures S4B–S4D). At the endpoint (16 weeks), we observed a trend for a higher level of human engraftment (Figure 3D, left panel) and a significant increase in the absolute number of hCD45$^+$CD34$^+$ cells (Figure 3E, left panel) in the BM of mice transplanted with GE-HSPCs cultured in the presence of MSCs. In these mice, the absolute number of human cells gene edited by HDR (GFP$^+$) engrafted in the BM was significantly higher ($p = 0.02$) (Figure 3D, right panel) and showed a similar clonal reconstitution capacity compared with standard GE procedures (Figures 3C and S5A).

In conclusion, our results suggest that MSCs favored the expansion *in vitro* of early hematopoietic progenitors contributing to the improved early-phase engraftment and graft clonality while preserving the HSC compartment (CD34^{high}CD90$^+$CD45RA$^-$) (Figure S5A). This correlated with the higher number of transplanted cells when co-cultured with MSCs. In this specific case, we confirmed that MSCs favored HSPC expansion (fold change on starting matched dose: 2.28 + MSCs; 1.7 standard protocol), reduced post-GE detrimental cellular response (percentage of cell death: + MSCs: 10%; standard protocol: 22.5%), and reduced the loss of phenotypically primitive GE-HSPCs, resulting in a higher absolute number of transplanted primitive cells (+MSCs: 1,114; standard protocol: 280). We calculated the number of effectively transplanted cells per mouse and observed an averaged 1.7-fold change difference between GE-HSPCs + MSCs and GE-HSPCs (Figure S4E). We determined the ratio between the absolute counts of hCD45$^+$ cells in the PB and the number of effectively transplanted cells in both the transplantation conditions, and we calculated the fold change enrichment, which reflects the higher number of outgrown cells of starting matched doses co-cultured with MSCs (6 weeks: 1; 12 weeks: 1.53; 16 weeks: 1.45) (Figure S4F). We also determined the absolute counts of CD45$^+$CD34$^+$GFP cells in the BM of transplanted mice (+MSCs: 62,058; standard protocol: 14,378), and we normalized for the average number of transplanted outgrowing cells. Based on this calculation, we found an enrichment of a 2.5-fold change of gene-edited cells in the BM of mice transplanted with cells treated for GE in the presence of MSCs (Figure S4G), which is higher than the difference of effectively transplanted cells (1.7-fold), suggesting a specific supportive function of MSCs in sustaining GE cells.

MSCs mitigate DDR-induced cell-cycle arrest in GE-HSPCs

Recently, some of us showed that the robust activation of the p53-mediated DDR pathway limits the proliferation of GE-HSPCs and

Figure 3. Improved HSCT outcome after transplantation of HSPCs gene edited in the presence of MSCs into a pre-clinical xenograft model

(A) Flow cytometry analysis of human hematopoietic cell engraftment represented as percentage of hCD45$^+$ cells on total live cells based on physical parameters in the peripheral blood (PB) of mice transplanted with GE-HSPCs co-cultured with BM-MSCs (rounded points) or according to standard protocol (squared points) at different time points after transplantation (left panel). The absolute number of hCD45$^+$ cells was determined by flow cytometry adding known amount of Count Beads to the blood sample and using the counting equation according to the manufacturer protocol of absolute counting beads for flow cytometry (right panel). (B) Engraftment analysis of HDR gene-edited (GFP$^+$) human CD45$^+$ cells on total live cells based on physical parameters in the PB of transplanted mice at different time points after cell infusion (left panel). The absolute number of hCD45$^+$ cells was determined by flow cytometry using absolute counting beads (right panel). (C) Number of unique dominant barcodes identified in the PB (6 and 12 week time points) and in the BM (16 week time point) of mice transplanted with GE-HSPCs + MSCs (GE CD34$^+$ + MSCs) or standard GE-HSPCs (GE CD34$^+$) at different time points after cell infusion. (D) Absolute number of human CD45$^+$ cells (left panel) and human HDR gene-edited (GFP$^+$) CD45$^+$ cells (right panel) on total live cells based on physical parameters engrafted in the BM of transplanted mice at sacrifice. (E) Absolute number of human CD34$^+$ (left panel) and HDR gene-edited (GFP$^+$) CD34$^+$ (right panel) cells within the human CD45$^+$ engraftment on total live cells in the bone marrow at sacrifice (16 weeks). For all plots, individual data points represent an animal. p values were determined by Mann-Whitney test (*$p \leq 0.05$; **$p \leq 0.001$).

their LT repopulating capacity upon transplantation into immunocompromised mice.[35]

To further dissect the molecular mechanisms through which BM-MSCs promote the expansion and maintenance of HSPCs treated for GE, we analyzed the extent of DDR activation in GE-HSPCs co-cultured with MSCs at 24 and 72 h after GE (GE $CD34^+$ + MSCs). HSPCs gene edited according to our standard procedures were analyzed as control (GE $CD34^+$).

We first measured the subnuclear accumulation of 53BP1 and γH2AX foci in GE-HSPCs,[35] as surrogate markers of both nuclease-induced DSB (1–2 foci/nucleus) and culture-induced stress (>2 foci/nucleus) (Figure 4A). Twenty-four h after editing, we observed a higher number of 53BP1- and γH2AX-positive HSPCs compared with untreated (UT) samples and comparable levels of DDR foci when HSPCs were edited in the presence or absence of MSCs (Figures 4B and 4C). However, at later time points (72 h), we found a lower percentage of cells positive for 53BP1-γH2AX foci and a faster DDR resolution in GE-HSPCs co-cultured with MSCs. While the percentage of cells with one or two DDR foci per nucleus was similar in HSPCs gene edited in the presence or absence of BM-MSCs (Figure 4D), we found that the fraction of cells displaying more than 2 DDR foci, likely reflecting the DNA replication stress induced by their excessive proliferation in culture, was reduced in GE-HSPCs + MSCs, and this reduction was more evident in GE-HSPCs than in UT samples (Figure 4D).

Altogether, these data suggest that MSCs may favor the recovery of GE-HSPCs by both accelerating DDR foci resolution over time and protecting HSPCs from culture-induced stress or possibly by facilitating the growth of healthier cells over cells displaying a higher DDR burden.

We next measured the expression levels of *CDKN1A* (*p21*), a cell-cycle inhibitor downstream of the p53-DDR activation cascade, which was upregulated in GE-HSPCs compared with UT controls Figure 4E), in line with the induction of a stronger DDR in GE-HSPCs.[35] The upregulation of *p21* was mitigated in GE-HSPCs after 24 h of co-culture with MSCs (Figure 4E), indicating that MSCs may protect GE-HSPCs from p53-mediated cell-cycle arrest and apoptosis. Of note, UT HSPCs co-cultured with MSCs displayed higher levels of *p21*, possibly reflecting a feedback mechanism to control MSC-induced excessive proliferation in control cells.[47]

We also analyzed the impact of GE procedures on cell-cycle distribution and observed a decreased percentage of primitive and progenitor GE-HSPCs arrested in the G1 phase with a concomitant increase in cells in the S phase of the cell cycle 72 h post-GE in the presence of a supportive layer of MSCs (Figure 4F). In the same condition, we also detected a decrease in the percentage of cells in the G2/M phase in the more committed progenitor fraction (Figure S6A). No differences in cell-cycle distribution were observed in UT samples co-cultured with MSCs (Figure S6B).

Moreover, the presence of BM-MSCs reduced the apoptotic rate in the GE-HSPC progenitor subsets and the percentage of pan nuclear-stained γH2AX-positive cells (a surrogate marker for apoptosis) while increasing the expression of two key pro-survival factors, *BCL2α* and *BCL2β*, 72 h after editing (Figures 4G and S6C–S6G). For some of these molecular readouts, we also report a beneficial anti-apoptotic effect of MSCs on UT cells (Figures S6C–S6G).

We further investigated the expression of inflammatory cytokines in GE-HSPCs, considering the capacity of MSCs to sense and regulate the inflammation state of surrounding cells.[48] We found that the expression of CXC chemokine ligand 8 (*CXCL8*; hereafter named *IL-8*) and monocyte chemoattractant protein 1 (*MCP-1*; also known as *CCL2*), two pro-inflammatory cytokines activated in response to stress,[49,50] were induced by the GE procedure compared with UT cells, and their levels were reduced in GE-HSPCs cultured in the presence of an MSC feeder (Figures S6H and S6I), whereas the expression of another pro-inflammatory molecule, tumor necrosis factor alpha (*TNF-α*), did not change in the presence of MSCs (Figure S6J). An early upregulation of *IL-8* was observed when UT cells were co-cultured with MSCs (Figure S6H).

To determine whether the capacity of MSCs to mediate the repair proficiency and attenuate the inflammatory programs in GE-HSPCs was maintained over time, we replated GE-HSPCs previously cultured with or without MSCs into a medium with a low concentration of selected cytokines (20 ng/mL hTPO; 100 ng/mL hSCF; 1 μM SR1; 50 nM UM171) and in the absence of MSCs. In these conditions, we found a significant downregulation of *p21* and *CCL2* expression, as well as a trend toward decreasing *IL8* in GE-HSPCs previously co-cultured with MSCs, compared with control. Consistent with previous observations, *TNF-α* expression was still maintained similar to standard protocol GE-HSPCs (Figures S6K and S6L).

This suggested that MSCs mitigated the inflammatory signaling cascade in HSPCs treated for GE, keeping DDR activation under control, possibly due to their capacity to activate an anti-inflammatory program in response to inflammatory signals.[48]

We next performed a Luminex Multiplex assay on the supernatant of GE-HSPCs co-cultured with MSCs for 72 h after GE to identify possible paracrine factors supporting GE-HSPCs (Figure S7). In particular, we focused on those factors that could eventually protect HSPCs from the detrimental effects of DDR activation upon GE, including cell-cycle arrest and the activation of an inflammatory or apoptotic cascade.

We found that MSC-conditioned medium in the absence of additional cytokines contained significantly higher levels of hematopoietic supportive factors, including IL-2, IL-6, SDF1α, and VEGFA, compared with standard medium[51–54] (Figure S7A). We also observed an increase of FGF-2 and HGF, which could contribute to preventing the cell-cycle arrest in HSPCs after the GE procedure[55,56] (Figure S7B). Furthermore, we reported increased levels of LIF, which is known to regulate the proliferation of primitive HSPCs[57]

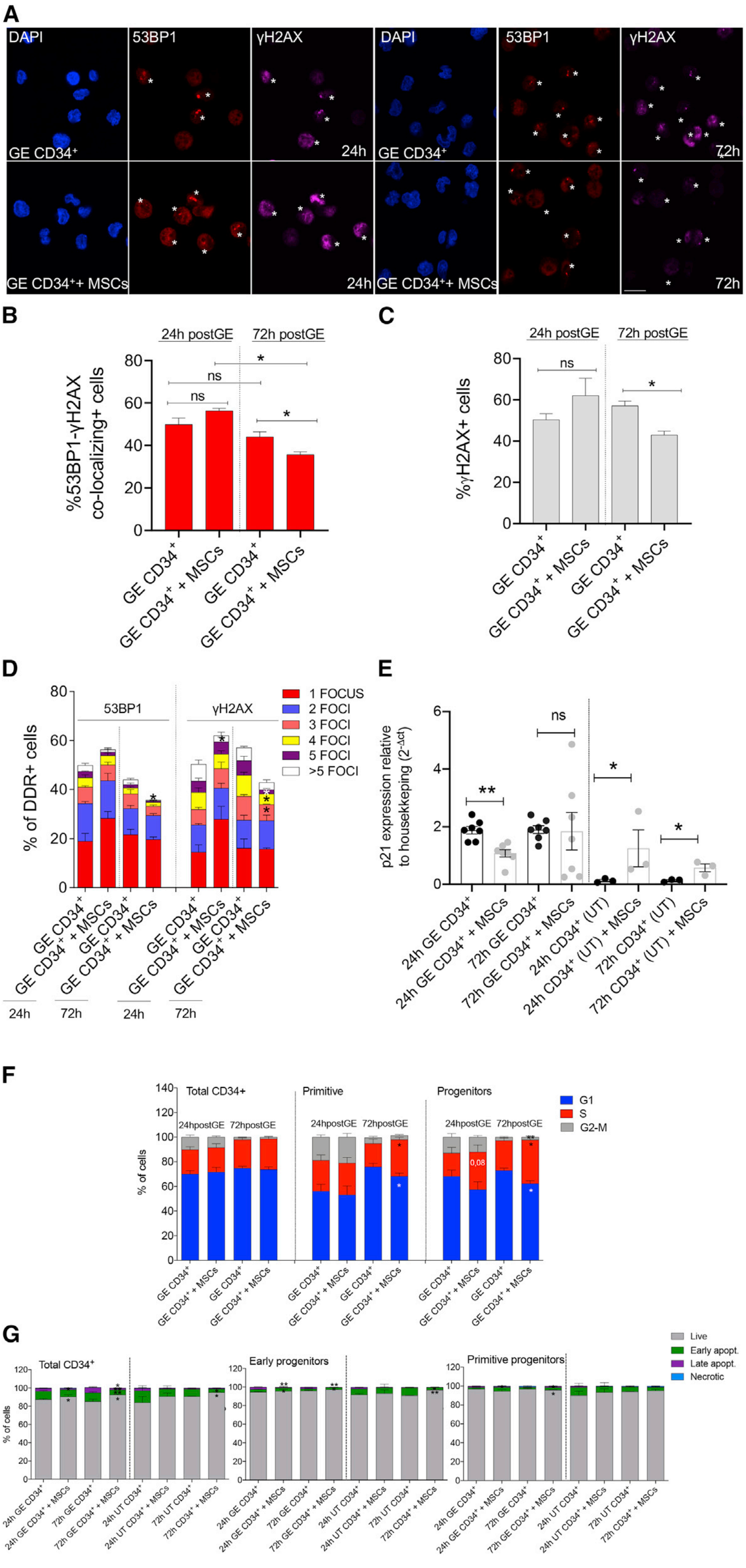

Figure 4. MSCs accelerate DDR foci resolution, preventing the cell-cycle delay and reducing the level of apoptosis in GE-HSPCs

(A) Confocal images of 53BP1-positive foci (red), γH2AX-positive foci (purple), and DAPI-positive nuclei (blue) in HSPCs gene edited according to standard procedures (GE CD34$^+$) or in the presence of MSCs (GE CD34$^+$ + MSCs) at 24 and 72 h upon gene editing. Asterisks indicate positive foci. Scale bar represents 20 μm. (B and C) Quantification of 53BP1-γH2AX co-localizing foci (B) and all γH2AX (C) foci from (A); 24 h (53BP1): n = 4; 72 h (53BP1): n = 4; 24 h (γH2AX): n = 4; 72 h (γH2AX): n = 3. Each error bar shows means ± SEM. p values were determined by Mann-Whitney test (ns $p > 0,05$; $^*p < 0.05$). (D) Quantification of 53BP1- and γH2AX-positive foci from (A)–(C). Positive cells were divided in subgroups according to the number of foci identified per cell (from 1 to more than 5). 24 h (53BP1): n = 4; 72 h (53BP1): n = 4; 24 h (γH2AX): n = 4; 72 h (γH2AX): n = 3. Each error bar shows means ± SEM. p values were determined by Mann-Whitney test for each subgroup (only significant comparisons were reported; $^*p \leq 0.05$). (E) qPCR expression analysis of *p21* in untreated (UT) and gene-edited (GE) HSPCs co-cultured with MSCs or in plastic dishes for 24 and 72 h. Each error bar shows means ± SEM (n ≥ 3). Gene expression was calculated as $2^{-\Delta CT}$ relative to *GUSB* gene. (F) Percentage of GE-HSPCs (distinguished between primitive cells and progenitors) in the indicated cell-cycle phases measured at 24 (n = 7) and 72 h (n = 6) after editing. Significance was calculated for each time point comparing GE CD34$^+$ + MSCs with GE CD34$^+$. Each error bar shows means ± SEM. p values were determined by Mann-Whitney test for each cell-cycle phase (only significant comparisons were reported; $^*p \leq 0.05$). (G) Percentage of apoptotic cells within different GE-HSPC subsets (primitive cells and progenitors) at the indicated time points after gene editing. Live: Annexin V$^-$, 7AAD$^-$; early apoptotic: Annexin V$^+$, 7AAD$^-$; late apoptotic: Annexin V$^+$, 7AAD$^+$; necrotic: Annexin V$^-$, 7AAD$^+$. n = 3 for each time point analyzed.

(Figure S7B). Conversely, we showed a significant reduction of MIP-1a and SCF in the MSC-conditioned medium (Figure S7C).

We next performed RNA sequencing analysis to identify genes specifically modulated in MSCs compared with MSCs exposed to GE-CD34$^+$ cells (GEO: GSE168834). Principal-component analysis (PCA) showed a strong separation of samples belonging to the two groups (Figure S8A), which was also reflected in the high number of differentially expressed genes (DEGs) (false discovery rate [FDR] < 0.01 and |logFC| > 2) between MSCs and MSCs co-cultured with HSPCs treated for GE. In particular, we found a total of 2,551 DEGs, of which 2,307 were upregulated and 244 were downregulated (Table S1). Gene set enrichment analysis (GSEA) against the Gene Ontology (GO)-Biological Processes (BP) database on genes ranked by log fold change (FC) values highlighted the upregulation of pathways involved in cytokine secretion, response to external signals, including inflammatory molecules, cell-cycle control, and DNA-repairing mechanisms in MSCs co-cultured with GE-HSPCs (Figures S8B–S8D).

In the attempt to identify MSC factors supporting GE-HSPCs, we further analyzed the cytokine gene profiling of MSCs co-cultured with GE-HSPCs compared with MSCs alone. In MSCs co-cultured with GE-HSPCs, we found an enriched expression of TNF and TNF superfamily cytokines (Figures S8D and S8E), known to facilitate HSPC functions[58] and to enhance the anti-inflammatory activity of MSCs, increasing their survival,[59] (Figure S8C) and the MSC production of IL-10 and other supportive factors.[60]

We also observed that MSCs massively expressed *IL-10* and *IL-18* after co-culture with GE-HSPCs (Figure S8E). The release of IL-10 could mediate the reduction of inflammatory cytokines in GE-HSPCs,[48] whereas IL-18 was described to control HSC quiescence in a murine setting.[61] We also found that MSCs express a higher level of *IL-1B* after co-culture with GE-HSPCs. The inflammatory milieu associated with the GE procedure could induce *IL-1B* expression in MSCs,[62] acting as a positive loop to increase the anti-inflammatory and supporting properties of MSCs.

Altogether, our results suggested that MSCs might be capable of sensing the inflammatory environment caused by the activation of the DNA-damage-induced stress signals in GE-HSPCs, activating, as a consequence, a pro-survival and anti-inflammatory signaling program to sustain GE cells (Figures 4 and S6–S8; Table S2).

We concluded that BM-MSCs contributed to faster resolution of DNA damage foci, in addition to reducing the culture stress in HSPCs treated for GE through the secretion of supportive, anti-inflammatory, and pro-survival factors.

MSC paracrine activity and cell-to-cell contact are required to support human HSPCs in several pre-clinical applications of GE technology

We further dissected the mechanisms of MSC function in support of GE-HSPCs by analyzing the phenotype, the absolute number of phenotypically primitive GE-HSPCs, and the expression of pro-apoptotic, inflammatory, and survival genes in HSPCs gene edited according to our standard protocol in the presence of MSCs (direct co-culture and transwell) and MSC-conditioned medium.

We observed that the MSC-conditioned medium alone supported the proliferation of GE-HSPCs (Figures 5A and 5B) and the maintenance of primitive GE-HSPCs less efficiently than our co-culture system (Figure 5C). The absolute count of phenotypically primitive GE-HSPCs and HSPCs gene edited by HDR (GFP$^+$) was higher in the presence of MSC-conditioned medium than in the standard protocol conditions (Figure 5D), suggesting that MSCs are required to efficiently support GE-HSPCs. The level of *IL-8* expression decreased in HSPCs cultured in the MSC-conditioned medium and even more efficiently in the presence of MSCs (both direct contact and transwell co-culture) (Figure 5E).

The expression level of pro-survival genes *BCL2α* and *BCL2β* increased in GE-HSPCs exposed to the conditioned medium and co-cultured with MSCs. On the contrary, *p21* was significantly downregulated only in the presence of MSCs (Figure 5F).

We concluded that the direct contact with MSCs allows for a superior efficiency to protect HSPCs from the detrimental effects of GE, possibly because the cells represent a continuous source of hematopoietic supportive factors in addition to controlling HSPC homeostasis through direct cell contact in co-culture.

We also determined whether the use of MSCs could be clinically relevant to support HSPCs undergoing editing-based gene disruption (knockout [KO]). We used our experimental setting to co-culture KO-edited HSPCs on an MSC feeder, and we performed KO of the AAVS1 locus with a previously published high-specificity guide RNA.[35] We analyzed the total cell count, the phenotype, and the absolute number of HSPCs with primitive phenotype 72 h after editing. We observed a higher number of KO-edited HSPCs and reduced mortality in the presence of MSCs (Figures S9A and S9B). MSCs also induced an expansion of hematopoietic progenitors while preserving the primitive HSPC subset (Figures S9C and S9D). The absolute number of KO-edited HSPCs with a primitive phenotype increased in the presence of MSCs. These results correlate with the upregulation of pro-survival genes and reduced expression of *BAX* in KO-edited HSPCs co-cultured with MSCs (Figure S9E). On the contrary, we did not observe a significant impact on the expression of cell-cycle arrest and inflammatory-related genes (Figures S9F and S9G), consistent with the observation that the activation of a DDR stress response and inflammatory program was more exacerbated in HSPCs undergoing HDR-mediated GE.[35]

MSCs exert pro-survival and anti-inflammatory effects also on mobilized PB (mPB) GE CD34+ cells, resulting in an improved transplantation outcome

Considering that mPB CD34$^+$ cells represent a clinically relevant source of HSPCs for transplantation, we applied our MSC-based co-culture system also to support mPB CD34$^+$ cells undergoing GE.

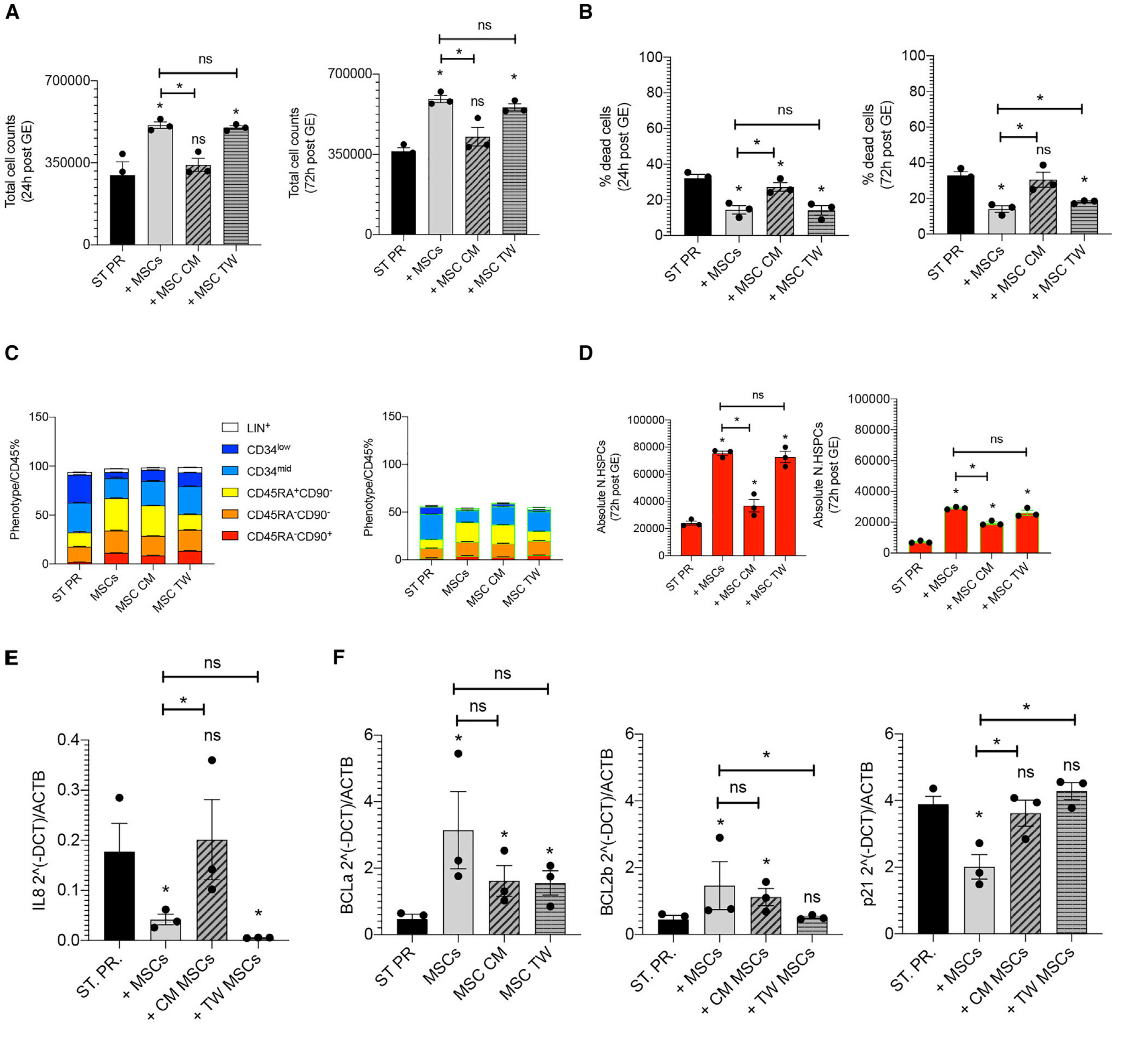

Figure 5. Comparison of the supportive capacity of MSC-conditioned medium, direct contact, and indirect transwell co-culture

(A and B) Total cell count (A) and percentage of death (B) of GE-HSPCs recovered for 24 (left panel) and 72 h (right panel) after editing in the presence of MSC-conditioned medium and in direct (+MSCs) and indirect transwell co-culture (+MSC TW) with BM-MSCs. GE-HSPCs maintained in culture according to our standard protocol conditions were used as control (ST PR). (C and D) Phenotypic analysis of GE-HSPCs expanded in culture for 72 h after editing in the presence of MSC-conditioned medium, in direct (+MSCs) and indirect transwell co-culture (+MSC TW) with BM-MSCs, and according to our standard protocol (ST PR). The absolute number of phenotypically primitive GE-HSPCs and HSPCs GE by HDR (GFP^+) is reported in (D). (E and F) Gene-expression analysis of inflammatory (E), pro-survival, and cell-cycle genes (F) in GE-HSPCs expanded in culture for 72 h after editing in the presence of MSC-conditioned medium, in direct (+MSCs) and indirect transwell co-culture (+MSC TW) with MSCs, and according to our standard protocol (ST PR). In our experimental setting, we could not distinguish properly the effects of direct contact from transwell MSC co-culture on GE-HSPCs since the majority of UCB CD34+ pre- and post-gene editing migrated toward the MSC feeder in the transwell bottom during the co-culture. Indeed, MSCs secrete SDF1α, a potent chemoattract for HSPCs.[53] For all experiments, each error bar shows means ± SEM ($n \geq 3$). p values were determined by Mann-Whitney test (*$p \leq 0.05$).

After 3 days of expansion in the presence of BM-MSCs, mPB $CD34^+$ cells were gene edited and recovered for 72 h on an MSC feeder. mPB $CD34^+$ cells gene edited according to our standard protocol were used as a control (Figure 6A). The total cell count of GE-mPB $CD34^+$ cells significantly increased in co-culture with MSCs (Figure 6B). In the absence of MSCs, we observed a higher mortality rate after GE, and the number of cells retrieved after 72 h of culture was almost half of the initial number (1×10^5) of cells undergoing GE, and we also

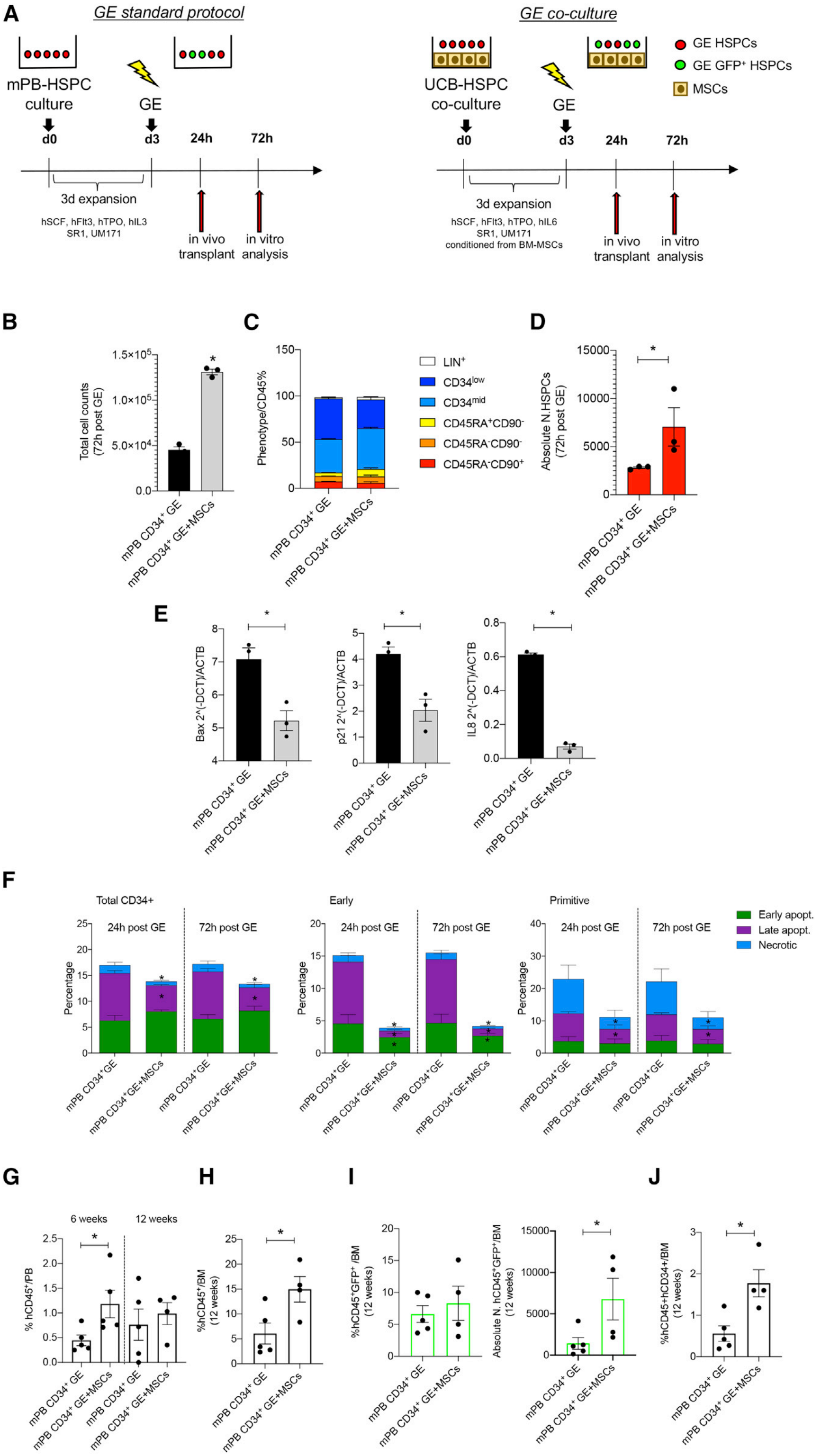

Figure 6. *In vitro* and *in vivo* analysis of the supporting activity of MSCs on GE mobilized PB (GE-mPB) HSPCs

(A) Schematic representation of the *in vitro* and *in vivo* experiments using GE-mPB CD34$^+$ cells. BM-MSCs were expanded for 72 h before co-culture with mPB HSPCs. Thereafter, mPB CD34$^+$ cells were pre-stimulated in co-culture with BM-MSCs in GE medium conditioned from BM-MSCs and similarly recovered upon gene editing. GE-mPB HSPCs were transplanted into NSG mice after 24 h recovery, while GE-HPSCs were maintained in culture with BM-MSCs for 72 h for the *in vitro* analysis. mPB HSPCs GE according to our standard protocol were used as controls for the transplantation experiments at 24 h after gene editing and for the *in vitro* analysis after 72 h of culture. (B and C) Total cell counts (B) and phenotypic analysis (C) of GE-mPB recovered in culture 72 h after gene editing in the presence of MSCs (mPB CD34$^+$ GE + MSCs). mPB HSPCs GE according to our standard protocol conditions were used as control (mPB CD34$^+$ GE). The absolute number of phenotypically primitive GE-mPB HSPCs is reported in (D). *BAX*, *p21*, and *IL-8* expression analysis is reported in (E), whereas evaluation of apoptosis in GEmPB HSPCs recovered in culture 72 h after gene editing in the presence (mPB CD34$^+$ GE + MSCs) or absence (mPB CD34$^+$ GE) of an MSC feeder is reported in (F). (G) Percentage of human cell engraftment (%hCD45$^+$) on total live cells in the PB of NSG mice transplanted with the outgrown of 0.5 $\times$ 10^6 mPB CD34$^+$ cells 24 h after GE in the presence of MSCs (mPB CD34$^+$ GE + MSCs) or according to our standard protocol (mPB CD34$^+$ GE) at 6 and 12 weeks after transplantation. (H) Analysis of human engraftment in the BM of transplanted mice at sacrifice (12 weeks). (I) Percentage (left panel) and absolute number (right panel) of HDR GE cells (GFP+) in the BM of transplanted mice at 12 weeks after transplantation. (J) Percentage of human cells positive for CD45 and CD34 engrafted on total live cells based on physical parameters in the BM after the transplantation of mPB CD34+ cells GE in the presence of MSCs (mPB CD34$^+$ GE + MSCs) or according to our standard protocol (mPB CD34$^+$ GE). For all plots, individual data points represent an animal. p values were determined by Mann-Whitney test (*$p \leq 0.05$; **$p \leq 0.001$).

observed an expansion of early progenitors when analyzing the phenotype of CD34$^+$ cells after editing (Figure 6C). The absolute number of GE-mPB CD34$^+$ cells with a primitive phenotype significantly increased in the presence of MSCs (Figure 6D). The percentage of HDR and non-homologous end joining (NHEJ) at 72 h post-editing in mPB cells gene edited in the presence of MSCs was similar to our standard controls (Figure S3G).

We reported a reduced expression of the pro-apoptotic gene *BAX*. *p21* was also downregulated in GE-mPB CD34$^+$ cells co-cultured with MSCs. In addition, we also found a robust reduction of *IL-8* in GE-mPB CD34+ cells co-cultured with MSCs (Figure 6E). Consistently, we found that a significant reduction of late apoptotic cells was observed in GE-mPB CD34$^+$ cells at 24 and 72 h after editing in the presence of MSCs. The pro-survival effects of MSCs were even more evident in early and primitive progenitors when dissecting the phenotype of co-cultured GE-mPB CD34$^+$ cells (Figure 6F). We also found a robust reduction of *IL-8* in GE-mPB CD34$^+$ cells co-cultured with MSCs (Figure 6E).

We then transplanted the culture equivalent of 0.5×10^6 mPB CD34$^+$ cells gene edited in the presence of MSCs or according to our standard protocol 24 h after *in vitro* recovery (Figure 6A). We followed the human cell engraftment in the PB, showing a higher engraftment of GEmPB CD34$^+$ cells + MSCs at 6 weeks after transplantation ($p = 0.019$), which is in line with the improved early-phase engraftment observed in our previous experiments using UCB CD34$^+$ cells (Figure 6G). At the endpoint, we observed a higher human engraftment ($p = 0.03$) and increased percentage of human CD34$^+$ cells in the BM of mice transplanted with mPB CD34$^+$ cells gene edited in the presence of MSCs ($p = 0.01$) (Figures 6H and 6J). In these samples, we also detected a higher number of human HDR-edited cells (CD45$^+$GFP$^+$) compared with our standard protocols (Figure 6I).

These data suggest that the pro-survival effects of MSCs on mPB CD34+ cells observed *in vitro* upon GE correlate with an improved transplantation efficacy.

DISCUSSION

In this study, we employed third-party human BM-MSCs as a supportive tool to sustain HSPCs undergoing GE. This strategy was based on pre-clinical and clinical data indicating that MSCs promote *ex vivo* expansion of unmanipulated HSPCs and facilitate their engraftment *in vivo*, thus improving the hematopoietic recovery of transplanted patients.[14–16,63] These data prompted us to exploit the hematopoietic supportive capacity of MSCs in the context of HSPC GE. In this setting, extensive *ex vivo* manipulation impacts on self-renewal and differentiation potential of HDR gene-edited autologous HSPCs, thus altering their LT repopulating capacity.[35] The activation of a strong DDR in cultured GE-HSPCs limits the clinical translation of GE technologies, potentially leading to a low dose of HDR-edited cells available for transplantation and accounting for delayed engraftment, oligoclonal hematopoietic reconstitution, and increased risk of graft failure in patients.

Our results demonstrate the ability of MSCs to favor the *ex vivo* expansion of GE-HSPCs and attenuate the loss of the host-repopulating HSPC subset, this translating into an improved transplantation outcome. While the *ex vivo* expansion effect driven by MSCs has been previously reported in unmanipulated HSPCs co-cultured with MSCs,[15,16] no detailed analysis has been performed so far on the impact of MSC co-culture on the phenotype and functionality of HSPCs upon GE procedure. To this aim, we developed a novel strategy to dissect the phenotypic composition of *ex vivo* cultured HSPCs. We observed that MSCs were capable of preserving the phenotypically primitive and LT repopulating HSPC subset upon GE when activation both by *ex vivo* expansion and the GE procedure occur. Indeed, our co-culture system allowed us to significantly increase the total number of CD34$^+$ cells and the number of phenotypically primitive Lin$^-$, CD34^{high}, CD90$^+$, CD45RA$^-$ cells available for transplantation (Figure 2), resulting in improved engraftment of human CD45$^+$ and faster hematological reconstitution when HSPCs, gene edited in the presence of MSCs, were transplanted into NSG mice. In particular, the ability of MSCs to favor the proliferation of early progenitors while preserving the phenotypically primitive HSPC subset allowed a faster and more polyclonal early-phase reconstitution, without affecting the clonogenic potential of LT repopulating cells, as also demonstrated by the BAR-seq analysis, which indicates an increase in repopulating HDR-edited clones during the early-phase reconstitution. Indeed, an enrichment of gene-edited cells was found at sacrifice in the BM of mice transplanted with CD34$^+$ cells treated for GE in the presence of MSCs (Figure S4G), indicating a specific supportive effect of MSCs on GE cells.

Limited information is available on the mechanisms by which MSCs support HSPC functions. Our data indicate that MSCs may act on DDR-dependent cell-cycle dynamics and mitigate the apoptotic and inflammatory cascade occurring in GE-HSPCs. Together with a p53-mediated DDR activation, inflammatory gene categories were reported by some of us to be enriched in GE-HSPCs.[35] Even if basal levels of key pro-inflammatory cytokines, such as TNF-α, are reported to support HSC survival and regeneration during inflammation,[64] several studies have highlighted the detrimental effects of chronic inflammatory programs in HSPCs,[50,65,66] which may alter their fitness, induce their premature differentiation, reduce the efficacy of HSCT, and even predispose to acute myeloid leukemia (AML) or myelodysplastic syndrome (MDS) development.[67] Given the DDR-dependent proliferative defect and the concomitant activation of an inflammatory cascade in GE-HSPCs, we reasoned that the use of MSCs could represent a valid approach to simultaneously counteract both mechanisms. Importantly, we observed that in response to GE, MSCs favored a faster resolution of DNA damage foci in HSPCs, reduced *p21* induction, and prevented the cell-cycle arrest observed upon standard GE procedures without affecting cell-cycle progression of UT cells (Figures 4 and S6).

Moreover, we also reported that MSCs reduced the level of apoptosis in all GE-HSPC subsets by inducing the expression of pro-survival

genes in GE-HSPCs (Figure S3), in line with previous works showing MSC pro-survival ability in injured cells.[68]

In addition, HSPCs gene edited in the presence of MSCs displayed a significant reduction of the culture-induced proliferative stress, as shown by the reduced accumulation of γH2AX foci at early and late time points after editing. We also reported a lower percentage of 53BP1-γH2AX foci-positive cells in HSPCs treated for GE in the presence of MSCs at later time points. This evidence is consistent with the reported role of MSCs in mitigating DNA damage or replication stress in *in vitro* expanded murine HSCs.[69] Despite the reduced presence of nuclear 53BP1, the efficiency of on-target repair by HDR editing in HSPCs co-cultured with MSCs was similar to standard protocol GE-HSPCs, as previously reported.[44]

Moreover, in support of this finding, we have shown the enrichment of several supportive factors in the medium collected from GE-HSPCs co-cultured with MSCs (Figure S4), suggesting that MSC paracrine activity plays a fundamental role in the control of proliferation, DNA repair, apoptosis, culture-induced stress, and maintenance of GE-HSPC stemness.

With respect to the activation of an inflammatory cascade reported in HSPCs treated for GE, MSCs have been reported to display a unique capacity to sense environmental signaling and activate a specific paracrine program to sustain neighboring cells and reduce the level of inflammation.[48] While the anti-inflammatory capacity of MSCs has been already described in several disease animal models and in patients,[70–73] in our work, we point to a new role of MSCs in the control of the inflammatory cascade occurring during HSPC-GE procedures, possibly through the release of anti-inflammatory molecules.[48] Indeed, we reported the transcriptional downregulation of *IL-8* and *CCL2,* two key inflammatory factors related to HSPC dysfunction and reduced *ex vivo* expansion,[74] when GE-HSPCs are cultured in the presence of MSCs compared with controls (Figures S6I and S6J).

In accordance with our data, it has been described that inflammatory cytokines prime MSCs toward an anti-inflammatory and pro-survival phenotype[75] that may reduce the inflammation of HSPCs after GE, favoring the maintenance of the HSPC primitive subset. Similarly, in response to the release of damage-associated molecules, MSCs produce indoleamine dioxygenase (IDO), an enzyme with a wide range of anti-inflammatory properties.[76] Moreover, IDO is also responsible for enhancing MSCs anti-inflammatory properties by reducing IL-8 production.[77]

In conclusion, our findings suggest possible molecular mechanisms responsible for the MSC-based proliferative and functional support of GE-HSPCs. In line with these findings, GSEA against the GO-BP database on RNA sequencing (RNA-seq) data of MSCs showed the activation of regulatory pathways involved in the control of inflammation and the cell cycle, in addition to an enriched set of genes involved in cytokine production and release, in MSCs co-cultured with GE-HSPCs (Figures 5B–5D; Table S2). Interestingly, we found several cytokines in the MSC-conditioned medium upon co-culture with GE-HSPCs that were enriched in our GSEA dataset, confirming the supportive paracrine activity of MSCs (Table S1; Figure S4).

Moreover, our results indicate that the MSC supportive activity protecting HSPCs from the detrimental effects of GE is superior when GE-HSPCs are directly co-cultured with MSCs (Figure 6), highlighting the dual role of MSCs in the control of HSPC homeostasis by employing both cell-to-cell contact and paracrine mechanisms.[78,79] In the co-culture setting, MSCs represent a continuous source of hematopoietic supportive factors, in addition to controlling HSPC homeostasis through direct cell-to-cell contact. In support of this observation, LT repopulating HSPCs are associated with osteoprogenitors in the BM niche,[2] and they show a more primitive phenotype if in contact with MSCs *in vitro*.[80]

Collectively, our data define an MSC-based co-culture system to sustain GE-HSPCs with the ultimate goal of preserving their functions in HSCT. Altogether, our results demonstrate that MSCs are capable of efficiently increasing the number of HDR gene-edited HSPCs, mitigating the hurdle concerning the low cell dose available for transplantation. The hematopoietic supportive activity of MSCs favored not only HSPC expansion but also the maintenance of phenotypically primitive HSPCs in culture, in addition to protecting HSPCs from cell-cycle delay and excessive activation, which are associated with GE procedures and exacerbated by *ex vivo* culture, leading to improved HSPC engraftment and transplantation outcomes. The MSC-driven effect on the early phase of hematological reconstitution was also supported by the increased number of GE-HSPC clones detected through the barcoded vector and demonstrates for the first time the ability of MSCs to sustain polyclonal reconstitution after HSCT.

Similar results were obtained *in vitro* when MSCs were co-cultured with GE-mPB $CD34^+$ cells, highlighting the possibility of improving the outcome of GE also when HSPCs derived from PB are employed, these representing a very relevant source for clinical application. In these experiments, we also observed a positive correlation between the number of GE-HSPCs with a primitive phenotype in culture with BM-MSCs and the level of human engraftment, confirming that the increased dose of HSPCs mediated by BM-MSCs is associated with an increased level of engraftment of GE cells in humanized model of transplantation.

Finally, we showed that MSC co-culture could be useful to also support HSPCs undergoing editing-based gene disruption.

A prompt and sustained hematological reconstitution represents a desirable goal for patients undergoing HSCT who are at risk of graft failure and of developing life-threatening infections, especially in the early post-transplant phase. Our data provide a proof of principle that in the setting of GE, where extensive *ex vivo* manipulation may impact on self-renewal and differentiation potential of HSPCs, MSC-based approaches could be employed to counteract GE-related detrimental effects, thus optimizing transplantation outcome.

The clinical implementation of such MSC-based approaches would require their testing within experimental trials given that technologies for large-scale and clinical-grade expansion of MSCs have been extensively optimized and employed in the clinical setting. MSC co-culture could be employed alone or in combination with other emerging strategies to increase HDR efficiency, such as transient p53 inhibition and adenovirus 5 E4orf6/7 protein expression.[27]

Since MSC-derived secretome is emerging as a medicinal product for the treatment of several inflammatory conditions,[81] this solution may be practically advantageous also in the setting of GE-HSPCs and might be employed in combination with the GE enhancers to preserve HSPCs and improve the GE-HSPC transplantation outcome.

MATERIALS AND METHODS

Isolation of human BM-derived MSCs

MSCs were isolated from residual BM aspirates of healthy donors (median age: 12 years; range: 4–18 years), who donated BM for transplantation at the San Raffaele Scientific Institute, after obtaining informed consent according to TIGET09 research protocol. TIGET09 is a protocol for the collection of biological material from healthy volunteers and patients with rare genetic diseases. It was approved by the Ethics Committee of San Raffaele Hospital on June 7th, 2017. MSCs were isolated according to a previously published protocol.[36]

Expansion of human UCB-HSPCs on MSC feeder

MSCs were plated at a density of 10,000 cells/cm^2 and expanded for 3 days in proper MSC medium. Twenty-four h before starting the co-culture experiment, MSC medium was replenished with HSPC medium with or without proper pre-stimulating factors for conditioning. Commercial UCB CD34$^+$ HSPCs (Lonza, catalog 2C-101) were thawed at a density of 5×10^5/mL and expanded in MSC-conditioned HSPC medium. As control, UCB CD34$^+$ HSPCs were thawed and expanded in HSPC medium with or without the addition of proper stimulating cytokines (100 ng/μL hSCF, 100 ng/μL hFLT3, 20 ng/μL hTPO, 20 ng/ μL hIL-6). In the case of GE, SR1 (1 μM) and UM171 (50 nM) were added to the cytokine mix. After 72 h of culture, UCB CD34$^+$ HSPCs were collected by gentle pipetting and analyzed in terms of total cell count, phenotype, and clonogenic capacity.

LT expansion of GE-HSPCs on MSC feeder

MSC feeder was prepared as described above including medium conditioning. Commercial UCB CD34$^+$ HSPCs (Lonza, catalog 2C-101) were edited following standard procedures[31] and plated at a density of 5×10^5 cells/mL on an MSC feeder in HSPC medium conditioned by MSCs for 72 h expansion. GE-HSPCs expanded for 72 h on plastic in the absence of MSCs were used as controls.

Expansion and recovery of human HSPCs undergoing GE on MSC feeder

MSC feeder was prepared as described above including medium conditioning. Commercial UCB CD34$^+$ HSPCs (Lonza, catalog 2C-101) undergoing GE were thawed at a density of 5×10^5/mL on an MSC feeder and expanded for 3 days on an MSC feeder in HSPC medium conditioned by MSCs. After editing, GE HSPCs were maintained in culture for 24 h or expanded for 72 h on an MSC feeder at a density of 5×10^5/mL in HSPC medium conditioned by MSCs. Human HSPCs edited according to standard protocol without the support of an MSC feeder were used as controls. Commercial mPB CD34$^+$ cells (Stem Cell, catalog 70060) were thawed and expanded in HSPC medium (300 ng/μL hSCF, 300 ng/μL hFLT3, 100 ng/μL hTPO, 60 ng/μL hIL-3) with the addition of SR1 (1 μM) and UM171 (50 nM) in the presence or absence of MSCs. Upon GE, GE-HSPCs were recovered in culture on an MSC feeder using HSPC medium conditioned from MSCs or on plastic using HSPC medium.

Flow cytometry

The capacity of MSCs to preserve HSPCs in culture was evaluated by flow cytometry analysis using the BD LSRFortessa. HSPCs were collected and washed with PBS + 2% FBS. 1×10^5 cells were incubated with the proper antibody mix for 10 min at room temperature (RT) in the dark. The following antibodies were used: CD16 PE (BD Biosciences, 332779); CD14 PE (BioLegend, 301806); CD3 PE (BD Biosciences, 345765); CD15 PE (BioLegend, 301906); CD56 PE (BD Biosciences, 345812); CD19 PE (BD Biosciences, 345789); CD34 BV421 (BioLegend, 343610); CD45RA APC-H7 (BioLegend, 304128); CD45 BUV395 (BD Biosciences, 563792); and CD90 APC (BD Biosciences, 559869) for 10 min at RT in the dark. After washing with PBS + 2% FBS, cells were centrifuged for 5 min at 1,500 RPM and resuspended into 100 μL PBS + 2% FBS. Unstained cells were used as a negative control. All flow cytometry assays were standardized using SPHERO Rainbow Calibration Particles (8 peaks) (Spherotech, RCP305A). The primitive HSPC cell subset was phenotypically identified as CD45$^+$, Lin$^-$, CD34^{high}, CD45RA$^-$, CD90$^+$ cells. Human cell engraftment and hematological reconstitution were determined on PB (50 μL) and BM samples (100 μL) of transplanted mice by flow cytometry. Red blood cells were lysed using ammonium-chloride-potassium (ACK) lysing solution for 15 min at RT, after the addition of precision counting beads (Biolegend, 424902). After washing twice with PBS + 2% FBS, cells were stained with the following antibody mix: CD45 APC (Biolegend, 304037); CD3 PE (BD Biosciences, 345765); CD19 PE Cy7 (BD Biosciences, 302216); CD33 VioBlue (Biolegend, 130-099-485); and CD13 PerCP Cy5.5 (BD Biosciences, 561361). BM samples were processed using the same protocol and stained with the following antibody mix: CD34 PB (Biolegend, 343511) PB and CD45 APC (Biolegend, 304037). Samples were run on BD FACSCanto II cytometer (BD Biosciences). At least 10,000 were recorded. Analysis of all fold cytometry results was performed using FlowJo software (Tree Star).

Cell-cycle phase analysis by EdU/Hoechst staining

0.5–1 $\times 10^5$ cells were treated with 2 μM EdU for 4 h in culture (Thermo Fisher Scientific, C10636), washed with PBS + 1% BSA, and stained with the following antibodies mix for 15 min at 4°C: CD34 PE (Miltenyi Biotec 130-081-00), CD90 APC (BD Biosciences,

559869), and CD133/1 PECy7 (Miltenyi Biotec, 130-101-652). Cells were then fixed with 100 μL of Click-iT fixative for 15 min at RT upon cell-surface staining to distinguish the different HSPC subsets. After washing with PBS + 1% BSA, cells were permeabilized with 100 μL 1X Click-iT saponin for 15 min. Detection of EdU-DNA was performed by incubating cells with 500 μL Click-iT Plus reaction cocktail for 30 min at RT protected from light. Cells were subsequently washed with PBS + 1% BSA before overnight DNA staining with Hoechst at RT protected from light. Fluorescence was measured by flow cytometry on FACSymphony A5 SORP (BD Biosciences). Analysis of flow cytometry results was performed using FlowJo software.

Apoptosis assay

Cell apoptosis was evaluated using Pacific Blue Annexin V (Biolegend, 640918) and 7-AAD Viability Staining Solution (BioLegend, 420403). In detail, GE-HSPCs were stained with the following antibodies mix for 15 min at 4°C: CD34 PE (Miltenyi Biotec 130-081-00), CD90 APC (BD Biosciences, 559869), and CD133/1 PECy7 (Miltenyi Biotec, 130-101-652). Cells were then washed with diluted 1:10 10X Annexin V Binding Buffer (BD Pharmingen, 556454) upon surface marker staining and stained with Pacific Blue Annexin V (Biolegend, 640918) and 7-AAD Viability Staining Solution (BioLegend, 420403) for 15 min at RT in the dark. After staining, cells were washed in 1X Annexin V Binding Buffer and acquired in 10 min. All samples were run on BD FACSCanto II cytometer (BD Biosciences). At least 10,000 were recorded. Analysis of flow cytometry results was performed using FlowJo software.

Immunofluorescence analysis

Multitest slides (MP Biomedicals, 096041505) were coated with Poly-L-lysine solution (Sigma-Aldrich, P8920-500ML). After two washes with PBS solution, 0.3–0.5 × 10^5 cells were seeded on covers for 20′ and fixed with 4% PFA (Santa Cruz Biotechnology, sc-281692) for 20′. Cells were then permeabilized with 0.1% Triton X-100. After blocking with 0.5% BSA and 0.2% fish gelatin in PBS, cells were stained with the indicated primary antibodies (53BP1 antibody, Bethyl Laboratories; anti-phospho histone H2A.X (Ser139) antibody, clone JBW301, Merck). Cells were than washed with PBS and incubated with Alexa 568- and 647-labeled secondary antibodies (Invitrogen). Nuclear DNA was stained with DAPI at 0.2 μg/mL concentration (Sigma-Aldrich, D9542), and covers were mounted with Aqua-Poly/Mount solution (TebuBio, 18606-20) on glass slides (Bio-Optica). Fluorescent images were acquired using Leica SP5 confocal microscopes. Quantification of DDR foci in immunofluorescence images was conducted using Cell Profiler.

Mice

NSG mice were purchased from The Jackson Laboratory and maintained in specific-pathogen-free (SPF) conditions. The procedures involving animals were designed and performed with the approval of the Animal Care and Use Committee of the San Raffaele Hospital (IACUC #1039 and #1068) and communicated to the Ministry of Health and local authorities according to Italian law.

GE-HSPC xenotransplantation experiments

Seven-week-old NSG mice were sublethally irradiated (150–180 Gy) and intravenously transplanted. 1 × 10^5 HSPCs (t0 culture equivalent) were gently collected by pipetting and injected 24 h after GE according to standard procedures or in the presence of MSCs. In the case of GE mPB $CD34^+$ cell transplantation, the outgrown cells of the 0.5 × 10^6 dose were transplanted as described above. In both mouse models, human cell engraftment and the presence of HDR gene-edited cells were monitored by serial collection of PB from the mouse tail, and, at the end of the experiment (16 weeks after transplantation), BM was collected for endpoint analyses.

Data availability

All relevant data are included in the manuscript. Sequencing data are deposited in the Gene Expression Omnibus with the access code GSE206904: RNA-seq (GSE168834) and BAR-Seq (GSE206900).

SUPPLEMENTAL INFORMATION

Supplemental information can be found online at https://doi.org/10.1016/j.ymthe.2022.08.011.

ACKNOWLEDGMENTS

We thank all members of L.N.'s, A.A.'s, and R.D.M.'s laboratories for critical discussion, the San Raffaele Hospital Flow Cytometry Facility (FRACTAL), the San Raffaele Center of Omics Sciences (COSR), and the San Raffaele Hospital Advanced Light and Electron Microscopy BioImaging Center (ALEMBIC). We thank Giulia Schiroli for technical support and critical discussion in the first phase of the project. We thank Marilena Mancino, Giada De Ponti, Margherita Berti, Giulia Porro and Maria Antonia Avanzini for technical support. This work was supported by the SR-TIGET Core grant from Fondazione Telethon TIGET-E6 to M.E.B., TIGET-E5 to R.D.M., and TIGET-E4 to L.N. L.N. is also supported by the Louis-Jeantet Foundation through the 2019 Jeantet-Collen Prize for Translational Medicine. R.D.M. is a New York Stem Cell Foundation - Robertson Investigator. A.C. was supported by the Lady Tata Memorial Trust International Award for Research in Leukemia 2020–2021 and is a European Hematology Association and American Society of Hematology Translational Reasearch Training in Hematology (EHA-ASH TRTH) Investigator.

AUTHOR CONTRIBUTIONS

S.C. designed the study, performed most of the experiments, analyzed results, and wrote the paper; A.C. performed the DDR experiments, interpreted data, and wrote the paper; V.V. gene edited UCB-HSPCs and helped with critical discussion; S.B. performed all the bioinformatics analysis and helped with critical discussion; S.F. generated the barcode DNA library and helped with bioinformatics data interpretation and critical discussion; S.S. and L.B.R. helped with the immunophenotypic analysis of GE-HSPCs; S.R., R.B., and S.P. helped with *ex vivo* MSC co-culture experiments; R.J.H. and L.S. helped with the xenograft transplantation experiments; I.M., R.D.M., P.G., A.A., and L.N. critically read the manuscript and helped

with scientific discussion; M.E.B. designed the study, analyzed data, and contributed to the final writing of the paper.

DECLARATION OF INTERESTS

L.N., P.G., and S.F. are inventors of patents on applications of GE in HSPCs owned and managed by the San Raffaele Scientific Institute and the Telethon Foundation. L.N. is the founder and a quota holder and P.G. is a quota holder of GeneSpire, a startup company aiming to develop *ex vivo* GE in genetic diseases. The remaining authors have no conflicts of interest to declare.

REFERENCES

1. Méndez-Ferrer, S., Michurina, T.V., Ferraro, F., Mazloom, A.R., Macarthur, B.D., Lira, S.A., Scadden, D.T., Ma'ayan, A., Enikolopov, G.N., and Frenette, P.S. (2010). Mesenchymal and haematopoietic stem cells form a unique bone marrow niche. Nature *466*, 829–834.
2. Morrison, S.J., and Scadden, D.T. (2014). The bone marrow niche for haematopoietic stem cells. Nature *505*, 327–334.
3. Asada, N., Takeishi, S., and Frenette, P.S. (2017). Complexity of bone marrow hematopoietic stem cell niche. Int. J. Hematol. *106*, 45–54.
4. Dominici, M., Le Blanc, K., Mueller, I., Slaper-Cortenbach, I., Marini, F., Krause, D., Deans, R., Keating, A., Prockop, D.J., and Horwitz, E. (2006). Minimal criteria for defining multipotent mesenchymal stromal cells. The International Society for Cellular Therapy position statement. Cytotherapy *8*, 315–317.
5. Bernardo, M.E., and Fibbe, W.E. (2015). Mesenchymal stromal cells and hematopoietic stem cell transplantation. Immunol. Lett. *168*, 215–221.
6. Noort, W.A., Kruisselbrink, A.B., in't Anker, P.S., Kruger, M., van Bezooijen, R.L., de Paus, R.A., Heemskerk, M.H.M., Löwik, C.W.G.M., Falkenburg, J.H., Willemze, R., and Fibbe, W.E. (2002). Mesenchymal stem cells promote engraftment of human umbilical cord blood-derived CD34(+) cells in NOD/SCID mice. Exp. Hematol. *30*, 870–878.
7. Almeida-Porada, G., Porada, C.D., Tran, N., and Zanjani, E.D. (2000). Cotransplantation of human stromal cell progenitors into preimmune fetal sheep results in early appearance of human donor cells in circulation and boosts cell levels in bone marrow at later time points after transplantation. Blood *95*, 3620–3627.
8. Masuda, S., Ageyama, N., Shibata, H., Obara, Y., Ikeda, T., Takeuchi, K., Ueda, Y., and Ozawa, K. (2009). Cotransplantation with MSCs improves engraftment of HSCs after autologous intra-bone marrow transplantation in nonhuman primates. Exp. Hematol. *37*, 1250–1257.e1251.
9. Fernandez-Garcia, M., Yañez, R.M., Sánchez-Domínguez, R., Hernando-Rodriguez, M., Peces-Barba, M., Herrera, G., O'Connor, J.E., Segovia, J.C., Bueren, J.A., and Lamana, M.L. (2015). Mesenchymal stromal cells enhance the engraftment of hematopoietic stem cells in an autologous mouse transplantation model. Stem Cell Res. Ther. *6*, 165.
10. Poloni, A., Leoni, P., Buscemi, L., Balducci, F., Pasquini, R., Masia, M.C., Viola, N., Costantino, E., Discepoli, G., Corradini, P., et al. (2006). Engraftment capacity of mesenchymal cells following hematopoietic stem cell transplantation in patients receiving reduced-intensity conditioning regimen. Leukemia *20*, 329–335.
11. Fibbe, W.E., and Bernardo, M.E. (2014). Control of immune responses by mesenchymal stromal cells. Rinsho Ketsueki *55*, 2190–2194.
12. Crippa, S., and Bernardo, M.E. (2018). Mesenchymal stromal cells: role in the BM niche and in the support of hematopoietic stem cell transplantation. Hemasphere *2*, e151.
13. Uccelli, A., Moretta, L., and Pistoia, V. (2006). Immunoregulatory function of mesenchymal stem cells. Eur. J. Immunol. *36*, 2566–2573.
14. Wagner, W., Roderburg, C., Wein, F., Diehlmann, A., Frankhauser, M., Schubert, R., Eckstein, V., and Ho, A.D. (2007). Molecular and secretory profiles of human mesenchymal stromal cells and their abilities to maintain primitive hematopoietic progenitors. Stem Cells *25*, 2638–2647.
15. Robinson, S.N., Ng, J., Niu, T., Yang, H., McMannis, J.D., Karandish, S., Kaur, I., Fu, P., Del Angel, M., Messinger, R., et al. (2006). Superior ex vivo cord blood expansion following co-culture with bone marrow-derived mesenchymal stem cells. Bone Marrow Transplant. *37*, 359–366.
16. de Lima, M., McNiece, I., Robinson, S.N., Munsell, M., Eapen, M., Horowitz, M., Alousi, A., Saliba, R., McMannis, J.D., Kaur, I., et al. (2012). Cord-blood engraftment with ex vivo mesenchymal-cell coculture. N. Engl. J. Med. *367*, 2305–2315.
17. Wang, W., et al. (2015). Notch Receptor-ligand Engagement Maintains hematopoietic stem cell quiescence and niche Retention. Stem Cells *33*, 2280–2293.
18. Emmons, R.V., et al. (1997). Retroviral gene transduction of adult peripheral blood or marrow-derived CD34+ cells for six hours without growth factors or on autologous stroma does not improve marking efficiency assessed in vivo. Blood *89*, 4040–4046.
19. Moore, K.A., Deisseroth, A.B., Reading, C.L., Williams, D.E., and Belmont, J.W. (1992). Stromal support enhances cell-free retroviral vector transduction of human bone marrow long-term culture-initiating cells. Blood *79*, 1393–1399.
20. Moritz, T., Patel, V.P., and Williams, D.A. (1994). Bone marrow extracellular matrix molecules improve gene transfer into human hematopoietic cells via retroviral vectors. J. Clin. Invest. *93*, 1451–1457.
21. Marktel, S., et al. (2019). Intrabone hematopoietic stem cell gene therapy for adult and pediatric patients affected by transfusion-dependent ss-thalassemia. Nat. Med. *25*, 234–241.
22. Aiuti, A., Roncarolo, M.G., and Naldini, L. (2017). Gene therapy for ADA-SCID, the first marketing approval of an ex vivo gene therapy in Europe: paving the road for the next generation of advanced therapy medicinal products. EMBO Mol. Med. *9*, 737–740.
23. Sessa, M., et al. (2016). Lentiviral haemopoietic stem-cell gene therapy in early-onset metachromatic leukodystrophy: an ad-hoc analysis of a non-randomised, open-label, phase 1/2 trial. Lancet *388*, 476–487.
24. Ferrua, F., Cicalese, M.P., Galimberti, S., Giannelli, S., Dionisio, F., Barzaghi, F., Migliavacca, M., Bernardo, M.E., Calbi, V., Assanelli, A.A., et al. (2019). Lentiviral haemopoietic stem/progenitor cell gene therapy for treatment of Wiskott-Aldrich syndrome: interim results of a non-randomised, open-label, phase 1/2 clinical study. Lancet. Haematol. *6*, e239–e253.
25. Ferrari, G., Thrasher, A.J., and Aiuti, A. (2020). Gene therapy using haematopoietic stem and progenitor cells. Nat. Rev. Genet.
26. Doudna, J.A. (2020). The promise and challenge of therapeutic genome editing. Nature *578*, 229–236.
27. Ferrari, A.V., Canarutto, D., Jacob, A., Castiello, M.C., Javed, A.O., and Genovese, P. (2021). Gene editing of hematopoietic stem cells: Hopes and hurdles toward clinical translation. Front. Genome Ed. *3*.
28. Genovese, P., Schiroli, G., Escobar, G., Tomaso, T.D., Firrito, C., Calabria, A., Moi, D., Mazzieri, R., Bonini, C., Holmes, M.C., et al. (2014). Targeted genome editing in human repopulating haematopoietic stem cells. Nature *510*, 235–240.
29. Wang, J., Exline, C.M., DeClercq, J.J., Llewellyn, G.N., Hayward, S.B., Li, P.W.L., Shivak, D.A., Surosky, R.T., Gregory, P.D., Holmes, M.C., and Cannon, P.M. (2015). Homology-driven genome editing in hematopoietic stem and progenitor cells using ZFN mRNA and AAV6 donors. Nat. Biotechnol. *33*, 1256–1263.
30. Dever, D.P., Bak, R.O., Reinisch, A., Camarena, J., Washington, G., Nicolas, C.E., Pavel-Dinu, M., Saxena, N., Wilkens, A.B., Mantri, S., et al. (2016). CRISPR/Cas9 beta-globin gene targeting in human haematopoietic stem cells. Nature *539*, 384–389.
31. Schiroli, G., Ferrari, S., Conway, A., Jacob, A., Capo, V., Albano, L., Plati, T., Castiello, M.C., Sanvito, F., Gennery, A.R., et al. (2017). Preclinical modeling highlights the therapeutic potential of hematopoietic stem cell gene editing for correction of SCID-X1. Sci. Transl. Med. *9*, eaan0820.
32. Kuo, C.Y., et al. (2018). Site-specific gene editing of human hematopoietic stem cells for X-Linked Hyper-IgM syndrome. Cell Rep *23*, 2606–2616.
33. Pavel-Dinu, M., Wiebking, V., Dejene, B.T., Srifa, W., Mantri, S., Nicolas, C.E., Lee, C., Bao, G., Kildebeck, E.J., Punjya, N., et al. (2019). Gene correction for SCID-X1 in long-term hematopoietic stem cells. Nat. Commun. *10*, 1634.
34. Rai, R., et al. (2020). Targeted gene correction of human hematopoietic stem cells for the treatment of Wiskott - Aldrich Syndrome. Nat. Commun. *11*, 4034.

35. Schiroli, G., Conti, A., Ferrari, S., Della Volpe, L., Jacob, A., Albano, L., Beretta, S., Calabria, A., Vavassori, V., Gasparini, P., et al. (2019). Precise gene editing preserves hematopoietic stem cell function following transient p53-mediated DNA damage response. Cell Stem Cell *24*, 551–565.e8.

36. Ingo, D.M., et al. (2016). Bone marrow-derived CD34(-) fraction: a rich source of mesenchymal stromal cells for clinical application. Cytotherapy *18*, 1560–1563.

37. Fares, I., et al. (2014). Cord blood expansion. Pyrimidoindole derivatives are agonists of human hematopoietic stem cell self-renewal. Science *345*, 1509–1512.

38. Lin, H., Damen, J.E., Walasek, M.A., Szilvassy, S.J., Turhan, A.G., Louis, S.A., Eaves, A.C., and Wognum, A.W. (2020). Feeder-free and serum-free in vitro assay for measuring the effect of drugs on acute and chronic myeloid leukemia stem/progenitor cells. Exp. Hematol. *90*, 52–64.e11.

39. Scala, S., and Aiuti, A. (2019). In vivo dynamics of human hematopoietic stem cells: novel concepts and future directions. Blood Adv. *3*, 1916–1924.

40. Sumide, K., Matsuoka, Y., Kawamura, H., Nakatsuka, R., Fujioka, T., Asano, H., Takihara, Y., and Sonoda, Y. (2018). A revised road map for the commitment of human cord blood CD34-negative hematopoietic stem cells. Nat. Commun. *9*, 2202.

41. Notta, F., Doulatov, S., Laurenti, E., Poeppl, A., Jurisica, I., and Dick, J.E. (2011). Isolation of single human hematopoietic stem cells capable of long-term multilineage engraftment. Science *333*, 218–221.

42. Laurenti, E., and Göttgens, B. (2018). From haematopoietic stem cells to complex differentiation landscapes. Nature *553*, 418–426.

43. Lombardo, A., Cesana, D., Genovese, P., Di Stefano, B., Provasi, E., Colombo, D.F., Neri, M., Magnani, Z., Cantore, A., Lo Riso, P., et al. (2011). Site-specific integration and tailoring of cassette design for sustainable gene transfer. Nat. Methods *8*, 861–869.

44. Ferrari, S., Jacob, A., Beretta, S., Unali, G., Albano, L., Vavassori, V., Cittaro, D., Lazarevic, D., Brombin, C., Cugnata, F., et al. (2020). Efficient gene editing of human long-term hematopoietic stem cells validated by clonal tracking. Nat. Biotechnol. *38*, 1298–1308.

45. Ferrari, S., Beretta, S., Jacob, A., Cittaro, D., Albano, L., Merelli, I., Naldini, L., and Genovese, P. (2021). BAR-Seq clonal tracking of gene-edited cells. NatNat. Protoc. *16*, 2991–3025.

46. Scala, S., Basso-Ricci, L., Dionisio, F., Pellin, D., Giannelli, S., Salerio, F.A., Leonardelli, L., Cicalese, M.P., Ferrua, F., Aiuti, A., and Biasco, L. (2018). Dynamics of genetically engineered hematopoietic stem and progenitor cells after autologous transplantation in humans. Nat. Med. *24*, 1683–1690.

47. Gartel, A.L. (2006). Is p21 an oncogene? Mol. Cancer Ther. *5*, 1385–1386.

48. Bernardo, M.E., and Fibbe, W.E. (2013). Mesenchymal stromal cells: sensors and switchers of inflammation. Cell Stem Cell *13*, 392–402.

49. Di Micco, R., Krizhanovsky, V., Baker, D., and d'Adda di Fagagna, F. (2020). Cellular senescence in ageing: from mechanisms to therapeutic opportunities. Nat. Rev. Mol. Cell Biol. https://doi.org/10.1038/s41580-020-00314-w.

50. King, K.Y., and Goodell, M.A. (2011). Inflammatory modulation of HSCs: viewing the HSC as a foundation for the immune response. Nat. Rev. Immunol. *11*, 685–692.

51. Chen, J., Astle, C.M., and Harrison, D.E. (2002). Hematopoietic stem cell functional failure in interleukin-2-deficient mice. J. Hematother Stem Cell Res. *11*, 905–912.

52. Tie, R., Li, H., Cai, S., Liang, Z., Shan, W., Wang, B., Tan, Y., Zheng, W., and Huang, H. (2019). Interleukin-6 signaling regulates hematopoietic stem cell emergence. Exp. Mol. Med. *51*, 1–12.

53. Sugiyama, T., Kohara, H., Noda, M., and Nagasawa, T. (2006). Maintenance of the hematopoietic stem cell pool by CXCL12-CXCR4 chemokine signaling in bone marrow stromal cell niches. Immunity *25*, 977–988.

54. Bautz, F., Rafii, S., Kanz, L., and Möhle, R. (2000). Expression and secretion of vascular endothelial growth factor-A by cytokine-stimulated hematopoietic progenitor cells. Possible role in the hematopoietic microenvironment. Exp. Hematol. *28*, 700–706.

55. Itkin, T., et al. (2012). FGF-2 expands murine hematopoietic stem and progenitor cells via proliferation of stromal cells, c-Kit activation, and CXCL12 down-regulation. Blood *120*, 1843–1855.

56. Galimi, F., et al. (1994). Hepatocyte growth factor induces proliferation and differentiation of multipotent and erythroid hemopoietic progenitors. J. Cell Biol. *127*, 1743–1754.

57. Shih, C.C., et al. (2000). A secreted and LIF-mediated stromal cell-derived activity that promotes ex vivo expansion of human hematopoietic stem cells. Blood *95*, 1957–1966.

58. Rezzoug, F., Huang, Y., Tanner, M.K., Wysoczynski, M., Schanie, C.L., Chilton, P.M., Ratajczak, M.Z., Fugier-Vivier, I.J., and Ildstad, S.T. (2008). TNF-alpha is critical to facilitate hemopoietic stem cell engraftment and function. J. Immunol. *180*, 49–57.

59. Heo, S.K., et al. (2016). LIGHT (TNFSF14) increases the survival and proliferation of human bone marrow-derived mesenchymal stem cells. PLoS One *11*, e0166589.

60. Chen, H., Min, X.H., Wang, Q.Y., Leung, F.W., Shi, L., Zhou, Y., Yu, T., Wang, C.M., An, G., Sha, W.H., and Chen, Q.K. (2015). Pre-activation of mesenchymal stem cells with TNF-alpha, IL-1beta and nitric oxide enhances its paracrine effects on radiation-induced intestinal injury. Sci. Rep. *5*, 8718.

61. Silberstein, L., et al. (2016). Proximity-based differential single-cell analysis of the niche to identify stem/progenitor cell Regulators. Cell Stem Cell *19*, 530–543.

62. Eljaafari, A., Tartelin, M.L., Aissaoui, H., Chevrel, G., Osta, B., Lavocat, F., and Miossec, P. (2012). Bone marrow-derived and synovium-derived mesenchymal cells promote Th17 cell expansion and activation through caspase 1 activation: contribution to the chronicity of rheumatoid arthritis. Arthritis Rheum. *64*, 2147–2157.

63. Crane, G.M., Jeffery, E., and Morrison, S.J. (2017). Adult haematopoietic stem cell niches. Nat. Rev. Immunol. *17*, 573–590.

64. Yamashita, M., and Passegue, E. (2019). TNF-Alpha Coordinates hematopoietic stem cell survival and myeloid regeneration. Cell Stem Cell *25*, 357–372.e357.

65. Luis, T.C., et al. (2016). Inflammatory signals in HSPC development and homeostasis: Too much of a good thing? Exp. Hematol. *44*, 908–912.

66. Sezaki, M., et al. (2020). Immuno-Modulation of hematopoietic stem and progenitor cells in inflammation. Front Immunol. *11*, 585367.

67. Schinke, C., et al. (2015). IL8-CXCR2 pathway inhibition as a therapeutic strategy against MDS and AML stem cells. Blood *125*, 3144–3152.

68. Block, G.J., et al. (2009). Multipotent stromal cells are activated to reduce apoptosis in part by upregulation and secretion of stanniocalcin-1. Stem Cells *27*, 670–681.

69. Nakahara, F., et al. (2019). Engineering a haematopoietic stem cell niche by revitalizing mesenchymal stromal cells. Nat. Cell Biol *21*, 560–567.

70. Iyer, S.S., and Rojas, M. (2008). Anti-inflammatory effects of mesenchymal stem cells: novel concept for future therapies. Expert Opin. Biol. Ther. *8*, 569–581.

71. Ciccocioppo, R., et al. (2011). Autologous bone marrow-derived mesenchymal stromal cells in the treatment of fistulising Crohn's disease. Gut *60*, 788–798.

72. Le Blanc, K., et al. (2008). Mesenchymal stem cells for treatment of steroid-resistant, severe, acute graft-versus-host disease: a phase II study. Lancet *371*, 1579–1586.

73. Németh, K., Leelahavanichkul, A., Yuen, P.S.T., Mayer, B., Parmelee, A., Doi, K., Robey, P.G., Leelahavanichkul, K., Koller, B.H., Brown, J.M., et al. (2009). Bone marrow stromal cells attenuate sepsis via prostaglandin E(2)-dependent reprogramming of host macrophages to increase their interleukin-10 production. Nat. Med. *15*, 42–49.

74. Csaszar, E., Kirouac, D.C., Yu, M., Wang, W., Qiao, W., Cooke, M.P., Boitano, A.E., Ito, C., and Zandstra, P.W. (2012). Rapid expansion of human hematopoietic stem cells by automated control of inhibitory feedback signaling. Cell Stem Cell *10*, 218–229.

75. Redondo-Castro, E. (2017). Interleukin-1 primes human mesenchymal stem cells towards an anti-inflammatory and pro-trophic phenotype in vitro. Stem Cell Res. Ther. *8*, 79.

76. Lin, W., Oh, S.K., Choo, A.B., and George, A.J. (2012). Activated T cells modulate immunosuppression by embryonic-and bone marrow-derived mesenchymal stromal cells through a feedback mechanism. Cytotherapy *14*, 274–284.

77. Jang, J., Suh, S., and Kim, J. (2014). Enhanced Ido activity and reduced il8 production of hbm-msc in inflammatory condition by pretreatment with cytokines including interferon-gamma. Cytotherapy *16*, S66–S67.

78. Crippa, S., Santi, L., Berti, M., De Ponti, G., and Bernardo, M.E. (2021). Role of ex vivo expanded mesenchymal stromal cells in determining hematopoietic stem cell transplantation outcome. Front. Cell Dev. Biol. *9*, 663316.

79. Nauta, A.J., and Fibbe, W.E. (2007). Immunomodulatory properties of mesenchymal stromal cells. Blood *110*, 3499–3506.

80. Kang, Y.G., Jeong, J.Y., Lee, T.H., Lee, H.S., and Shin, J.W. (2018). Synergistic integration of mesenchymal stem cells and Hydrostatic Pressure in the expansion and maintenance of human hematopoietic/progenitor cells. Stem Cells Int. *2018*, 4527929.

81. Teixeira, F.G., and Salgado, A.J. (2020). Mesenchymal stem cells secretome: current trends and future challenges. Neural Regen. Res. *15*, 75–77.

YMTHE, Volume *31*

Supplemental Information

Mesenchymal stromal cells improve the transplantation outcome of CRISPR-Cas9 gene-edited human HSPCs

Stefania Crippa, Anastasia Conti, Valentina Vavassori, Samuele Ferrari, Stefano Beretta, Silvia Rivis, Roberto Bosotti, Serena Scala, Stefania Pirroni, Raisa Jofra-Hernandez, Ludovica Santi, Luca Basso-Ricci, Ivan Merelli, Pietro Genovese, Alessandro Aiuti, Luigi Naldini, Raffaella Di Micco, and Maria Ester Bernardo

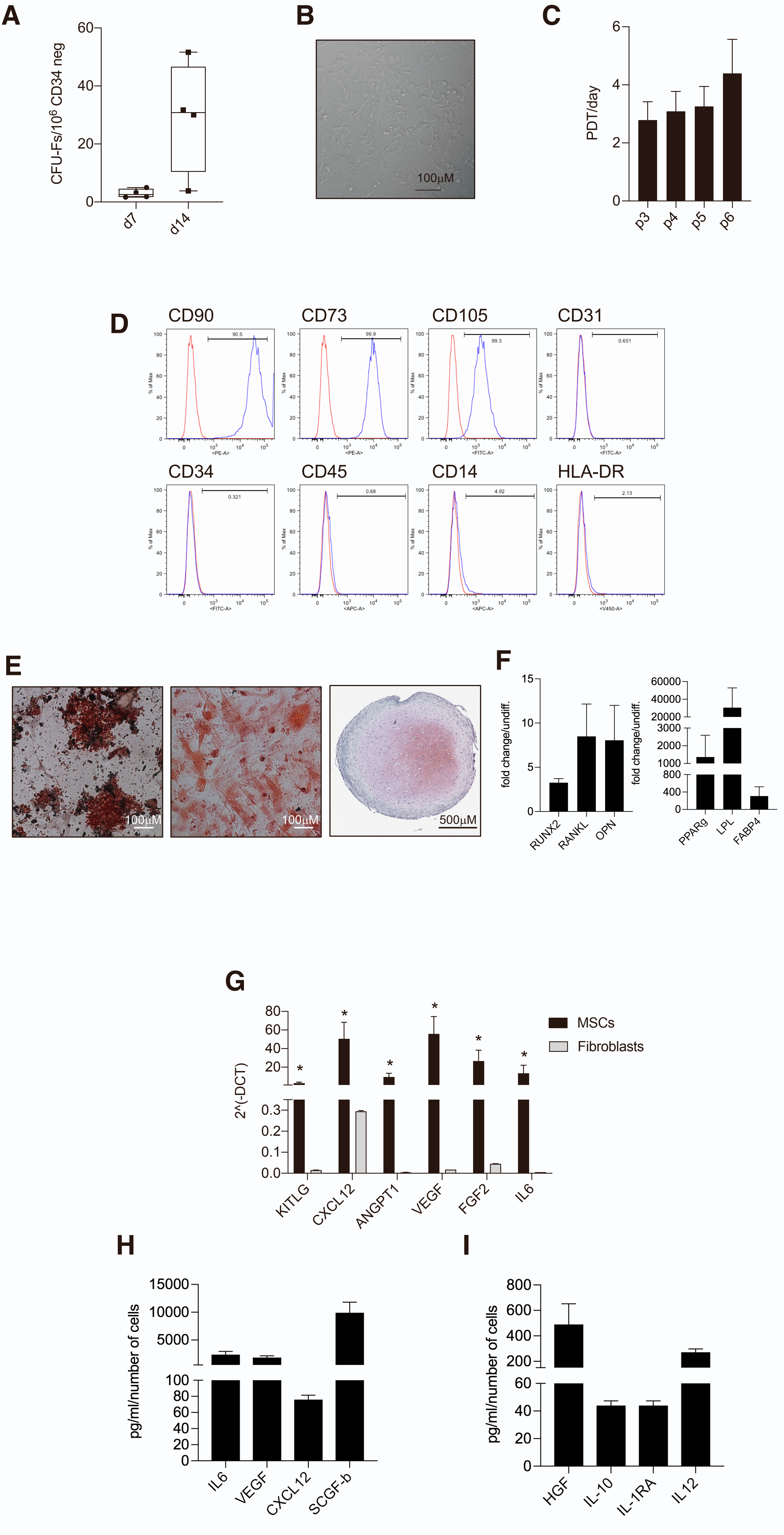

A
CFU-Fs/10^6 CD34 neg
d7
d14
B
100μM
C
PDT/day
p3
p4
p5
p6
D
CD90
90.5
CD73
99.9
CD105
99.3
CD31
0.651
CD34
0.321
CD45
0.68
CD14
4.92
HLA-DR
2.13
% of Max
<PE-A>
<FITC-A>
<APC-A>
<V450-A>
E
100μM
100μM
500μM
F
fold change/undiff.
RUNX2
RANKL
OPN
PPARg
LPL
FABP4
G
2^(-DCT)
MSCs
Fibroblasts
KITLG
CXCL12
ANGPT1
VEGF
FGF2
IL6
H
pg/ml/number of cells
IL6
VEGF
CXCL12
SCGF-b
I
pg/ml/number of cells
HGF
IL-10
IL-1RA
IL12

Figure S1

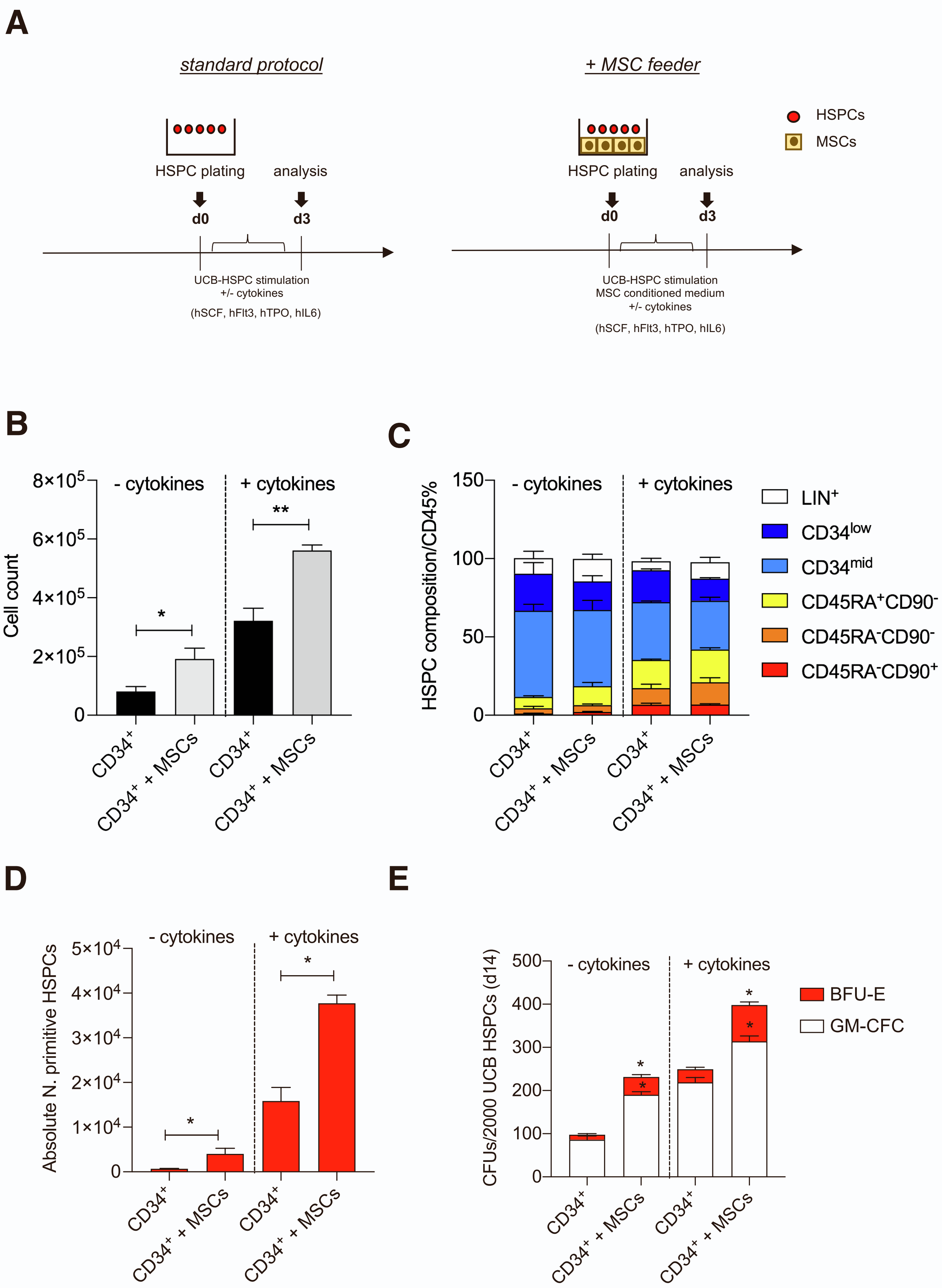

A
standard protocol
+ MSC feeder
HSPCs
MSCs
HSPC plating
analysis
d0
d3
UCB-HSPC stimulation
+/- cytokines
(hSCF, hFlt3, hTPO, hIL6)
UCB-HSPC stimulation
MSC conditioned medium
+/- cytokines
(hSCF, hFlt3, hTPO, hIL6)
B
Cell count
- cytokines
+ cytokines
CD34+
CD34+ + MSCs
C
HSPC composition/CD45%
LIN+
CD34low
CD34mid
CD45RA+CD90-
CD45RA-CD90-
CD45RA-CD90+
D
Absolute N. primitive HSPCs
E
CFUs/2000 UCB HSPCs (d14)
BFU-E
GM-CFC

Figure S2

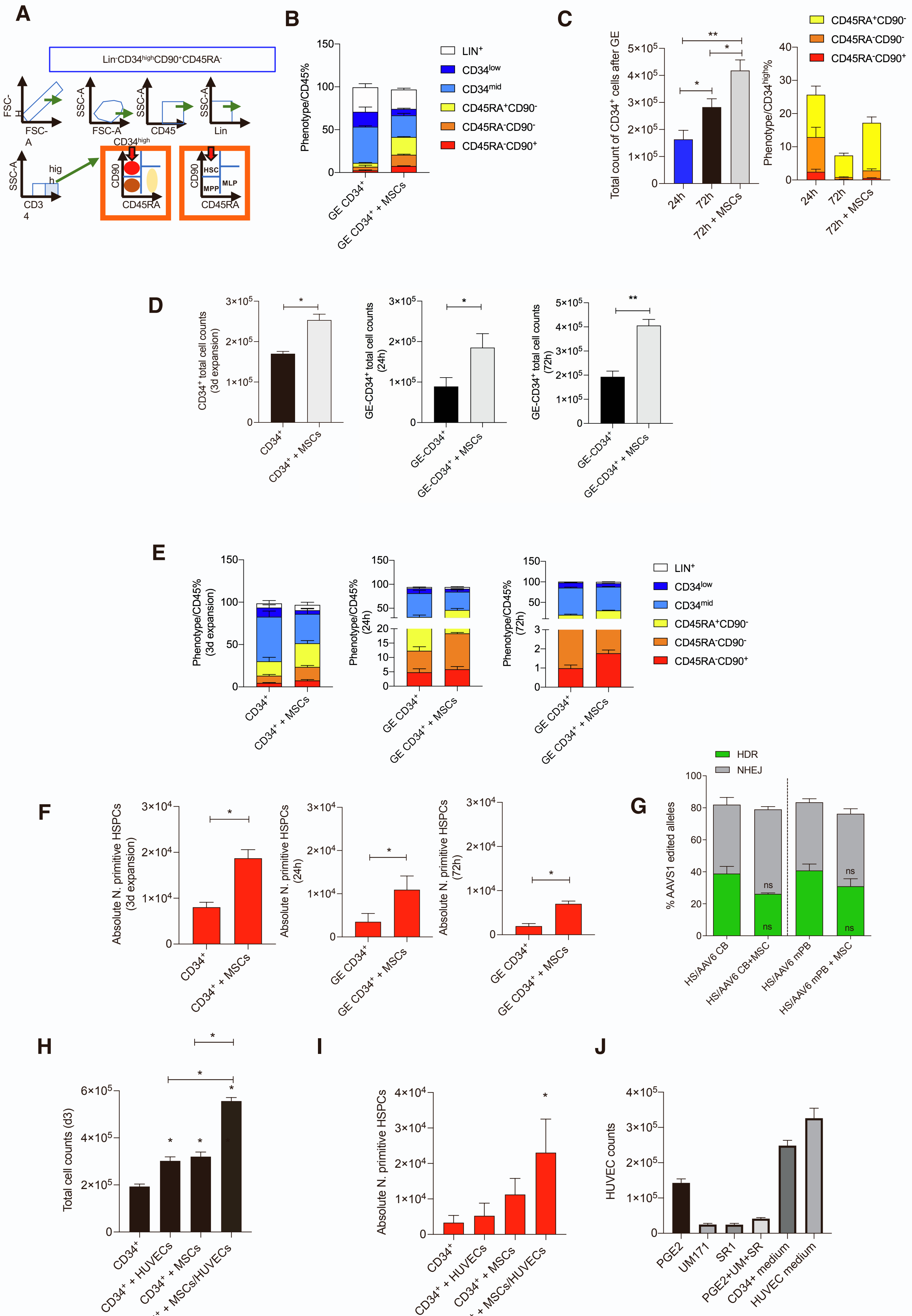
A
Lin-CD34highCD90+CD45RA-
FSC-H
FSC-A
SSC-A
CD45
Lin
CD34high
CD34
high
CD90
CD45RA
HSC
MPP
MLP
B
Phenotype/CD45%
LIN+
CD34low
CD34mid
CD45RA+CD90-
CD45RA-CD90-
CD45RA-CD90+
GE CD34+
GE CD34+ + MSCs
C
Total count of CD34+ cells after GE
Phenotype/CD34high%
24h
72h
72h + MSCs
D
CD34+ total cell counts (3d expansion)
GE-CD34+ total cell counts (24h)
GE-CD34+ total cell counts (72h)
CD34+
CD34+ + MSCs
GE-CD34+
GE-CD34+ + MSCs
E
Phenotype/CD45% (3d expansion)
Phenotype/CD45% (24h)
Phenotype/CD45% (72h)
F
Absolute N. primitive HSPCs (3d expansion)
Absolute N. primitive HSPCs (24h)
Absolute N. primitive HSPCs (72h)
G
HDR
NHEJ
% AAVS1 edited alleles
ns
HS/AAV6 CB
HS/AAV6 CB+MSC
HS/AAV6 mPB
HS/AAV6 mPB + MSC
H
Total cell counts (d3)
CD34+ + HUVECs
CD34+ + MSCs/HUVECs
I
Absolute N. primitive HSPCs
J
HUVEC counts
PGE2
UM171
SR1
PGE2+UM+SR
CD34+ medium
HUVEC medium

Figure S3

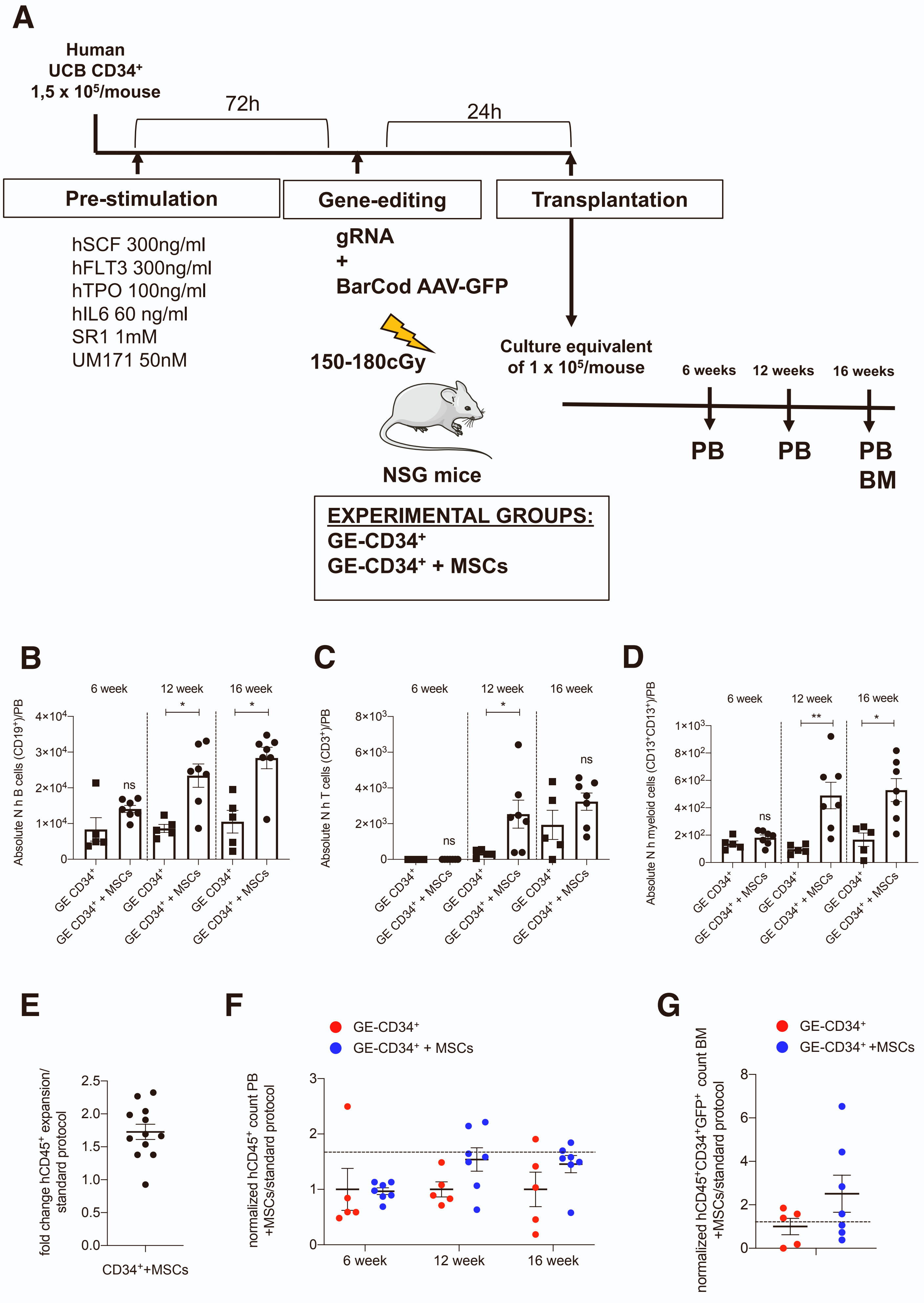
A
Human UCB CD34+ 1,5 x 10^5/mouse
72h
24h
Pre-stimulation
Gene-editing
Transplantation
hSCF 300ng/ml
hFLT3 300ng/ml
hTPO 100ng/ml
hIL6 60 ng/ml
SR1 1mM
UM171 50nM
gRNA
+
BarCod AAV-GFP
150-180cGy
NSG mice
Culture equivalent of 1 x 10^5/mouse
6 weeks
12 weeks
16 weeks
PB
PB
PB
BM
EXPERIMENTAL GROUPS:
GE-CD34+
GE-CD34+ + MSCs
B
Absolute N h B cells (CD19+)/PB
C
Absolute N h T cells (CD3+)/PB
D
Absolute N h myeloid cells (CD13+CD13+)/PB
6 week
12 week
16 week
ns
GE CD34+
GE CD34+ + MSCs
E
fold change hCD45+ expansion/ standard protocol
CD34++MSCs
F
GE-CD34+
GE-CD34+ + MSCs
normalized hCD45+ count PB +MSCs/standard protocol
G
GE-CD34+
GE-CD34+ +MSCs
normalized hCD45+CD34+GFP+ count BM +MSCs/standard protocol

Figure S4

A

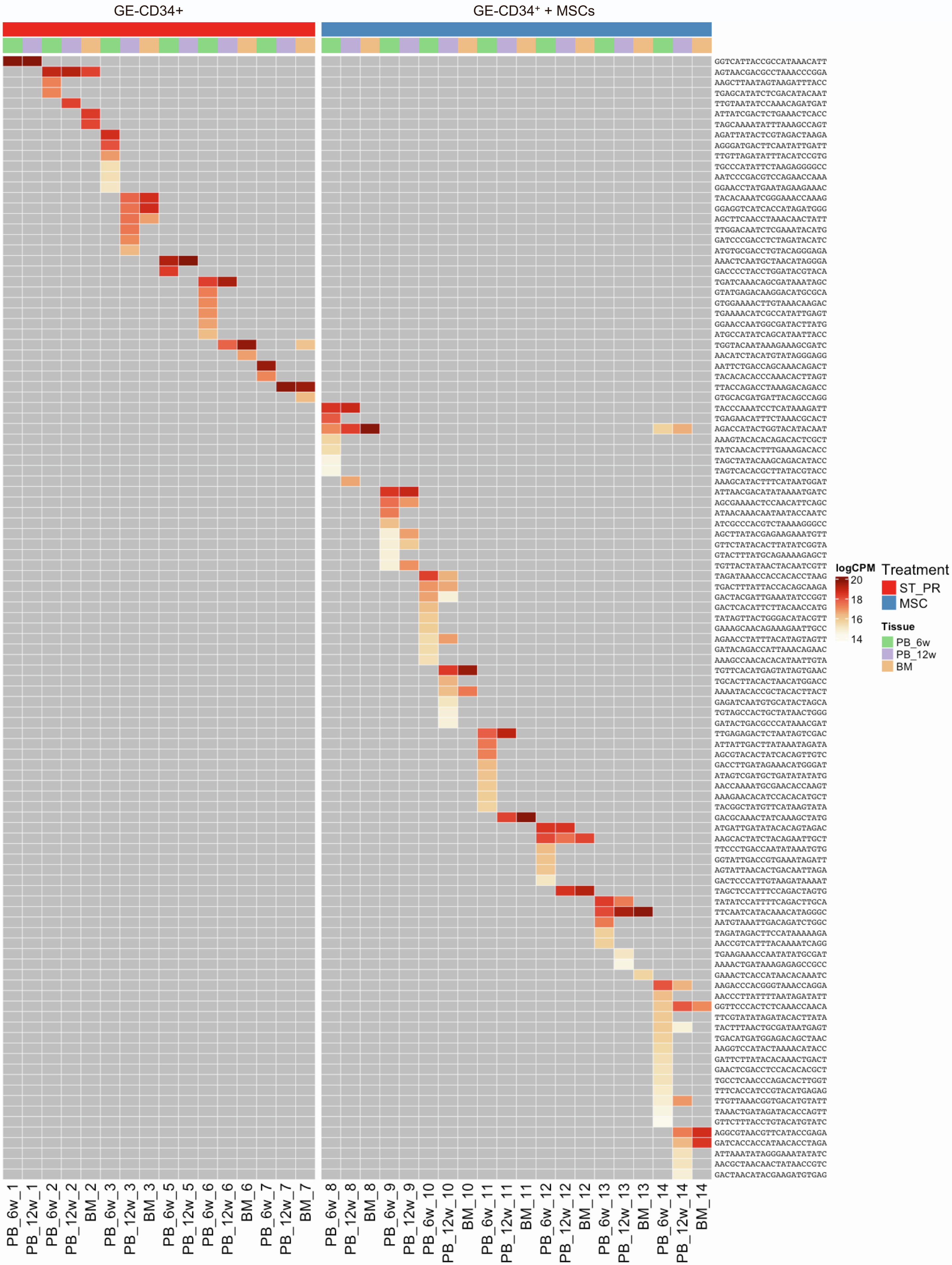

GE-CD34+
GE-CD34⁺ + MSCs
GGTCATTACCGCCATAAACATT
AGTAACGACGCCTAAACCCGGA
AAGCTTAATAGTAAGATTTACC
TGAGCATATCTCGACATACAAT
TTGTAATATCCAAACAGATGAT
ATTATCGACTCTGAAACTCACC
TAGCAAAATATTTAAAGCCAGT
AGATTATACTCGTAGACTAAGA
AGGGATGACTTCAATATTGATT
TTGTTAGATATTTACATCCGTG
TGCCCATATTCTAAGAGGGGCC
AATCCCGACGTCCAGAACCAAA
GGAACCTATGAATAGAAGAAAC
TACACAAATCGGGAAACCAAAG
GGAGGTCATCACCATAGATGGG
AGCTTCAACCTAAACAACTATT
TTGGACAATCTCGAAATACATG
GATCCCGACCTCTAGATACATC
ATGTGCGACCTGTACAGGGAGA
AAACTCAATGCTAACATAGGGA
GACCCCTACCTGGATACGTACA
TGATCAAACAGCGATAAATAGC
GTATGAGACAAGGACATGCGCA
GTGGAAAACTTGTAAACAAGAC
TGAAAACATCGCCATATTGAGT
GGAACCAATGGCGATACTTATG
ATGCCATATCAGCATAATTACC
TGGTACAATAAAGAAAGCGATC
AACATCTACATGTATAGGGAGG
AATTCTGACCAGCAAACAGACT
TACACACACCCAAACACTTAGT
TTACCAGACCTAAAGACAGACC
GTGCACGATGATTACAGCCAGG
TACCCAAATCCTCATAAAGATT
TGAGAACATTTCTAAACGCACT
AGACCATACTGGTACATACAAT
AAAGTACACACAGACACTCGCT
TATCAACACTTTGAAAGACACC
TAGCTATACAAGCAGACATACC
TAGTCACACGCTTATACGTACC
AAAGCATACTTTCATAATGGAT
ATTAACGACATATAAAATGATC
AGCGAAAACTCCAACATTCAGC
ATAACAAACAATAATACCAATC
ATCGCCCACGTCTAAAAGGGCC
AGCTTATACGAGAAGAAATGTT
GTTCTATACACTTATATCGGTA
GTACTTTATGCAGAAAAGAGCT
TGTTACTATAACTACAATCGTT
TAGATAAACCACCACACCTAAG
TGACTTTATTACCACAGCAAGA
GACTACGATTGAAATATCCGGT
GACTCACATTCTTACAACCATG
TATAGTTACTGGGACATACGTT
GAAAGCAACAGAAAGAATTGCC
AGAACCTATTTACATAGTAGTT
GATACAGACCATTAAACAGAAC
AAAGCCAACACACATAATTGTA
TGTTCACATGAGTATAGTGAAC
TGCACTTACACTAACATGGACC
AAAATACACCGCTACACTTACT
GAGATCAATGTGCATACTAGCA
TGTAGCCACTGCTATAACTGGG
GATACTGACGCCCATAAACGAT
TTGAGAGACTCTAATAGTCGAC
ATTATTGACTTATAAATAGATA
AGCGTACACTATCACAGTTGTC
GACCTTGATAGAAACATGGGAT
ATAGTCGATGCTGATATATATG
AACCAAAATGCGAACACCAAGT
AAAGAACACATCCACACATGCT
TACGGCTATGTTCATAAGTATA
GACGCAAACTATCAAAGCTATG
ATGATTGATATACACAGTAGAC
AAGCACTATCTACAGAATTGCT
TTCCCTGACCAATATAAATGTG
GGTATTGACCGTGAAATAGATT
AGTATTAACACTGACAATTAGA
GACTCCCATTGTAAGATAAAAT
TAGCTCCATTTCCAGACTAGTG
TATATCCATTTTCAGACTTGCA
TTCAATCATACAAACATAGGGC
AATGTAAATTGACAGATCTGGC
TAGATAGACTTCCATAAAAAGA
AACCGTCATTTACAAAATCAGG
TGAAGAAACCAATATATGCGAT
AAAACTGATAAAGAGAGCCGCC
GAAACTCACCATAACACAAATC
AAGACCCACGGGTAAACCAGGA
AACCCTTATTTTAATAGATATT
GGTTCCCACTCTCAAACCAACA
TTCGTATATAGATACACTTATA
TACTTTAACTGCGATAATGAGT
TGACATGATGGAGACAGCTAAC
AAGGTCCATACTAAAACATACC
GATTCTTATACACAAACTGACT
GAACTCGACCTCCACACACGCT
TGCCTCAACCCAGACACTTGGT
TTTCACCATCCGTACATGAGAG
TTGTTAAACGGTGACATGTATT
TAAACTGATAGATACACCAGTT
GTTCTTTACCTGTACATGTATC
AGGCGTAACGTTCATACCGAGA
GATCACCACCATAACACCTAGA
ATTAAATATAGGGAAATATATC
AACGCTAACAACTATAACCGTC
GACTAACATACGAAGATGTGAG
PB_6w_1
PB_12w_1
PB_6w_2
PB_12w_2
BM_2
PB_6w_3
PB_12w_3
BM_3
PB_6w_5
PB_12w_5
PB_6w_6
PB_12w_6
BM_6
PB_6w_7
PB_12w_7
BM_7
PB_6w_8
PB_12w_8
BM_8
PB_6w_9
PB_12w_9
PB_6w_10
PB_12w_10
BM_10
PB_6w_11
PB_12w_11
BM_11
PB_6w_12
PB_12w_12
BM_12
PB_6w_13
PB_12w_13
BM_13
PB_6w_14
PB_12w_14
BM_14
logCPM
20
18
16
14
Treatment
ST_PR
MSC
Tissue
PB_6w
PB_12w
BM

Figure S5

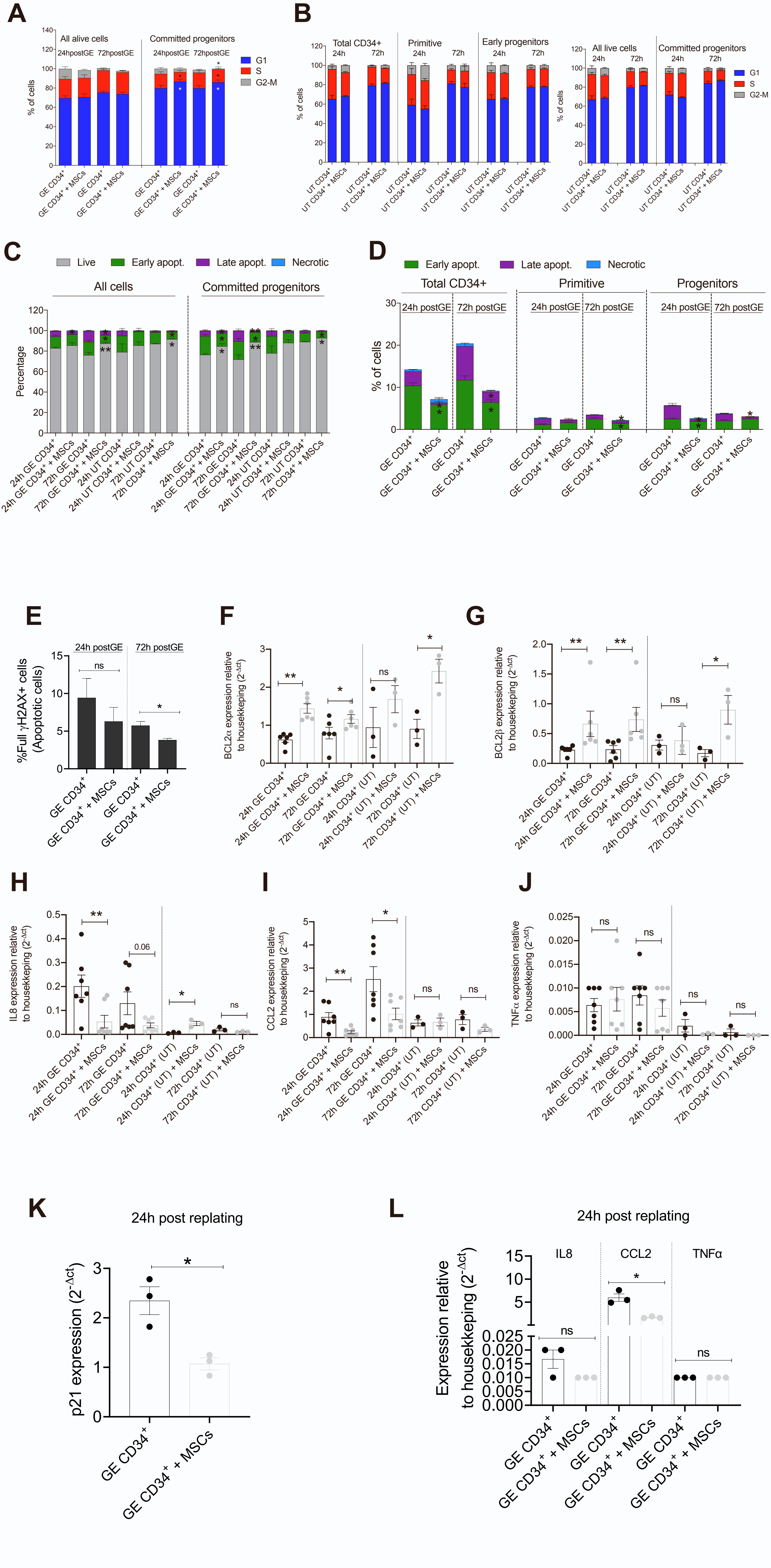
A
All alive cells
Committed progenitors
24hpostGE
72hpostGE
% of cells
G1
S
G2-M
GE CD34+
GE CD34+ + MSCs
B
Total CD34+
Primitive
Early progenitors
All live cells
Committed progenitors
24h
72h
UT CD34+
UT CD34+ + MSCs
C
Live
Early apopt.
Late apopt.
Necrotic
All cells
Committed progenitors
Percentage
24h GE CD34+
24h GE CD34+ + MSCs
72h GE CD34+
72h GE CD34+ + MSCs
24h UT CD34+
24h UT CD34+ + MSCs
72h UT CD34+
72h CD34+ + MSCs
D
Total CD34+
Primitive
Progenitors
24h postGE
72h postGE
% of cells
E
%Full γH2AX+ cells (Apoptotic cells)
ns
F
BCL2α expression relative to housekeeping (2-Δct)
G
BCL2β expression relative to housekeeping (2-Δct)
24h CD34+ (UT)
24h CD34+ (UT) + MSCs
72h CD34+ (UT)
72h CD34+ (UT) + MSCs
H
IL8 expression relative to housekeeping (2-Δct)
0.06
I
CCL2 expression relative to housekeeping (2-Δct)
J
TNFα expression relative to housekeeping (2-Δct)
K
24h post replating
p21 expression (2-Δct)
L
24h post replating
IL8
CCL2
TNFα
Expression relative to housekeeping (2-Δct)

Figure S6

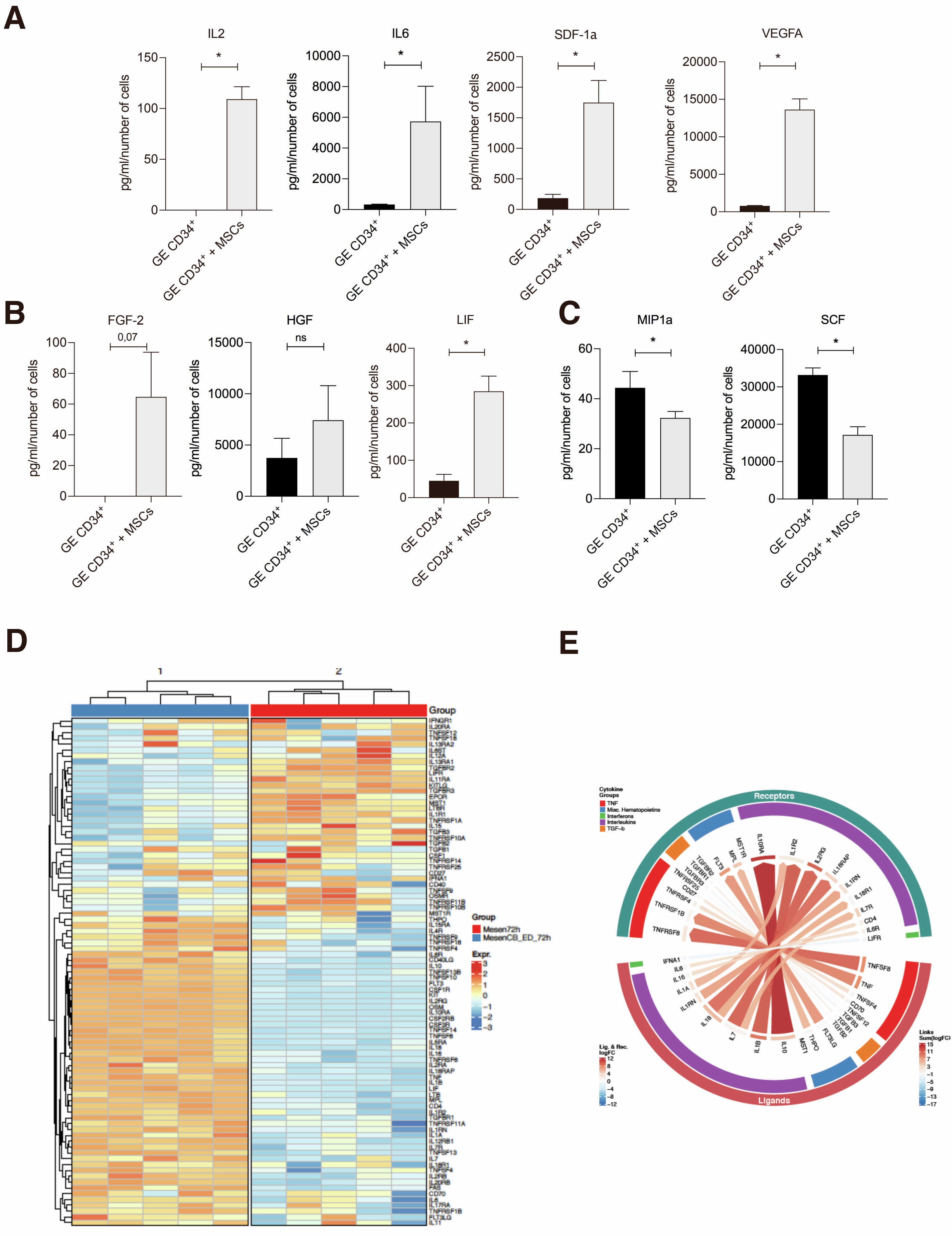
A
IL2
IL6
SDF-1a
VEGFA
pg/ml/number of cells
GE CD34⁺
GE CD34⁺ + MSCs
*
150
100
50
0
10000
8000
6000
4000
2000
2500
2000
1500
1000
500
20000
15000
10000
5000
B
FGF-2
HGF
LIF
0,07
ns
80
60
40
20
400
300
200
C
MIP1a
SCF
40000
30000
D
E
Group
Mesen72h
MesenCB_ED_72h
Expr.
Cytokine Groups
TNF
Misc. Hematopoietins
Interferons
Interleukins
TGF–b
Receptors
Ligands
Lig. & Rec. logFC
Links Sum(logFC)

Figure S7

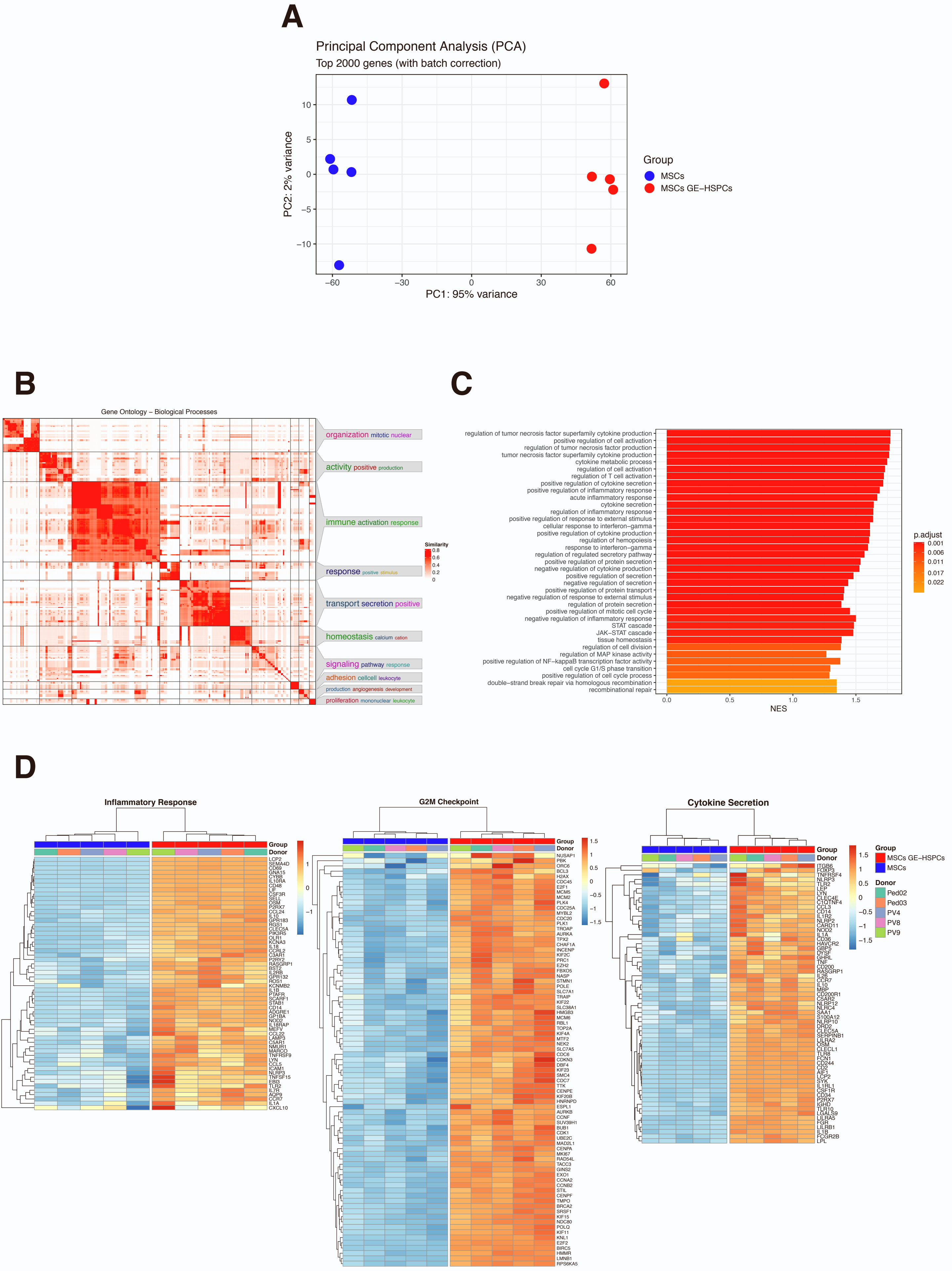
A
Principal Component Analysis (PCA)
Top 2000 genes (with batch correction)
PC2: 2% variance
PC1: 95% variance
10
5
0
−5
−10
−60
−30
0
30
60
Group
MSCs
MSCs GE−HSPCs
B
Gene Ontology – Biological Processes
organization mitotic nuclear
activity positive production
immune activation response
response positive stimulus
transport secretion positive
homeostasis calcium cation
signaling pathway response
adhesion cellcell leukocyte
production angiogenesis development
proliferation mononuclear leukocyte
Similarity
0.8
0.6
0.4
0.2
0
C
regulation of tumor necrosis factor superfamily cytokine production
positive regulation of cell activation
regulation of tumor necrosis factor production
tumor necrosis factor superfamily cytokine production
cytokine metabolic process
regulation of cell activation
regulation of T cell activation
positive regulation of cytokine secretion
positive regulation of inflammatory response
acute inflammatory response
cytokine secretion
regulation of inflammatory response
positive regulation of response to external stimulus
cellular response to interferon−gamma
positive regulation of cytokine production
regulation of hemopoiesis
response to interferon−gamma
regulation of regulated secretory pathway
positive regulation of protein secretion
negative regulation of cytokine production
positive regulation of secretion
negative regulation of secretion
positive regulation of protein transport
negative regulation of response to external stimulus
regulation of protein secretion
positive regulation of mitotic cell cycle
negative regulation of inflammatory response
STAT cascade
JAK−STAT cascade
tissue homeostasis
regulation of cell division
regulation of MAP kinase activity
positive regulation of NF−kappaB transcription factor activity
cell cycle G1/S phase transition
positive regulation of cell cycle process
double−strand break repair via homologous recombination
recombinational repair
0.0
0.5
1.0
1.5
NES
p.adjust
0.001
0.006
0.011
0.017
0.022
D
Inflammatory Response
G2M Checkpoint
Cytokine Secretion
Group
Donor
Group
MSCs GE−HSPCs
MSCs
Donor
Ped02
Ped03
PV4
PV8
PV9

Figure S8

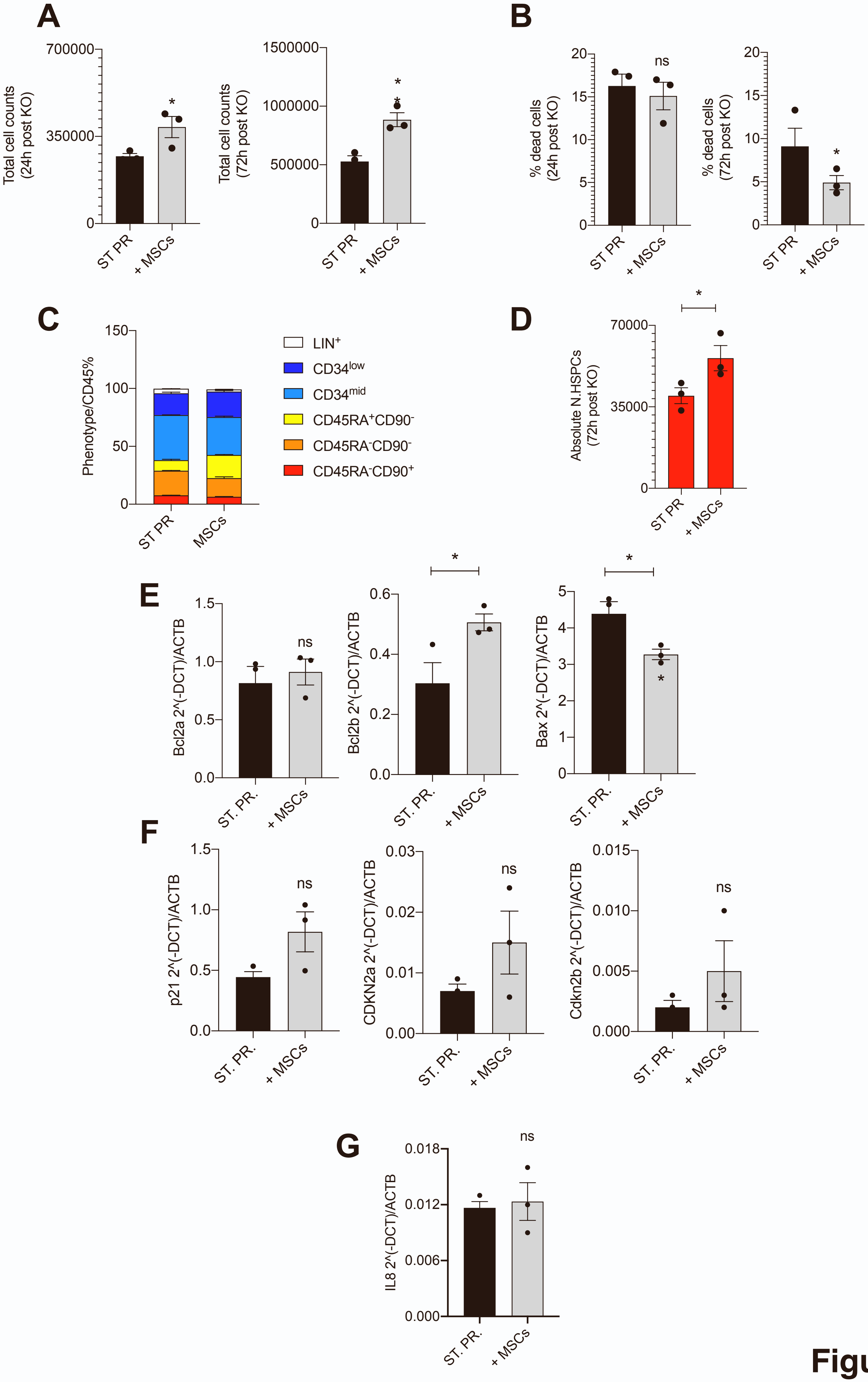
A
Total cell counts (24h post KO)
700000
350000
0
ST PR
+ MSCs
*
Total cell counts (72h post KO)
1500000
1000000
500000
0
ST PR
+ MSCs
*
B
% dead cells (24h post KO)
20
15
10
5
0
ns
ST PR
+ MSCs
% dead cells (72h post KO)
*
ST PR
+ MSCs
C
Phenotype/CD45%
150
100
50
0
ST PR
MSCs
LIN+
CD34low
CD34mid
CD45RA+CD90-
CD45RA-CD90-
CD45RA-CD90+
D
Absolute N.HSPCs (72h post KO)
70000
35000
0
*
ST PR
+ MSCs
E
Bcl2a 2^(-DCT)/ACTB
1.5
1.0
0.5
0.0
ns
ST. PR.
+ MSCs
Bcl2b 2^(-DCT)/ACTB
0.6
0.4
0.2
0.0
*
ST. PR.
+ MSCs
Bax 2^(-DCT)/ACTB
5
4
3
2
1
0
*
ST. PR.
+ MSCs
F
p21 2^(-DCT)/ACTB
1.5
1.0
0.5
0.0
ns
ST. PR.
+ MSCs
CDKN2a 2^(-DCT)/ACTB
0.03
0.02
0.01
0.00
ns
ST. PR.
+ MSCs
Cdkn2b 2^(-DCT)/ACTB
0.015
0.010
0.005
0.000
ns
ST. PR.
+ MSCs
G
IL8 2^(-DCT)/ACTB
0.018
0.012
0.006
0.000
ns
ST. PR.
+ MSCs

Figure S9

Supplementary Figure Legend

Fig. S1. MSC isolation and characterization.

Fig. S2. MSCs support the expansion of UCB-HSPCs while preserving the HSPC subset with a primitive phenotype.

Fig. S3. Phenotypic analysis of *ex-vivo* expanded UCB-HSPCs.

Fig. S4. Analysis of hematological reconstitution in transplanted NSG mice.

Fig. S5. Analysis of clonal reconstitution dynamics in transplanted NSG mice.

Fig. S6. Analysis of cell-cycle, survival and inflammatory state in GE-HSPCs.

Fig. S7. Cytokine analysis of conditioned medium from MSCs co-cultured with GE-HSPCs.

Fig. S8. RNA sequencing analysis of MSCs compared to MSCs co-cultured with GE-HSPCs.

Fig. S9. MSCs in support of KO-edited HSPCs.

Fig. S1: Isolation and characterization of BM-MSCs. A) Clonogenic capacity of BM-MSCs used in this study, determined as CFU-Fs at 7 and 14 days after plating the CD34 negative cellular fraction of healthy donor BM aspirates. Each error bars show means ± s.e.m of CFU-Fs normalized for the number of plated cells. n=6. **B**) Representative picture of *ex-vivo* expanded MSCs acquiring a spindle-like morphology in culture. **C**) Proliferation capacity of MSCs evaluated as population doubling time (PDT)/day starting from passage 3. Each error bars show means ± s.e.m of PDT calculated as (https://www.doubling-time.com/compute.php). **D**) Representative flow cytometer analysis used to characterize the expression of canonical MSC markers (CD90, CD73, CD105) and lack of expression of hematopoietic (CD34, CD45, CD14), MHC class II (HLA-DR) and endothelial markers (CD31) in the MSC samples used in this study. **E**) Representative pictures of MSCs differentiated into osteoblasts (Alizarin red, left panel), adipocytes (Oil Red O, middle panel) and chondrocytes (Safranin O-Fast Green, right panel). **F**) RT-qPCR expression analysis of early (*PPARγ*) and late genes (LPL, FABP4) of adipogenic differentiation in MSCs cultured in proper adipogenic differentiation medium for 21 days. Expression analysis of early (*RUNX2*) and late (*RANKL*, *OPN*) genes of osteogenic differentiation in MSCs differentiated for 21 days in osteogenic medium in reported in the right panel. Each error bars show means ± s.e.m gene expression calculated as 2 -ΔCT relative to Actin-beta (*ACTB*) gene (left panel). **G**) Fold change of hematopoietic supportive gene expression in BM-derived MSCs (black bars) compared to fibroblasts (grey bars) expanded in culture for 3-4 passages. Each error bars show means ± s.e.m. of gene expression calculated as 2 -ΔCT relative to *ACTB* gene expression. Each error bars show means ± s.e.m (n ≥ 3). *p*-values were determined by Mann-Whitney test (*p ≤ 0.05). (n =7). Quantification (pg/ml) of hematopoietic supportive cytokines (**H**) and inflammatory molecules (**I**) in the conditioned medium from MSCs expanded for 72 hours in culture. Each error bars show means ± s.e.m. normalized for the number of cells originally plated (n = 6).

Fig. S2. MSCs support the expansion of UCB-HSPCs while preserving the HSPC subset with a primitive phenotype. A) Schematic representation of the cell culture protocol used to expand UCB-HSPCs on MSC feeder. HSPCs expanded on plastic using standard protocols [5] were used as controls. **B**) Human HSPC ($CD34^{+}$) total cell count after 72 hours of culture with (+ cytokines) or without (-cytokines) the addition of proper cytokines in the medium (hSCF, hFLT3, hTPO, hIL-6) [5]. Grey bars: UCB-HSPCs co-cultured with MSCs; black bars: UCB-HSPCs cultured in plastic dishes. Each error bars show means ± s.e.m. of total cell count (n=5). *p*-values were determined by Mann-Whitney test (*$p \leq 0.05$; **$p \leq 0.001$). **C**) Phenotypic composition of UCB-HSPCs expanded for 72 hours in culture with (+ cytokines) or without (-cytokines) the addition of proper cytokines in the medium on MSC feeder ($CD34^{+}$ + MSCs) or according to standard protocols ($CD34^{+}$). The primitive HSPC subset was phenotypically defined as $CD45^{+}$, $CD34^{high}$, $CD90^{+}$, $CD45RA^{-}$ subset. Each error bars show means ± s.e.m. of percentage of each subset to the percentage of human $CD45^{+}$ cells (n = 4). **D**) Absolute number of HSPCs with a primitive phenotype present in culture after 72 hours of expansion (n=4). Absolute number was calculated as percentage of $CD45^{+}$, $CD34^{high}$, $CD90^{+}$, $CD45RA^{-}$ of total cell counts (**B**). Each error bars show means ± s.e.m. *p*-values were determined by Mann-Whitney test (*$p \leq 0.05$). **E**) CFUs assay 14 days after plating *ex vivo* expanded UCB-HSPCs into methylcelluose medium. Red = Burst Forming Units-Erythroid; white = granulocyte-macrophage progenitors. Each error bars show means ± s.e.m. (n = 4). *p*-values were determined by Mann-Whitney test (*$p \leq 0.05$).

Fig. S3. Phenotypic analysis of *ex-vivo* expanded UCB-HSPCs. A) Schematic representation of the gating strategy developed to phenotypically identify primitive HSPC in culture. **B**) Phenotypic analysis of GE-HSPCs expanded on MSC feeder for 72h (GE $CD34^{+}$ + MSCs) after gene-editing. GE-HSPCs expanded on plastic were used as controls (GE $CD34^{+}$). Each error bars show means ± s.e.m. of the percentage of each phenotypic subset on human $CD45^{+}$ cells (n=4). **C**) Total cell count

(left panel) and phenotypic analysis (right panel) of the primitive HSPC subset in UCB-HSPCs cultured for 72 hours upon gene editing in the presence or absence of an MSC feeder compared to GE-HPSCs recovered on plastic for 24 hours. **D**) Total cell counts of UCB-HSPCs co-cultured with MSCs ($CD34^+$ + MSCs) for 3 days before editing (left panel). Total cell counts of GE-HSPCs recovered in culture for 24 hours and expanded for 72 hours after editing in the presence (GE $CD34^+$ + MSCs) or absence (GE $CD34^+$) of MSC feeder are shown in the middle and right panel, respectively. For each condition, the phenotypic analysis of cultured $CD34^+$ is reported in **E**. **F**) Absolute number of HSPCs with a primitive phenotype ($CD45^+$, $CD34^{high}$, $CD90^+$, $CD45RA^-$) before editing (left panel), at 24 hours after editing (middle panel) and 72 hours after editing (right panel). The absolute number of HSPCs with a primitive phenotype in co-culture with MSCs (grey bars) was compared to the ones observed using our standard protocol (black bars) of gene-editing. **G**) Percentage of HDR and NHEJ 72 hours after editing UCB (CB) and mobilized peripheral blood (mPB) $CD34^+$ cells edited in the presence or not of MSCs (n = 3); Wilcoxon matched-pairs signed rank test. Total cell counts (**H**) and absolute number of phenotypically primitive HSPCs (**I**) after 72-hour co-culture of $CD34^+$ cells in the presence of MSCs, HUVEC, and mixed MSC-HUVEC feeder. $CD34^+$ cells expanded on plastic were used as controls. **J**) HUVEC cell counts 72 hours after the exposure to the gene-editing drugs required by our protocol for efficient gene transfer. For all the experiments, each error bars show means ± s.e.m ($n \geq 3$). *p*-values were determined by Mann-Whitney test (*$p \leq 0.05$).

Fig. S4. Flow cytometry analysis of hematological reconstitution in NSG transplanted mice. A) Schematic representation of the experimental plan used for the transplantation of the culture outgrowing cells of UCB-HSPCs gene editing in the presence of MSCs (GE CD34+ + MSCs) or according to standard protocol (GE CD34+) into NSG mice. Absolute number of human B cells ($CD19^+$) (**B**), T cells ($CD3^+$) (**C**) and myeloid cells ($CD13^+$, $CD33^+$) (**D**) on total $hCD45^+$ cells in the peripheral blood (PB) of NSG mice transplanted with HSPCs gene-edited in the presence of MSCs

(+MSCs) or according to standard protocol (st.pr) at different time points post transplantation. **E**) Ratio between the number of outgrowing cells effectively transplanted upon gene editing in the presence of MSCs and the number of cells gene-edited according to our standard protocol. **F**) Fold change of human engraftment in the peripheral blood of transplanted mice calculated as ratio of human $CD45^+$ cell counts normalized for the number of effectively transplanted cells in mice treated with GE-HPSCs + MSCs compared to standard GE-HSPC transplants. **G**) Fold change of $CD45^+CD34^+GFP^+$ cell engraftment in the BM of transplanted mice represented as ratio of $CD45^+CD34^+GFP^+$ absolute counts normalized for the number of transplanted cells in mice transplanted with GE-HSPCs+MSCs compared to standard GE-HSPCs. For all plots, individual data points represent an animal. *p*-values were determined by Mann-Whitney test (*$p \leq 0.05$;** $p \leq 0.01$).

Fig. S5. Clonal tracking analysis. A) Heatmap showing the logCPM abundance (red-scaled palette) of dominant unique "barcode" sequences (rows) in the PB at 6 and 12 weeks after transplantation and in the BM at sacrifice of mice transplanted with HSPCs gene-edited in presence of MSCs or standard GE-HSPCs.

Fig. S6. Characterization of the DDR response in GE-HSPCs. A) Percentage of committed progenitors in the indicated cell-cycle phase at 24 ($n = 8$) and 72 hours ($n = 7$) after gene-editing. Significance was calculated for each time point comparing GE $CD34^+$ + MSCs to GE $CD34^+$ at each cell-cycle phase. Each error bars show means ± s.e.m. p-values were determined by Mann-Whitney test (only significant comparisons were reported). **B**) Cell-cycle analysis at the indicated time points in untreated (UT) and gene-edited (GE) committed progenitors. Live: Annexin V^-, $7AAD^-$, Early apoptotic: Annexin V^+, $7AAD^-$, Late apoptotic: Annexin V^+, $7AAD^+$, Necrotic: Annexin V^-, $7AAD^+$ ($n \geq 3$ for each time point). Each error bars show means ± s.e.m. p-values were determined by Mann-

Whitney test for each apoptotic phase (only significant comparisons were reported; *$p < 0.05$). **C**) Apoptosis analysis at the indicated time points in untreated (UT) and gene-edited (GE) committed progenitors. Live: Annexin V^-, $7AAD^-$, Early apoptotic: Annexin V^+, $7AAD^-$, Late apoptotic: Annexin V^+, $7AAD^+$, Necrotic: Annexin V^-, $7AAD^+$ ($n \geq 3$ for each time point). Each error bars show means ± s.e.m. p-values were determined by Mann-Whitney test for each apoptotic phase (only significant comparisons were reported; *$p < 0.05$). **D**) Apoptosis analysis at the indicated time points in the different subsets of gene-edited (GE) HSPCs. Early apoptotic: Annexin V+, 7AAD-, Late apoptotic: Annexin V+, 7AAD+, Necrotic: Annexin V-, 7AAD+ ($n \geq 3$ for each time point). Each error bars show means ± s.e.m. p-values were determined by Mann-Whitney test for each apoptotic phase (only significant comparisons were reported; *$p < 0.05$). **E**) Quantification of full-stained γH2AX positive cells from Fig. 3A-C. (n=4 for each time point). Each error bars show means ± s.e.m. p-values were determined by Mann-Whitney test comparing GE $CD34^+$ + MSCs to GE $CD34^+$ (ns $p>0.05$; *$p < 0.05$). RT-qPCR expression analysis of *BCL2α* (**F**) and *BCL2β* (**G**) in untreated (UT) and gene-edited HSPCs in the presence or absence of MSC feeder after 24 and 72 hours of culture. RT-qPCR expression analysis of *IL-8* (**H**), *CCL2* (**I**) and *TNFα* (**J**) in untreated (UT) and gene-edited (GE) HSPCs co-cultured with MSCs (+ MSCs) for 24 and 72 hours compared to our standard protocol condition. Expression analysis of *p21* (**K**), *IL-8*, *CCL2*, and *TNFα* (**L**) in GE-HSPCs maintained in culture for 24 hours in a medium poor of cytokines after 72 hour-expansion in the presence (GE $CD34^+$ + MSCs) or absence (GE $CD34^+$) of MSCs. For all the experiments, error bars show means ± s.e.m. and the level of gene expression was calculated as 2 -ΔCT relative to *GUSB* gene ($n \geq 3$ for each time point). p-values were determined by Mann-Whitney test comparing GE $CD34^+$ + MSCs to GE $CD34^+$ and comparing UT $CD34^+$ + MSCs to UT $CD34^+$(ns $p>0.05$; *$p < 0.05$).

Fig. S7. Cytokine analysis of the conditioned from the co-culture of GE-HSPCs with MSCs. Quantification (pg/ml) of hematopoietic supportive cytokines (**A**) and growth factors (**B**) in the

conditioned medium from MSCs co-cultured with GE-HSPCs for 72 hours. Conditioned medium from GE-HSPCs expanded in plastic dishes for 72 hours was used as control. Quantification was normalized for the number of plated cells. MIP1a and SCF were the only two factors downregulated in the co-culture medium (**C**). Each error bars show means ± s.e.m. (n=4). p-values were determined by Mann-Whitney test (*$p \leq 0.05$). **D**) Heatmap representation of gene expression analysis of cytokines in MSCs (n=5) and MSC co-cultured with GE-HSPCs (n=5) with unsupervised clustering showing the separation of the two groups. **E**) Chord diagram showing the results of the DGE analysis on ligand-receptor cytokine pairs. For each pair, the logFC values resulting from the comparison of MSC with MSC co-cultured with GE-HSPCs samples are reported, while links represent the score of the pairs computed by summing the logFC values of both ligands and receptors. Cytokine pairs are organized into groups based on their function: TNF, Miscellaneous Hematopoietins, Interferons, Interleukins, and TGF-β.

Fig. S8. RNA sequencing analysis of MSCs compared to MSCs co-cultured with GE-HSPCs. A) Principal component analysis (PCA) plot of the RNA-seq samples. **B**) Heatmap showing the similarity of significant Gene Ontology (BP) terms resulting from the Gene Set Enrichment Analysis (GSEA). **C**) Bar plot of the inflammation, cell cycle, and cytokine production GO terms with the corresponding NES values. Colors represent adjusted p-values. **D**) Heatmap of regularized log-normalized read counts of GSEA core-enriched genes of the inflammatory response hallmark category (left panel). Heatmap of regularized log-normalized read counts of GSEA core-enriched genes of the G2M checkpoint hallmark category (middle panel). Heatmap of regularized log-normalized read counts of GSEA core-enriched genes of the cytokine secretion category (right panel).

Fig. S9. MSCs in support of KO-edited HSPCs. Total cell count (**A**) and percentage of death (**B**) of KO-edited HSPCs recovered for 24 (left panel) and 72 hours (right panel) on a MSC feeder

(+MSCs) or on plastic according to our standard protocol (ST. PR.). Phenotypic analysis (**C**) and absolute number of phenotypically primitive (**D**) KO-HSPCs 72 hours after editing in the presence (+MSCs) or absence (ST. PR.) of MSCs. **E-G**) Gene expression analysis of pro-survival (*BCL2*α, *BCL2*β), apoptotic (*BAX*), cell-cyle (*p21*, *CDKN2A*, *CDKN2B*) and inflammatory genes (*IL-8*) in KO-edited HSPCs recovered in culture for 72 hours after editing on a MSC feeder or according to our standard protocol conditions. For all the experiments, each error bars show means ± s.e.m ($n \geq 3$). *p*-values were determined by Mann-Whitney test (*$p \leq 0.05$).

Supplementary Tables

Table S1. List of differentially expressed gene (DGEs) in MSCs exposed to GE-HSPCs compared to MSCs.

Table S2. List of the significantly enriched GO terms derived from the GSEA of the pre-ranked gene list resulting from the comparison between MSCs and MSCs co-cultured with GE-HSPCs.

Supplementary Materials

Material and Methods.

Cell Culture. BM-MSCs were cultured in DMEM+GlutaMAX (Thermo Fisher Scientific, 10566-016) supplemented with 5% platelet lysate (Stemcell, 06963) and 1% penicillin/streptomycin (Gibco, 15140122). UCB $CD34^+$ cells (Lonza, 2C-101) were cultured in Stemspan-SFEM II (Stemcell Technologies, 09650), supplemented with 1% penicillin/streptomycin, 2% L-glutamine (Gibco, A2916801), human recombinant thrombopoietin (TPO) (20ng/ml) (PeproTech, 300-18), interleukin-6 (IL-6) (20ng/ml) (PeproTech, 200-06), Flt3 ligand (Flt3L) (100 ng/ml) (PeproTech, 300-19) and stem cell factor (SCF) (100 ng/ml) (PeproTech, 300-07). PGE2 (10 ug/ml) (Cayman, 14750) was added to facilitate the recovering of thawed cells. After editing, pyrimidoindole derivative (UM171) (50nM) (Stemcell, 72912) and StemRegenin 1 (SR1) (1 uM) (Biovision, 1967) were added as supplement in Stemspan medium. HUVEC cells were cultured in M199 medium (ThermoFisher Scientific, 11150059) supplemented with 20% fetal bovine serum (FBS) (ThermoFisher Scientific, A4766801), 1% penicillin/streptomycin, 2 mM L-glutamine, 10 mM 4-(2-hydroxyethyl)-1-piperazineethanesulfonic acid (HEPES) (ThermoFisher Scientific, 15630080), 50 mg/ml heparin (Stemcell Technologies, 07980) and 50 mg/ml endothelial cell growth supplement (ECGS) (Merck, E2759).

Fibroblast colony-forming unit assay. The CD34 negative fraction of BM mononuclear cells was plated at a density of 2×10^5 cells/cm^2 in DMEM+GlutaMAX supplemented with 5% platelet lysate and 1% penicillin/streptomycin. Colony-forming units were stained with 1% crystal violet (Sigma-Aldrich, catalog C0775) and manually counted after 7 and 14 days.

Population doubling time. BM-MSCs were plated at a density of 3 × 10^4 cells/cm^2, detached, and counted when reaching 80%–90% of confluence using Trypan blue to distinguish live cells. Proliferative capacity was calculated as population doubling time according to http://www.doubling-time.com/ compute.php. Cell proliferation was followed from passage 3 to 6.

Flow cytometry MSC characterization. MSCs were immunophenotypically characterized by flow-cytometry (FC) at passage 2/3 to evaluate the expression of MSC canonical markers. Cells were detached and washed with PBS + 2% FBS. 1 x 10^5 cells were incubated with the proper antibody mix for 10 minutes at RT in the dark. The following antibodies were used: CD90 PE (BioLegend, 328110), CD105 FITC (BioLegend, 323204), CD73 PE (BD Biosciences 555445), CD146 V450 (BD Biosciences, 562136), CD271 PE (BD Biosciences, 557196), CD45 APC (BD Biosciences, 340910), CD34 FITC (BD Biosciences, 345801), CD14 APC (BD Biosciences, 555399), CD31 FITC (BD Biosciences, 55027) and HLA-DR PB (BioLegend, 307624). Unstained cells were used as negative control. All samples were run on BD FACSCanto II cytometer (BD Biosciences). At least 10.000 were recorded.

***In vitro* adipogenic, osteogenic and chondrogenic differentiation of BM-MSCs.** For adipogenic and osteogenic differentiation, BM-MSCs were cultured in proper induction medium according to a previously published protocol [1]. Adipogenic differentiation medium: alpha MEM (Thermo Fisher Scientific, 12571), 1% penicillin/streptomycin, and 10% MesenCult MSC Stimulatory Supplement (Stemcell Technologies, 05402), supplemented with 10^{-10} M dexamethasone (Sigma, D4902), 50 µg/ml l-ascorbic acid (Sigma, A92902), 10 µg/ml insulin, 5 µM 3-isobutyl-1-methylxanthine (Sigma, I5879), 0.2^{-10} M indomethacin (Sigma, I7378), and 0.5 mM β-glycerol

phosphate (Sigma, G9422). Osteogenic differentiation medium: alpha MEM, 1% penicillin/streptomycin, and 10% MesenCult MSC Stimulatory Supplement, supplemented with 10^{-10} M dexamethasone and 50 μg/ml l-ascorbic acid. Starting from day 7 of differentiation, 5 mM β-glycerol phosphate was added to the medium. Chondrogenic differentiation of MSCs was obtained by plating MSCs at a concentration of $2.5x10^5$ cells/ml in StemMACS ChondroDiff Media (MiltenyiBiotech, 130-091-679), following the manufacturer's instruction. Detection of chondrocytes was performed on PFA 4% fixed pellets using Safranin O-Fast Green (Sigma, 84120). MSC differentiation was evaluated after 21 days by proper staining and/or gene expression analysis.

Oil Red O staining. BM-MSC-differentiated cells, were fixed with PFA 4% for 15' (Sigma, 1004968350), washed with Isopropanol 60% and stained with Oil Red 0 solution (Merck, 102419) for 15' to evaluate the efficiency of adipogenic differentiation. After washing with H_20, cells were stained with hematoxylin solution for 10' (Sigma, MHS80).

Alizarin Red staining. BM-MSC -differentiated cells, were fixed with PFA 4% for 15' (Sigma, 1004968350), washed with H_20 and stained with Alizarin Red (Merck, TMS-008-C) for 15' to evaluate the efficieny of osteogenic differentiation. After washing with H_20, cells were stained with hematoxylin solution for 10' (Sigma, MHS80).

RNA extraction. RT-PCR and real-time PCR. Total RNA was extracted using RNeasy Micro Kit (Qiagen, 74004) or miRNeasy Micro Kit (Qiagen, 217084), according to the manufacturer's instructions and DNase treatment was performed using RNase-free DNase Set (Qiagen,79254). cDNA was synthesized from 1 μg total RNA using the high-capacity reverse transcription kit (Applied Biosystems, 4319983) or from 30 ng total RNA using iScript cDNA Synthesis Kit (Bio-

Rad, 170-8891) and then pre-amplified using TaqMan PreAmp Master Mix (2X) (ThermoFisher, 4488593). SYBR Green based quantitative PCR was performed using QuantiFast SYBR Green PCR Kit (Qiagen, 1039712) or Fast SYBR Green Master Mix (Thermofisher, 4385618), starting from 10 ng of cDNA with a Viia7 real-time PCR system (Thermofisher). The following primers were used after standard curve method optimization to reach the 100% primer efficiency:

**Gene**	**Primers**
KITLG	FW 5'AGCCAGCTCCCTTAGGAATG 3'<br>RW 5' GACTTGGCTGTCTCTTCTTCCA 3'
CXCL12	FW 5' TGCCCTTCAGATTGTAGCCC 3'<br>RW 5' CGAGTGGGTCTAGCGGAAAG 3'
FGF2	FW 5'GCTGTACTGCAAAAACGGGG 3'<br>RW 5' TAGCTTGATGTGAGGGTCGC 3'
IL6	FW 5'GATGGATGCTTCCAATCTGG 3'<br>RW 5' TGTTCTGGAGGTACTCTAGG 3'
VEGFA	FW 5' CATCTTCAAGCCATCCTGTG 3'<br>RW 5' GGAAGCTCATCTCTCCTATG 3'
ANGPT1	FW 5' ACATGGGCAATGTGCCTACA 3'<br>RW 5' TCTCAAGTTTTTGCAGCCACTG 3'
PPARg	FW 5' TCAGAAATGCCTTGCAGTGG 3'<br>RW 5' TATCACTGGAGATCTCCGCCAA 3'
FABP4	FW 5' AAACTGGTGGTGGAATGCGT 3'<br>RW 5' GCGAACTTCAGTCCAGGTCA 3'
LPL	FW 5' CCGCCGACCAAAGAAGAGAT 3'<br>RW 5' TAGCCACGGACTCTGCTACT 3'
RUNX2	FW 5' CCGGAATGCCTCTGCTGTTA 3'<br>RW 5' TGTCTGTGCCTTCTGGGTTC 3'
RANKL	FW 5' GACACAACTCTGGAGAGTCA 3'<br>RW 5' TCTGCTCTGATGTGCTGTGATC 3'
OPN	FW 5' TAGGCATCACCTGTGCCATA 3'<br>RW 5' CTCAGAACTTCCAGAATCAGCC 3'
IL8	FW 5' CACCGGAAGGAACCATCTCA 3'<br>RW 5' TGGCAAAACTGCACCTTCACA 3'
TNF	FW 5' CCTGCTGCACTTTGGAGTGA 3'<br>RW 5' GAGGGTTTGCTACAACATGGG 3'
CCL2	FW 5' AGAATCACCAGCAGCAAGTGTCC 3'<br>RW 5' TCCTGAACCCACTTCTGCTTGG 3'
BAX	FW 5' CCCCGAGAGGTCTTTTTCCG 3'<br>RW 5' TGGTTCTGATCAGTTCCGGC 3'

CDKN1A	FW 5’ CAGCATGACAGATTTCTACCACTC 3’<br>RW 5’ CTCGCGCTTCCAGGACTG 3’
CDKN2A	FW 5’ CACCAGCGTGTCCAGGAAG 3’<br>RW 5’ CAACTGCGCCGACCCC 3’
CDKN2B	FW 5’ CAGACGACCCCAGGCATC 3’<br>RW 5’ CTGCCACTCTCACCCGAC 3’
BCLα	FW 5’ CTTTGAGTTCGGTGGGGTCA 3’<br>RW 5’ GGGCCGTACAGTTCCACAAA 3’
BCLβ	FW 5’ GGTGAACTGGGGGAGGATTG3’<br>RW 5’ GCCCAGACTCACATCACCAA 3’
ACTB	FW 5’ ACAGAGCCTCGCCTTTGCC 3’<br>RW 5’ GATATCATCATCCATGGTGAGCTGG 3’
GUSB	FW 5’ CTGACACCTCCAAGTATCCCAAG 3’<br>RW 5’ GTCGTGTACAGAAGTACAGACCGC 3’

Colony-Forming Units (CFUs) assays. Clonogenic capacity of HSPCs and GE HSPCs cultured *in vitro* was evaluated by CFUs assay using methylcellulose-based MethoCult (Stemcell H4434). 500 were plated in 1,1ml of MethoCult in 35mm dish in triplicates for each condition. The number and the type colonies was determined at day 7 or day 14 using the Zeiss Axio Observer inverted microscope.

Luminex assay. The presence of hematopoietic supportive factors and inflammatory cytokines was quantified (pg/ml) in the medium conditioned from BM-MSCs using the Immune Monitoring 65-Plex Human ProcartaPlex™ Panel (EPX650-10065-901) and normalized for the number of cells plated according to the kit assay protocol.

Gene editing of UCB $CD34^+$ HSPCs. UCB $CD34^+$ cells (purchased from Lonza) were seeded at a density of $5x10^5$ cells/ml in serum-free StemSpan medium (StemCell Technologies) supplemented with penicillin, streptomycin, glutamine, 1 mM SR-1(Biovision), 50 nM UM171 (STEMCell Technologies), 10 mM PGE2 added only at the beginning of the culture (Cayman),

and human early-acting cytokines (SCF 100 ng/ml, Flt3-L 100 ng/ml, TPO 20 ng/ml, and IL-6 20 ng/ml; all purchased from Peprotech).

G-CSF mPB CD34+ HSPCs were purified with the CliniMACS CD34 Reagent System (Miltenyi Biotec) from Mobilized Leukopak (AllCells) upon approval by the Ospedale San Raffaele Bioethical Committee (TIGET-HPCT) according to manufacturer's instructions. HSPCs were seeded at the concentration of $5x10^5$ cells/ml in serum-free StemSpan medium (StemCell Technologies) supplemented with 100 IU/ml penicillin, 100 µg/ml streptomycin, 2% glutamine, 300 ng/ml hSCF, 300 ng/ml hFlt3-L, 100 ng/ml hTPO and 10 µM PGE2 (at the beginning of the culture). Culture medium was also supplemented with 1 µM SR1 and 35 nM UM171. HSPCs were cultured in a 5% CO2 humidified atmosphere at 37C. After 3 days of pre-stimulation in early active cytokine medium, HSPCs were washed with PBS and electroporated using P3 Primary Cell 4D-Nucleofector X Kit and program EO-100 (Lonza). Cells were electroporated with 1,25 µM of RNPs. The following gRNA was used: *5' GTCACCAATCCTGTCCCTAGTGG 3'*. Transduction with AAV6 was performed at a dose of 1-2 $x10^4$ vg/cell 15' after electroporation. AAV6 donor templates were generated from a construct containing AAV2 inverted terminal repeats, produced at the TIGEM Vector Core by a triple-transfection method and purified by ultracentrifugation on a cesium chloride gradient. An AAV6 donor template carrying a PGK-GFP-polyA cassette flanked by homology arms for the AAVS1 locus was used for the *in vitro* experiments [2], while a barcoded version of the same vector was used in xenograft experiments to follow the clonal reconstitution dynamics of edited cells, as previously described [3]. In any case, gene editing efficiency was measured by flow cytometry as percentage of GFP+ cells in GE-HSPCs at 24 and 72 hours after electroporation. When indicated, in the absence of AAV6 donor template, HSPCs were gene knockout (KO) by NHEJ.

HDR and NHEJ molecular analysis. For molecular analysis of HDR and NHEJ, genomic DNA was isolated with QIAamp DNA Micro Kit (Qiagen) according to the manufacturer's instructions. HDR-mediated integration was quantified by ddPCR, using primers and probes designed on the junction between the vector sequence and the targeted locus and on control sequence used for normalization (human TTC5 gene) as previously published (2).

Nuclease activity was measured by mismatch-sensitive endonuclease assay by PCR-based amplification of the targeted locus followed by digestion with T7 Endonuclease I (NEB) according to the manufacturer's instructions. Digested DNA fragments were resolved and quantified by capillary electrophoresis on TapeStation (ThermoFisher) according to the manufacturer's instructions.

RNA sequencing. MSCs expanded in culture and MSCs co-cultured with GE-HSPCs for 72 hours were washed two times with PBS, incubated with trypsin and collected for RNA extraction. RNA was extracted using RNeasy Micro Kit (Qiagen, 74004), according to the manufacturer's instructions and DNase treatment was performed using RNase-free DNase Set (Qiagen,79254). RNA quality control was performed on HSRNA chip (Tape Station 2100, Agilent), and only samples with RIN>7 were processed. RNA library preparation was performed using the Illumina Ribo-Zero Plus rRNA Depletion Kit (Illumina) following the manufacturer's instruction. Briefly, starting from 100ng of total RNA, RNA libraries were barcoded, pooled, denatured and diluted to an 0.8 pM final concentration. The SBS (sequencing by synthesis) was performed onto Novaseq 6000 (Illumina) set to 100 cycles, yielding an average of 30-40x106 clusters for each sample in SR mode. Demultiplexing was performed using bcl2fastq. Quality check of RNA-seq sequences was performed using FastQC and low-quality reads were trimmed with Cutadapt. Alignment

against the human reference genome (GRCh38/hg38) was done using STAR, with standard input parameters, and gene counts were produced using Subread featureCounts and Genecode v.34 as gene annotation. Transcript counts were processed with the R/Bioconductor package edgeR, normalizing for library size using trimmed mean of M values and correcting p-values using FDR. Moreover, differential gene expression (DGE) analysis between MSCs and MSCs co-cultured with HSPCs treated for GE was done with the edgeR glmFit function (which fits a negative binomial generalized log-linear model for each gene and conducts genewise statistical tests), and differentially expressed genes (DEGs) were identified accordingly (FDR < 0.01 and |logFC| > 2). Gene Set Enrichment Analysis was performed by the R/Bioconductor package ClusterProfiler on pre-ranked (by logFC) gene lists against the Gene Ontology (GO) – Biological Processes (BP) database.

BAR-Seq amplicon library preparation, sequencing and bioinformatic analyses

Library preparation, purification and sequencing for BAR-Seq were performed as previously described [4]. Barcode sequences were extracted from input FASTQ files using the BAR-Seq bioinformatic pipeline, analyzed with default parameters (edit-distance = 3, min-count = 3, and saturation = 90), and quantified by computing logCPM value

Flow cytometry HSPC characterization. For immunophenotypic analyses (performed on FACSCanto II; BD PharMingen), we used CD90 APC (BD Biosciences, 559869). CD133 PECy7 (Miltenyi Biotec, 130-101-652), CD34 PE (Miltenyi Biotec, 130-081-002). Single stained and Fluorescence Minus One stained cells were used as controls. 7-AAD Viability Staining Solution (BioLegend) were included in the sample preparation for flow cytometry to exclude dead cells

from the analysis. Apoptosis analysis was performed by 7-AAD staining in combination with Annexin V (BioLegend) according to the manufacturer's instructions.

Cell cycle phases analysis by EdU/ Hoechst staining EdU (5-ethynyl-20 -deoxyuridine), supplied with Click-iT EdU Alexa Fluor 647 Imaging Kit (#C10424, Thermo-Fisher Scientific, Waltham, MA, USA), was diluted in DMSO to a final concentration of 10 mM and kept at -20°C. 0.5-1x105 cells were treated with 2mM EdU for 4 hours in culture. Cells (approx. 0,5-1 3 105 cells) were washed with 3 mL of 1% BSA in PBS and fixed with 100 mL of Click-iT fixative for 15 min. Cells were washed again with 3 mL of 1% BSA in PBS and permeabilized with 100 mL of 1X Click-iT saponin-based permeabilization for 15 min. Detection of EdU-DNA was performed by incubating cells with 500 mL of Click-iT Plus reaction cocktail for 30 min at RT protected from light. Cells were subsequently washed with 3 mL of 1% BSA in PBS before staining of DNA with Hoechst for 1h at RT protected from light and immediately after their fluorescence was measured by flow cytometry.

Immunofluorescence Analysis. Multitest slides (10 well, MP Biomedicals) were treated for 20' with Poly-L-lysine solution (Sigma-Aldrich) at 1mg/ml concentration. After two washes with DPBS solution, approximately 0.5/1x105 cells were seeded on covers for 20' and fixed with 4% paraformaldehyde (Santa Cruz Biotechnology) for other 20'. Cells were then permeabilized with 0.1% Triton X-100. After blocking with 0.5% BSA and 0.2% fish gelatin in DPBS, cells were probed with the indicated primary antibodies. After primary antibodies incubation (53BP1 Antibody, Bethyl Laboratories; Anti-phospho Histone H2A.X (Ser139) Antibody, clone JBW301, Merck), cells were washed three times with DPBS and incubate with Alexa 488-, 568- and/or 647-labeled secondary antibodies (Invitrogen). Nuclear DNA was stained with DAPI at 0.2 mg/ml

concentration (Sigma-Aldrich) and covers were mounted with Aqua-Poly/Mount solution (Polysciences. Inc.) on glass slides (Bio-Optica). Fluorescent images were acquired using Leica SP2 and Leica SP5 Confocal microscopes. Quantification of DDR foci in immunofluorescence images was conducted using Cell Profiler (version 2.1.1, revision 6c2d896).

Statistical analyses

Data were expressed as means ± SEM or dot plots with mean values indicated as a line. “n” indicates biologically independent samples/animals/experiments. Mann-Whitney test was performed to compare two independent groups. Analyses were performed using GraphPad Prism v8. Differences were considered statistically significant at $*p < 0.05$, $**p < 0.01$. “ns” indicates not significant comparison.

In our gene-editing experiments we used biological replicates of MSCs derived from healthy donors of the same age range, cultured using a standardized method and co-cultured with HSPCs at a similar *in vitro* passage (p3-p5). For each experiment, we co-cultured MSC biological replicates with a pool of N. 3 UCB-CD34$^+$ cells derived from different healthy donors.

Supplementary References

1. Crippa, S. *et al.* Bone marrow stromal cells from beta-thalassemia patients have impaired hematopoietic supportive capacity. *J Clin Invest* **129**, 1566-1580 (2019).
2. Schiroli, G. *et al.* Precise Gene Editing Preserves Hematopoietic Stem Cell Function following Transient p53-Mediated DNA Damage Response. *Cell Stem Cell* **24**, 551-565 e558 (2019).
3. Ferrari, S. *et al.* Efficient gene editing of human long-term hematopoietic stem cells validated by clonal tracking. *Nat Biotechnol* **38**, 1298-1308 (2020).
4. Ferrari, S. *et al.* BAR-Seq clonal tracking of gene-edited cells. *Nat Protoc* **16**, 2991-3025 (2021).
